# Supplementary material for: Selenonium Salt as a Catalyst for Nucleophilic Substitution Reactions in Water: Synthesis of Thiocyanites and Selenocyanates
Source: Molecules. 2023 Mar 29;28(7):3056. doi: 10.3390/molecules28073056 (PMC10095699; doi:10.3390/molecules28073056)
Supplement: Supplementary file 1 [file molecules-28-03056-s001.zip › molecules-2306244-supplementary.pdf]

Supporting Information for:

Selenonium Salt as a Catalyst for Nucleophilic Substitution Reactions in Water: Synthesis of Thiocyanites and Selenocyanates

TABLE OF CONTENTS

^{1}H NMR of catalyst **C7** and varying amounts of BnBr 02

Optimization conditions for the synthesis of thiocyanates without a catalyst …….. 03

Substrate scope for the preparation of thio- and selenocyanates in uncatalyzed using hydrated EtOH as solvent 04

Copies of ^{1}H and ^{13}C NMR of catalysts and products 05

^{1}H NMR of catalyst **C7** and varying amounts of benzyl bromide.

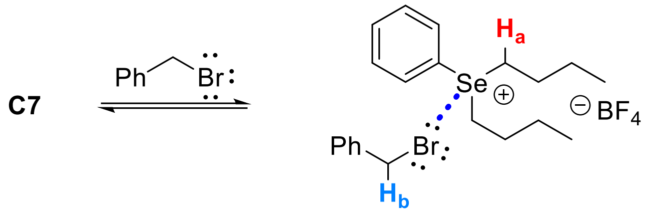

The signal shift of hydrogen H_a:

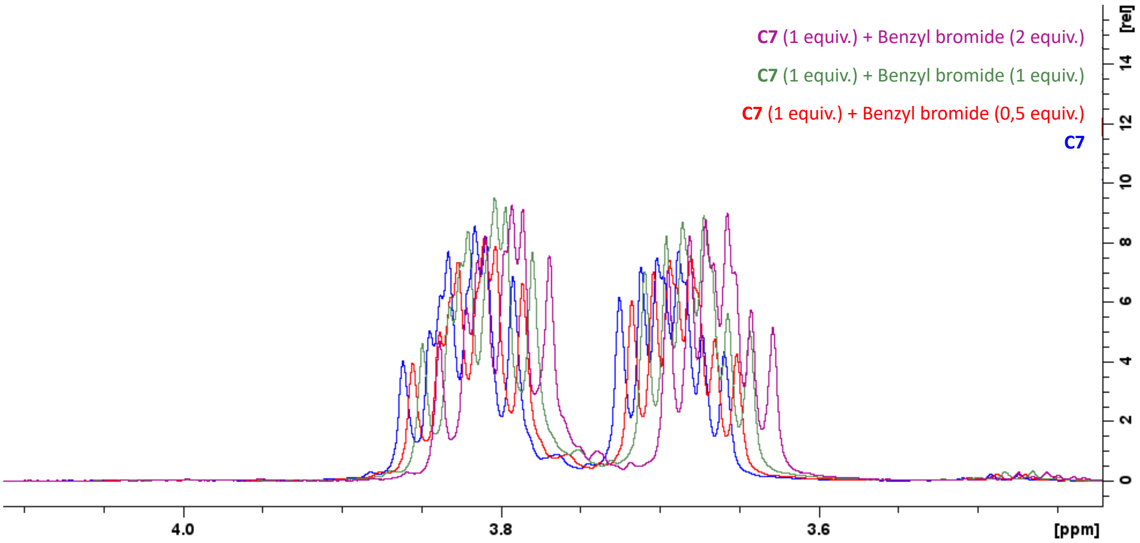

The signal shift of hydrogen H_b:

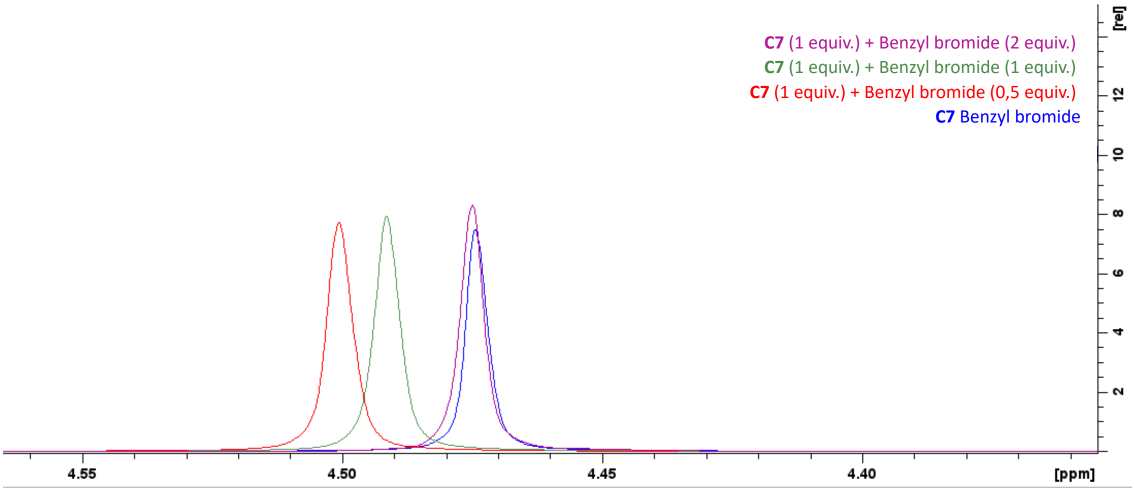

Optimization of the reaction conditions for the synthesis of thiocyanates without a catalyst:

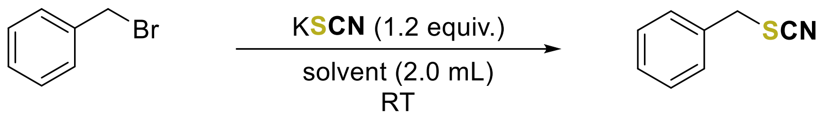

Entry	Solvent	Time	Isolated Yield (%)
1	Dimethyl carbonate	10 min	8
2	Anisole	10 min	3
3	Ethylene glycol	10 min	2
4	1-Butanol	10 min	6
5	Ethyl acetate	10 min	1
6	EtOH[a]	10 min	26
7	EtOH[a]	4 h	83
8	EtOH[a]	2 h	74

[a] 95% EtOH grade (used as received).

Experimental procedure: a test tube was charged with the corresponding solvent (2.0 mL) and benzyl bromide (85.5 mg, 0.5 mmol). The mixture was stirred until became homogeneous and then KSCN (58.3 mg, 0.6 mmol) was added. The reaction mixture was stirred at 25±2 °C (water bath) at a constant rate of 360 rpm during the period indicated on the table above. Afterwards, the mixture was extracted with AcOEt (3 x 10.0 mL) and the combined organic phases washed with water (3 x 10.0 mL), brine (3 x 10.0 mL), dried over $MgSO_4$ and evaporated under reduced pressure. Purification was performed by silica gel chromatography column with a mixture of hexanes/AcOEt = 9/1.

Substrate scope for the preparation of thio- and selenocyanates in uncatalyzed using hydrated EtOH as solvent.

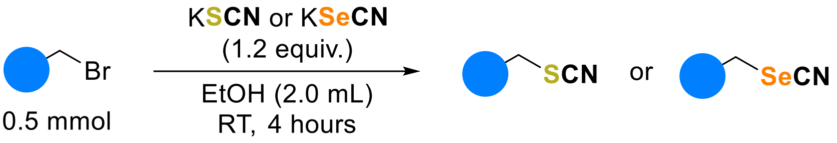

Entry	Product	Yield (%)[a]	Product	Yield (%)[a]
1	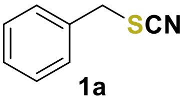 **1a**	83	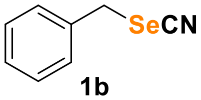 **1b**	75
2	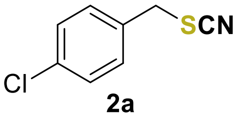 **2a**	79		
3	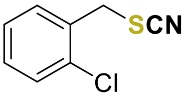 **3a**	65		
4	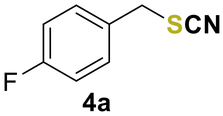 **4a**	35		
5	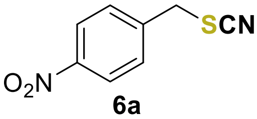 **6a**	81	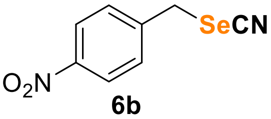 **6b**	86
6	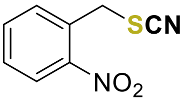 **7a**	49		
7	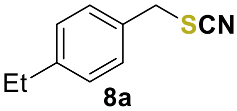 **8a**	81		
8	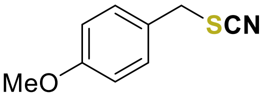 **9a**	47	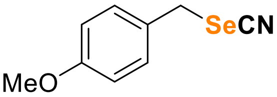 **9b**	47
9	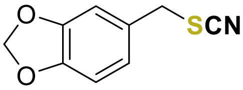 **10a**	78	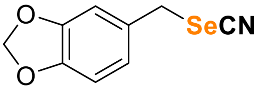 **10b**	69
10	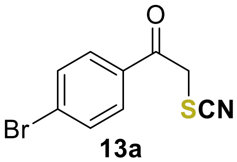 **13a**	82	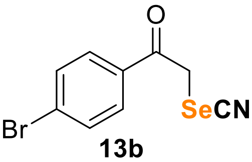 **13b**	78

[a] Yields for pure and isolated products.

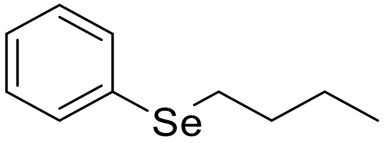

C1

^{1}H NMR ($CDCl_3$, 400 MHz)

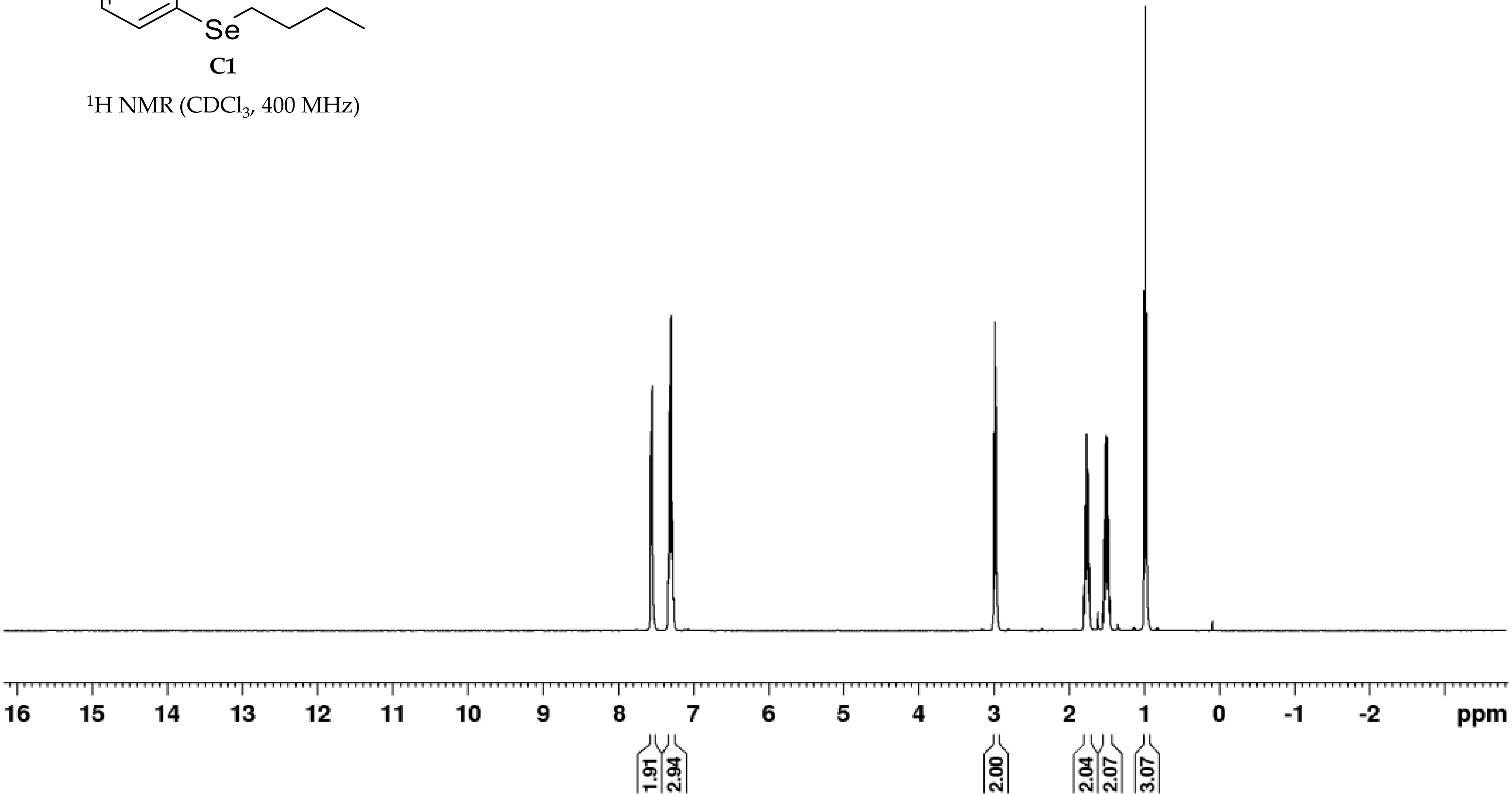

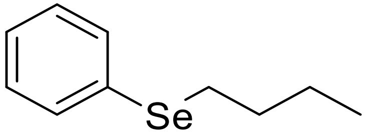

C1

^{13}C NMR ($CDCl_3$, 100 MHz)

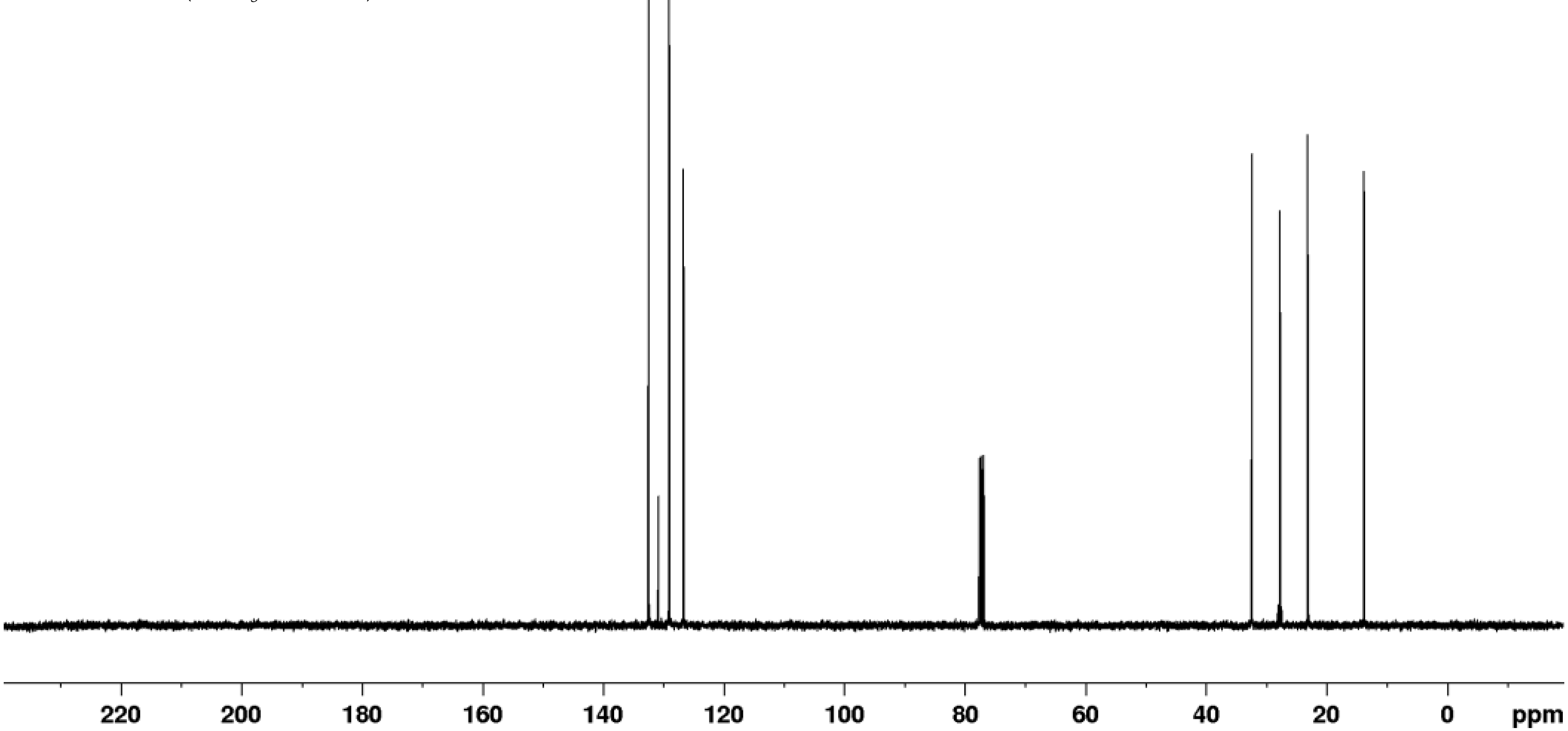

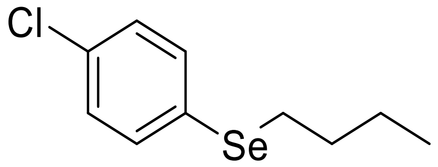

C2

^{1}H NMR ($CDCl_3$, 400 MHz)

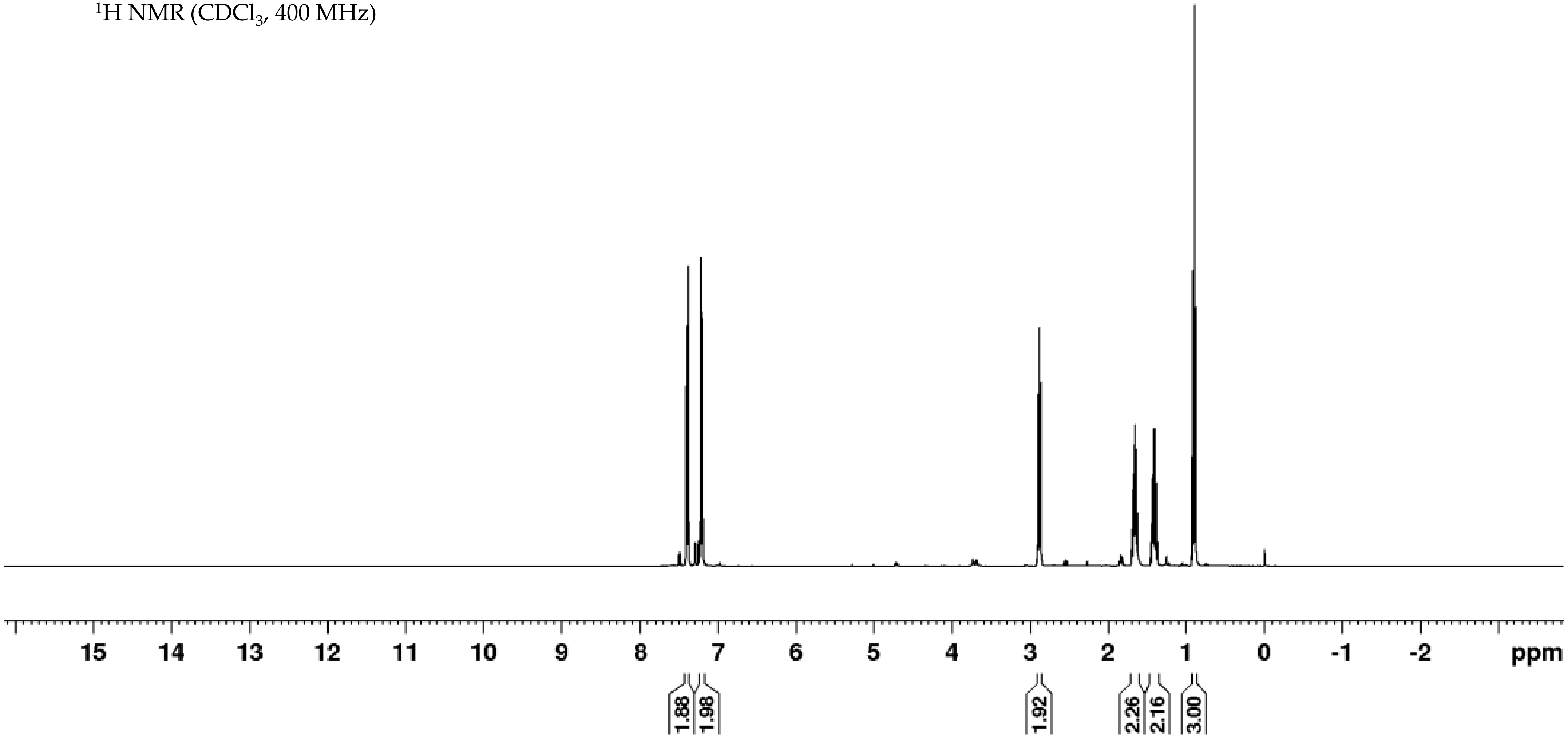

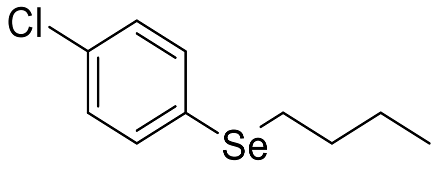

C2

^{13}C NMR ($CDCl_3$, 100 MHz)

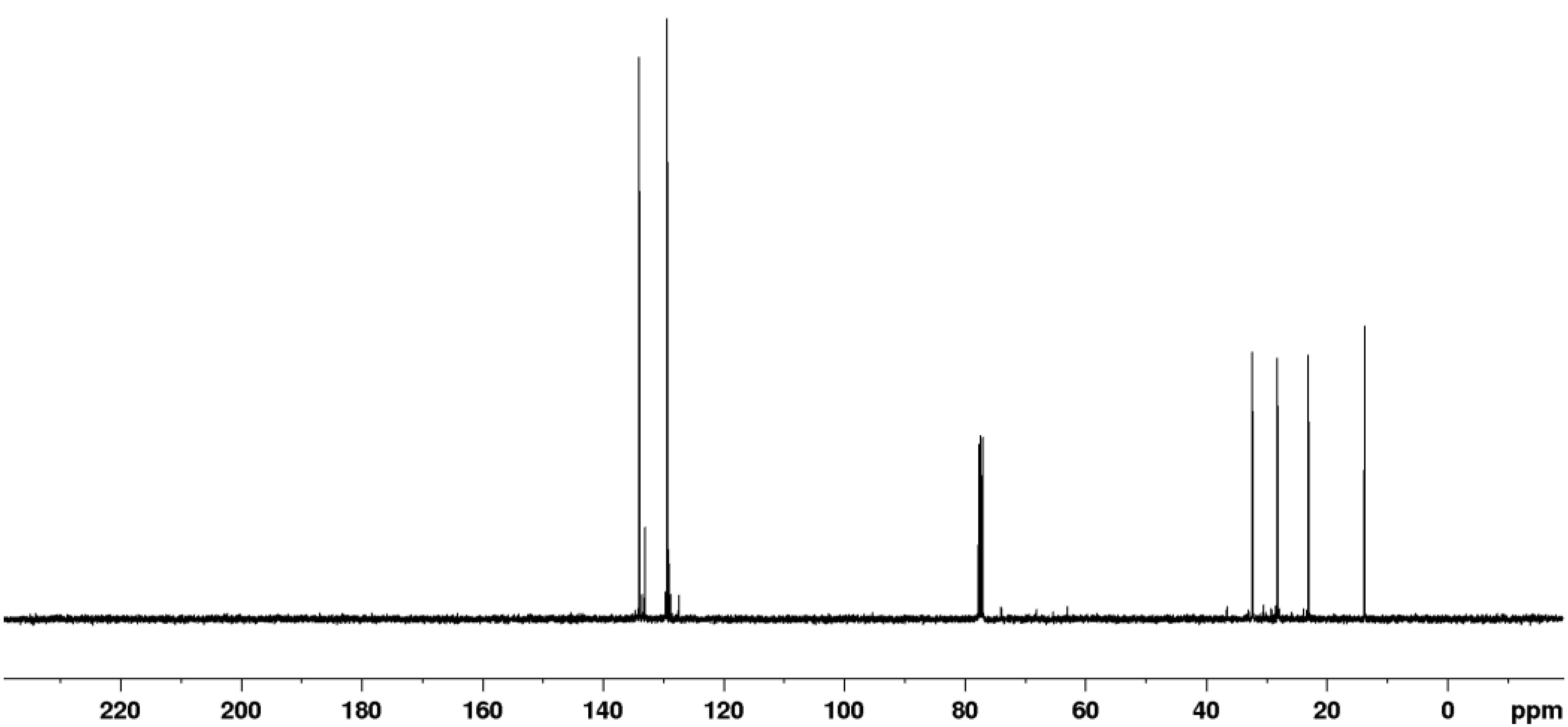

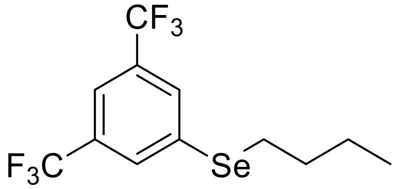

C3

^{1}H NMR ($CDCl_3$, 400 MHz)

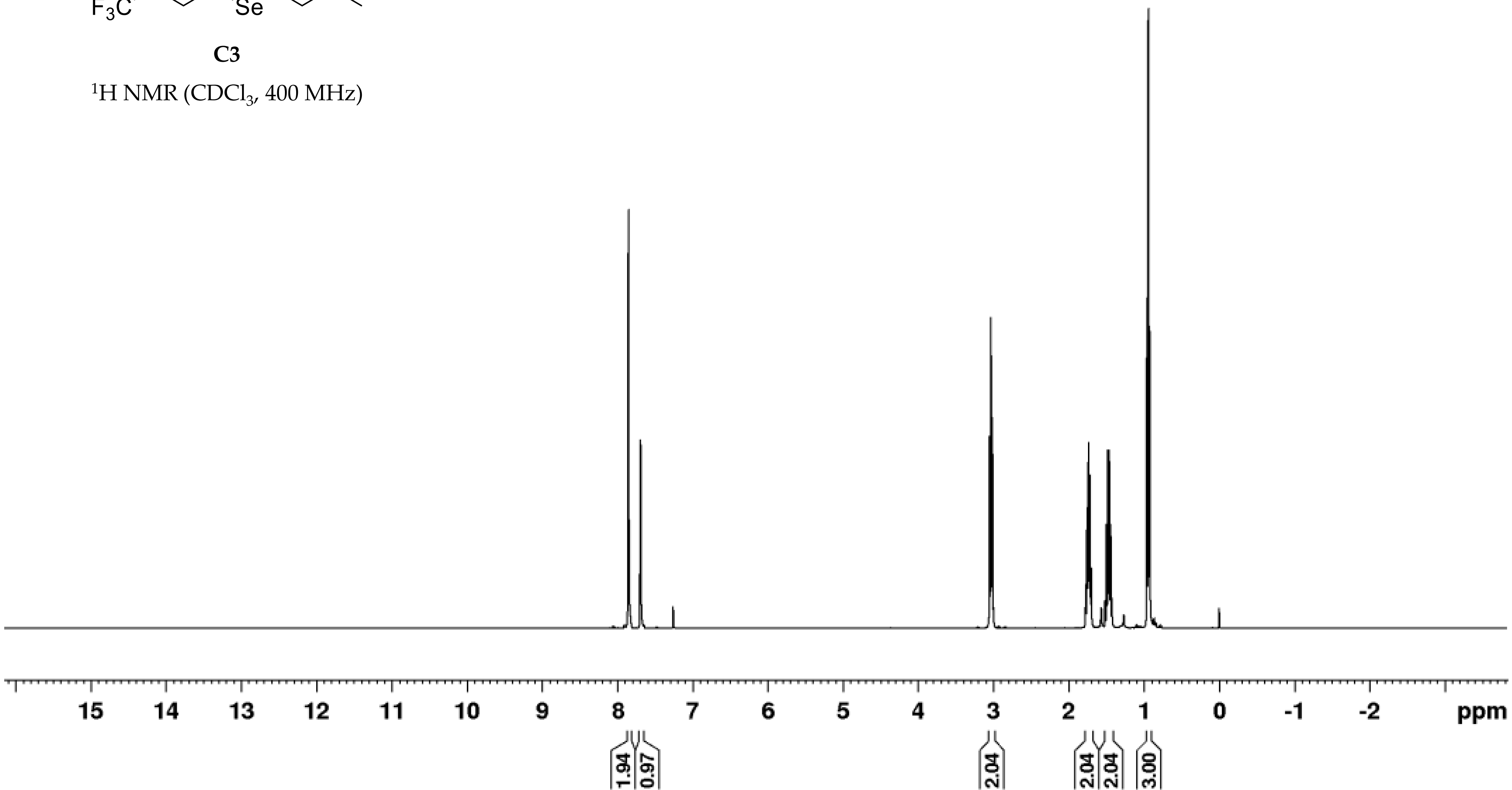

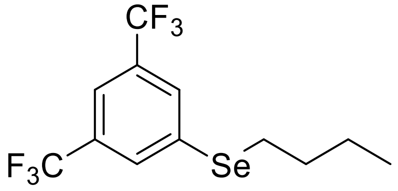

C3

^{13}C NMR ($CDCl_3$, 100 MHz)

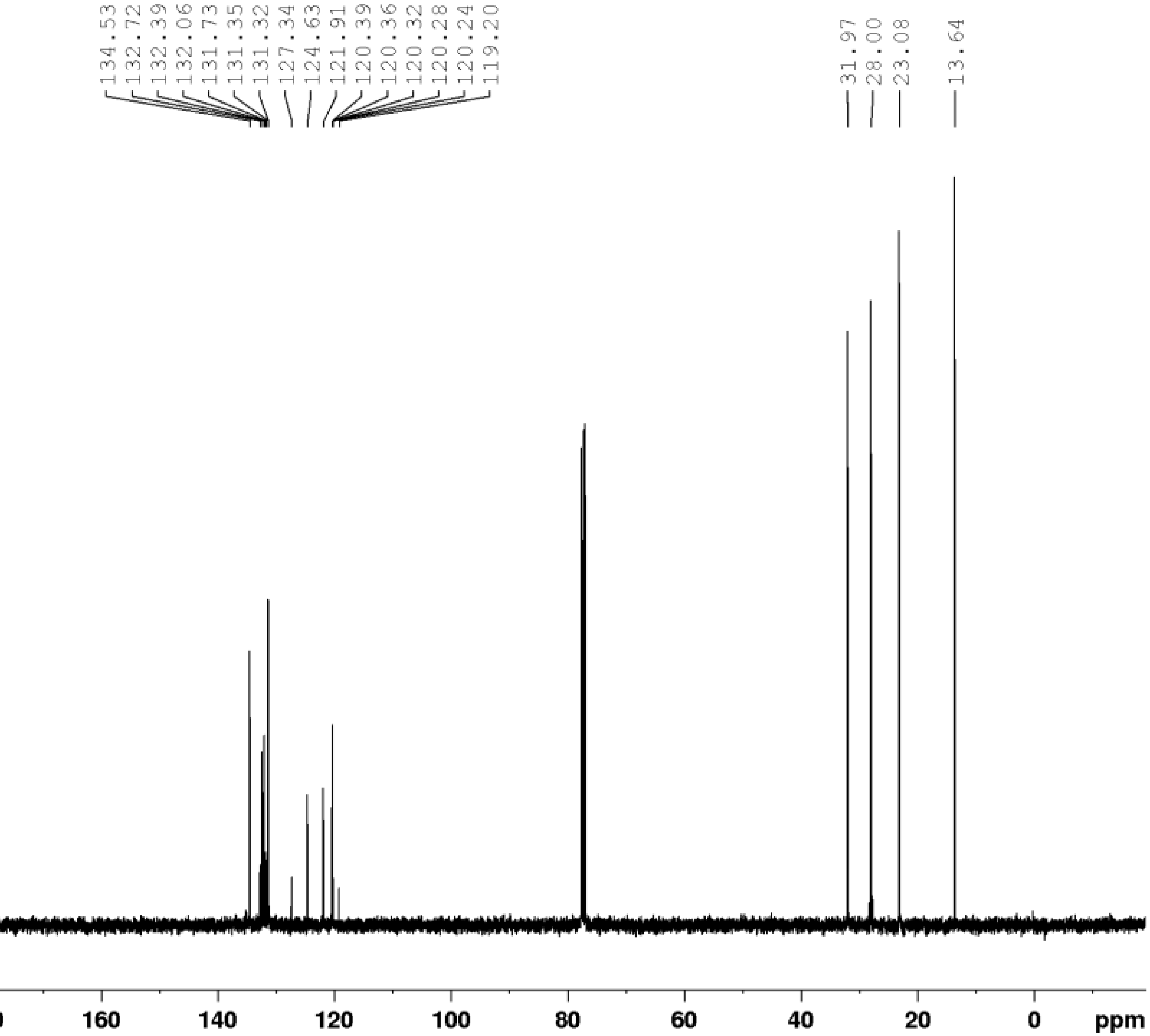

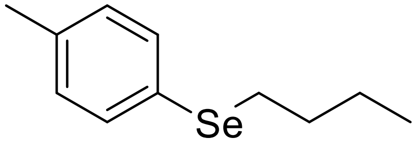

C4

^{1}H NMR ($CDCl_3$, 400 MHz)

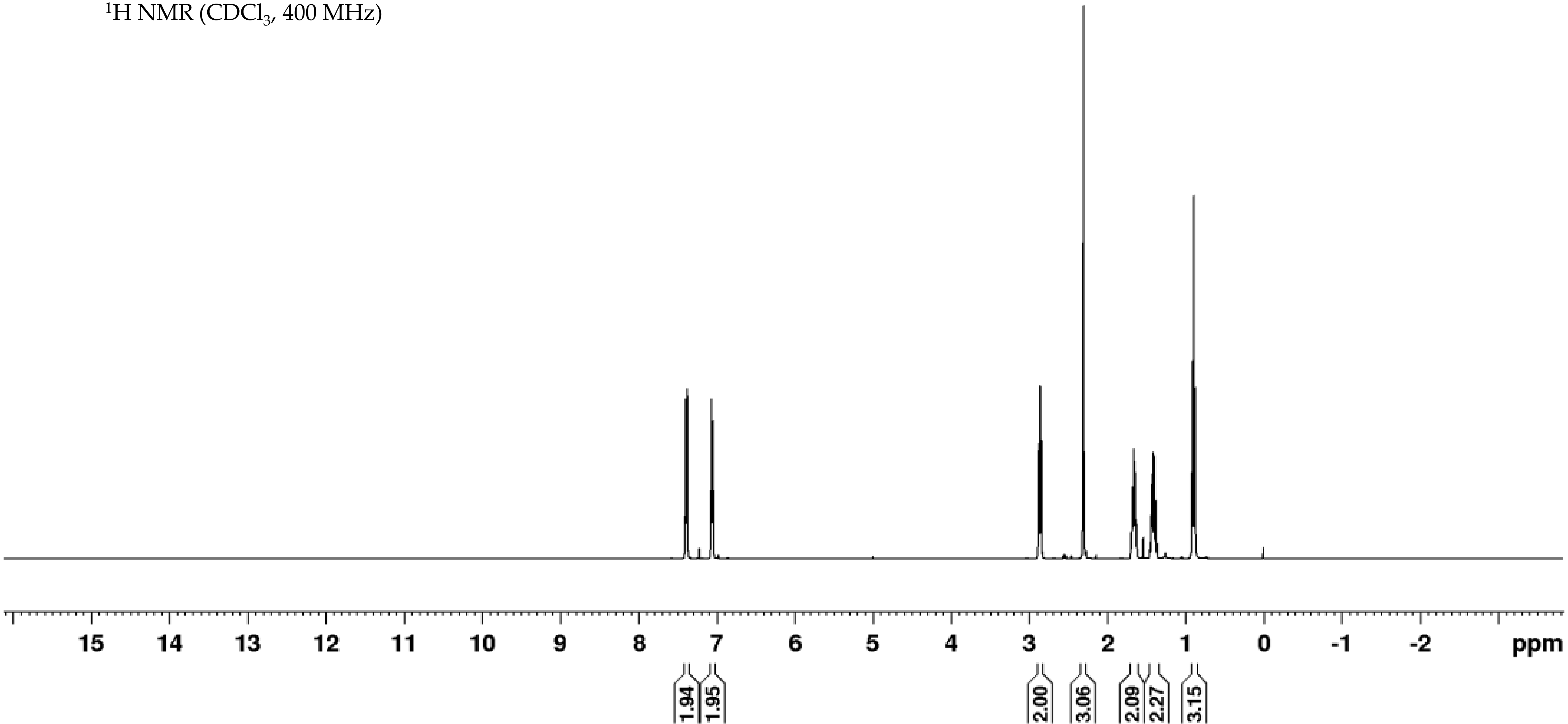

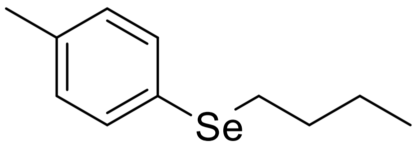

C4

^{13}C NMR ($CDCl_3$, 100 MHz)

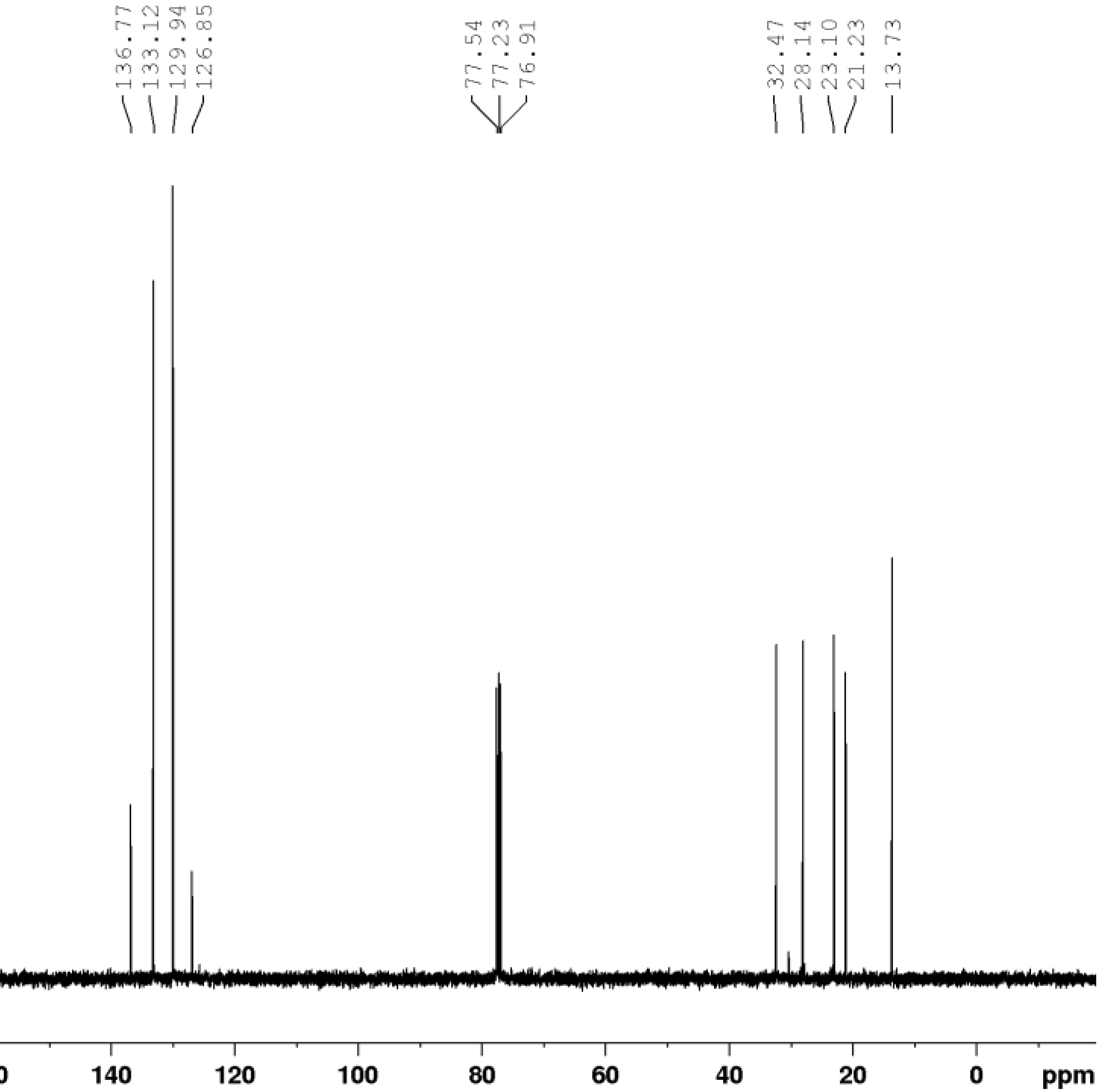

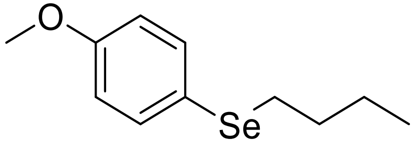

C5

^{1}H NMR ($CDCl_3$, 400 MHz)

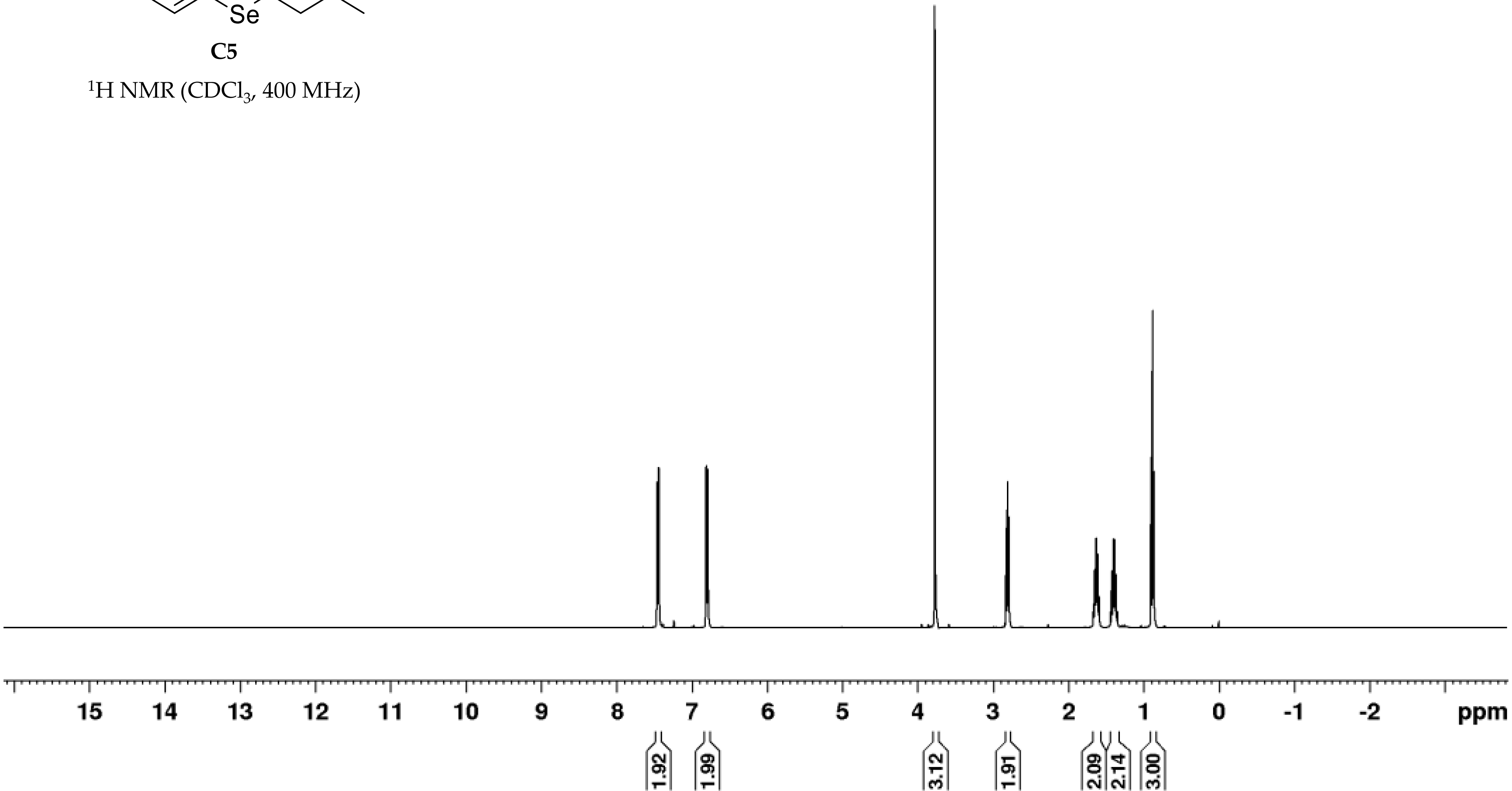

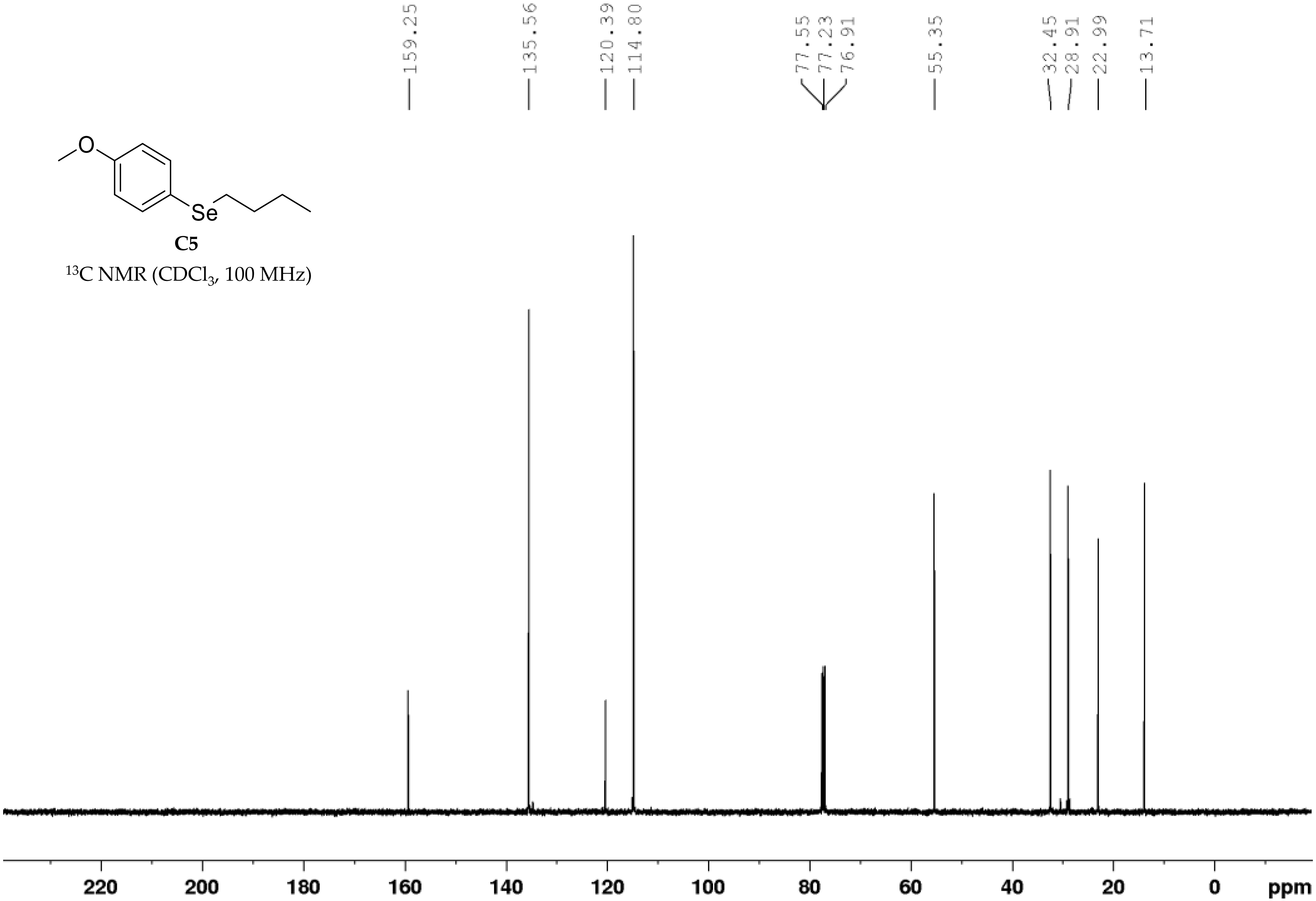
159.25
135.56
120.39
114.80
77.55
77.23
76.91
55.35
32.45
28.91
22.99
13.71
O
Se
C5
13C NMR (CDCl3, 100 MHz)
220
200
180
160
140
120
100
80
60
40
20
0
ppm

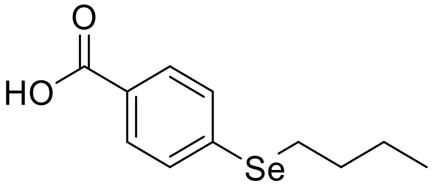

C6

^{1}H NMR ($CDCl_3$, 400 MHz)

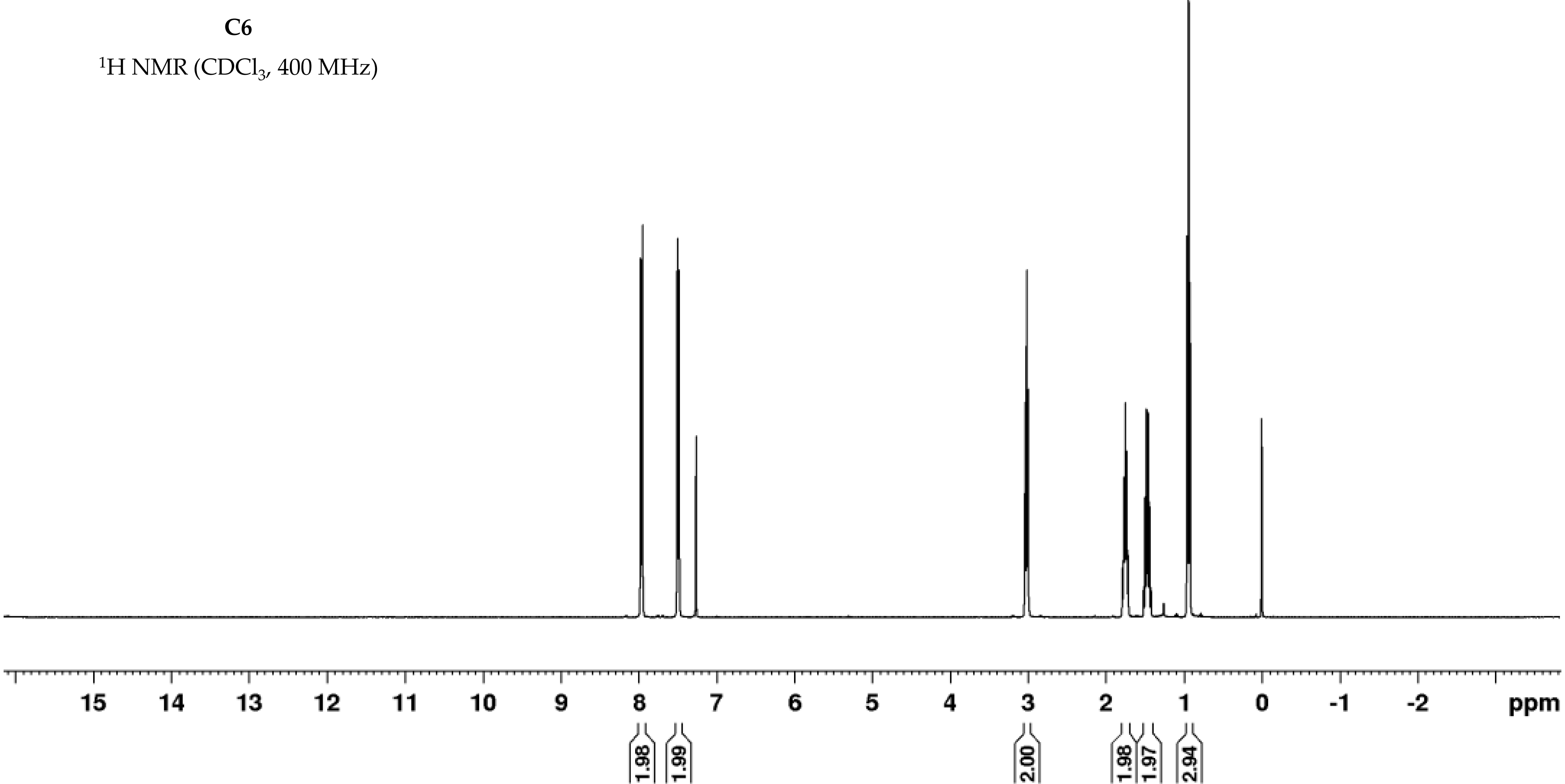

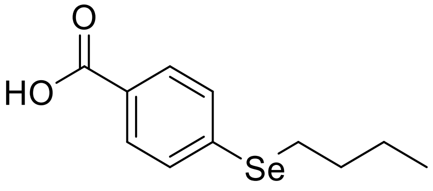

C6

^{13}C NMR ($CDCl_3$, 100 MHz)

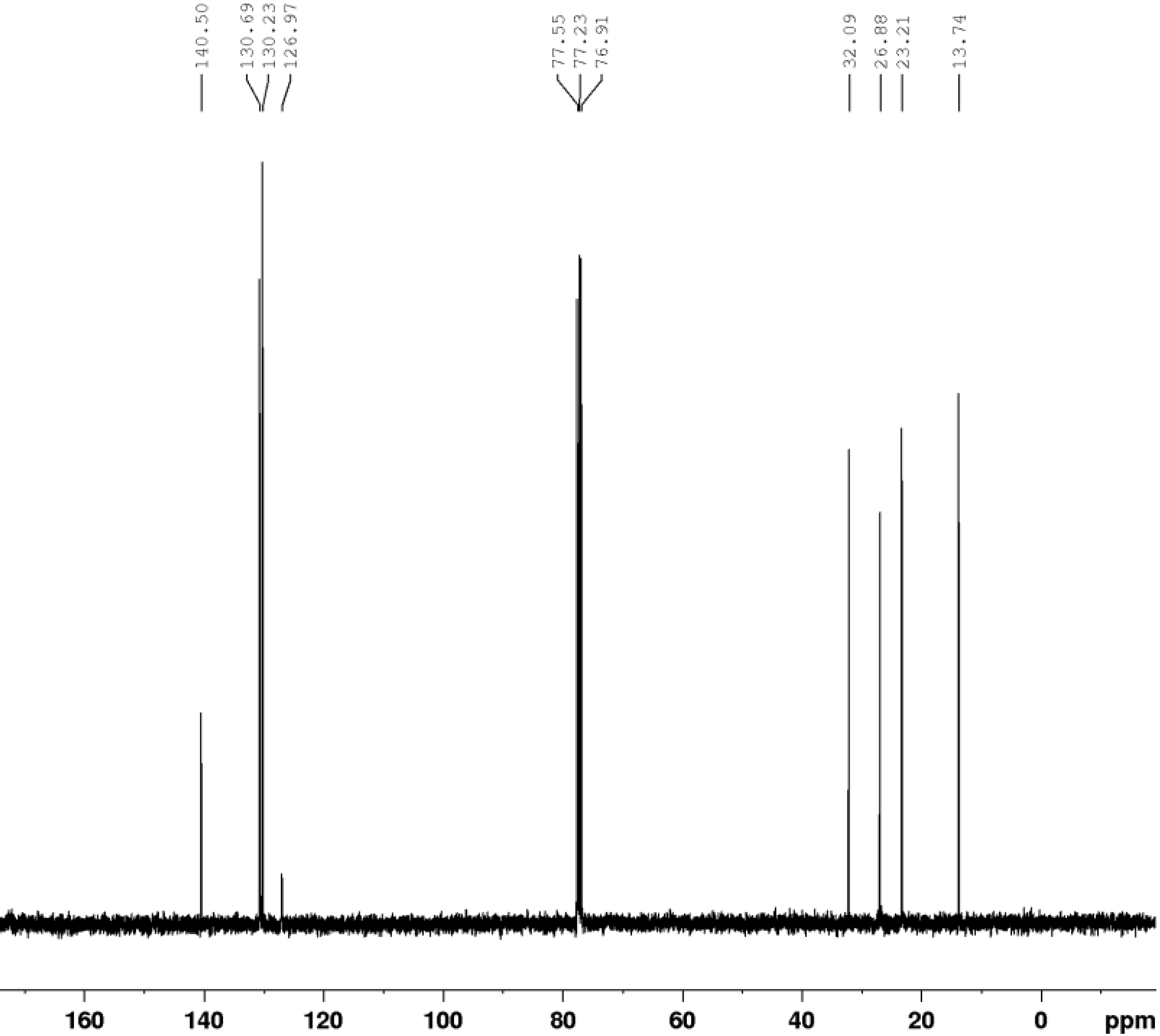

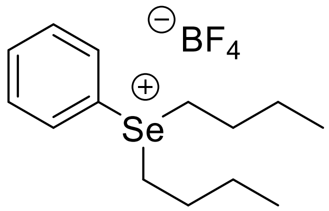

C7

^{1}H NMR ($CDCl_3$, 400 MHz)

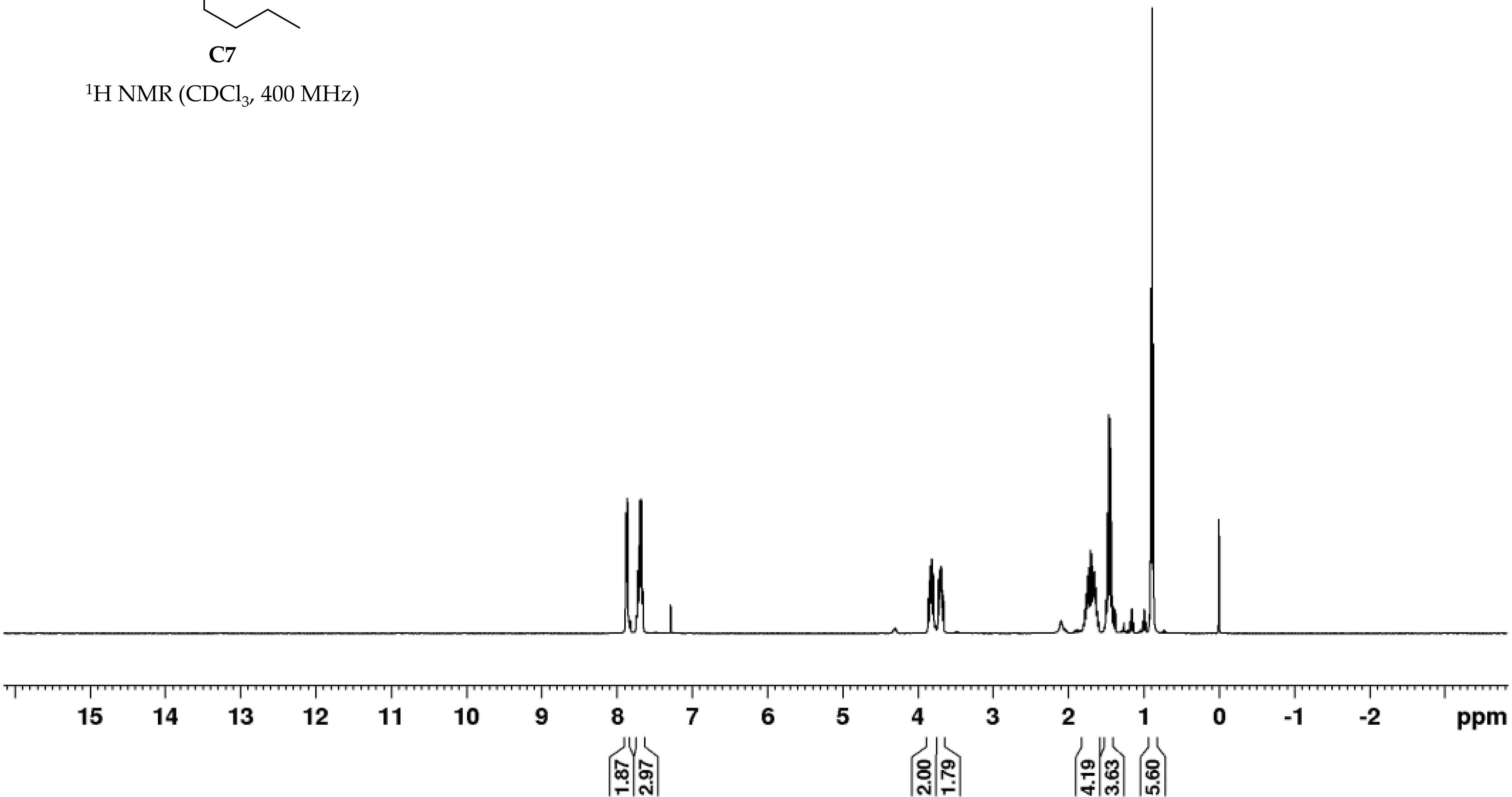

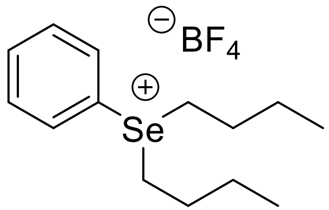

C7

^{13}C NMR ($CDCl_3$, 100 MHz)

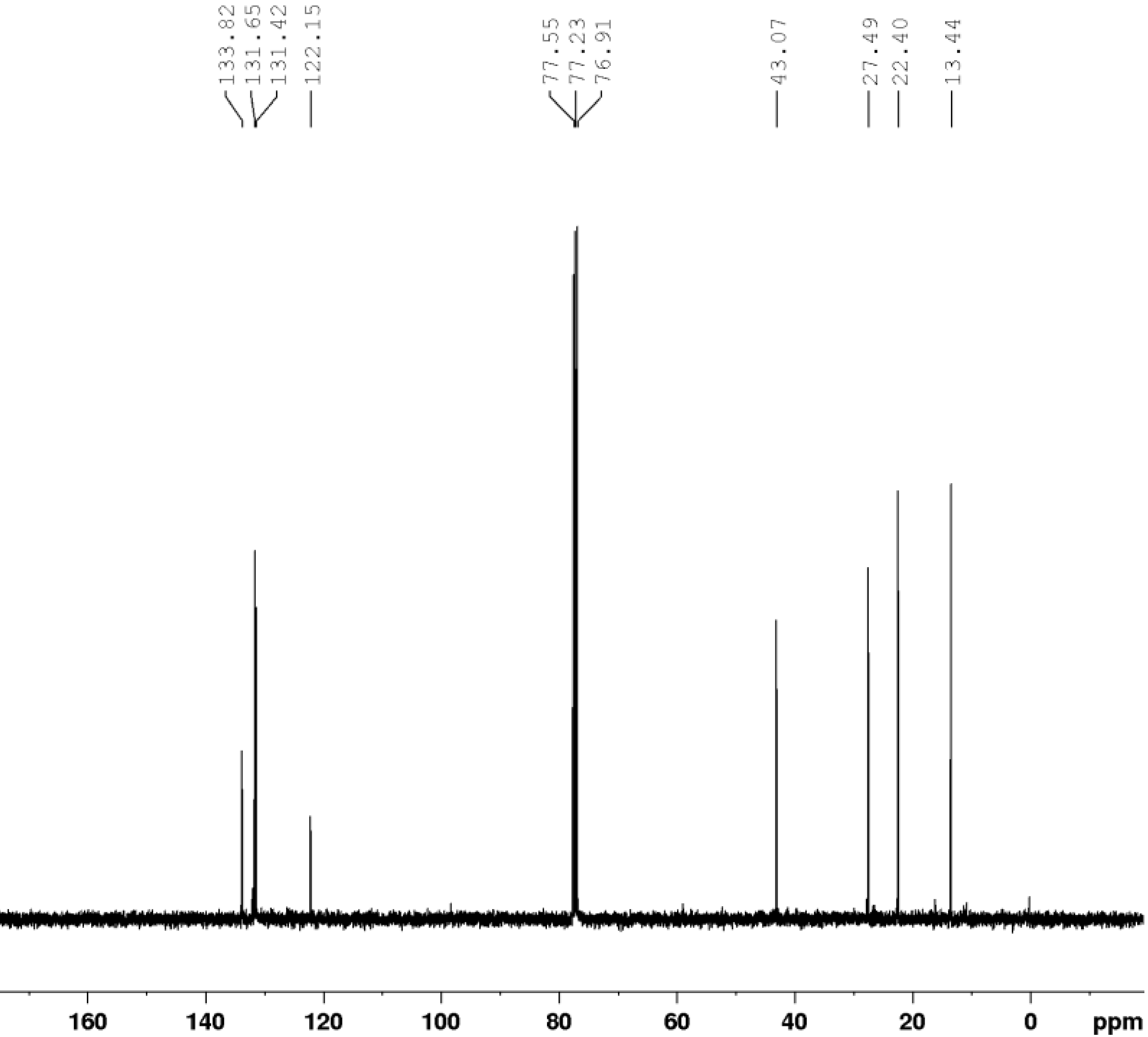

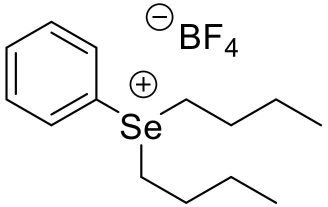

C7

^{77}Se NMR ($CDCl_3$, 76 MHz)

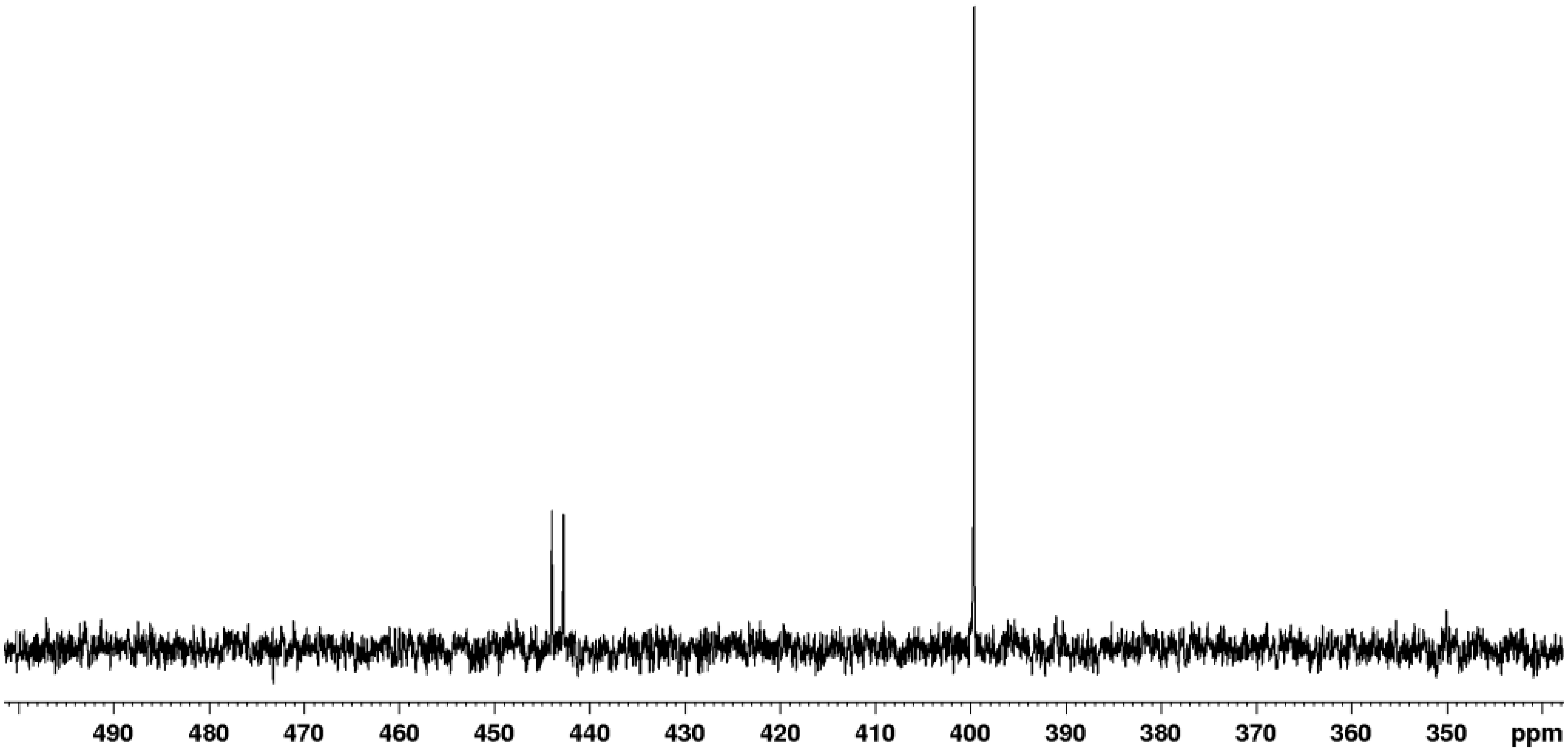

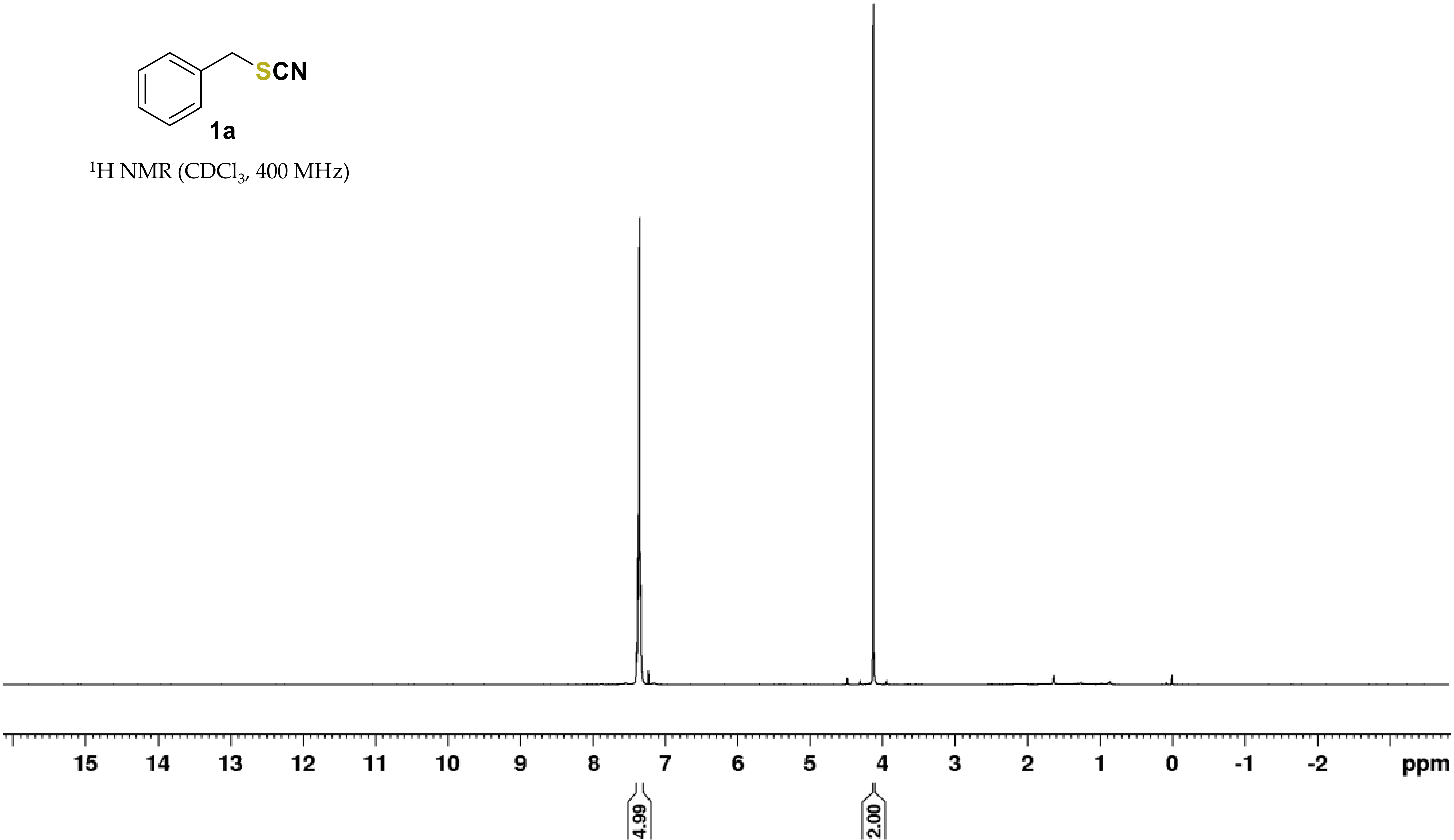

^{1}H NMR ($CDCl_3$, 400 MHz)

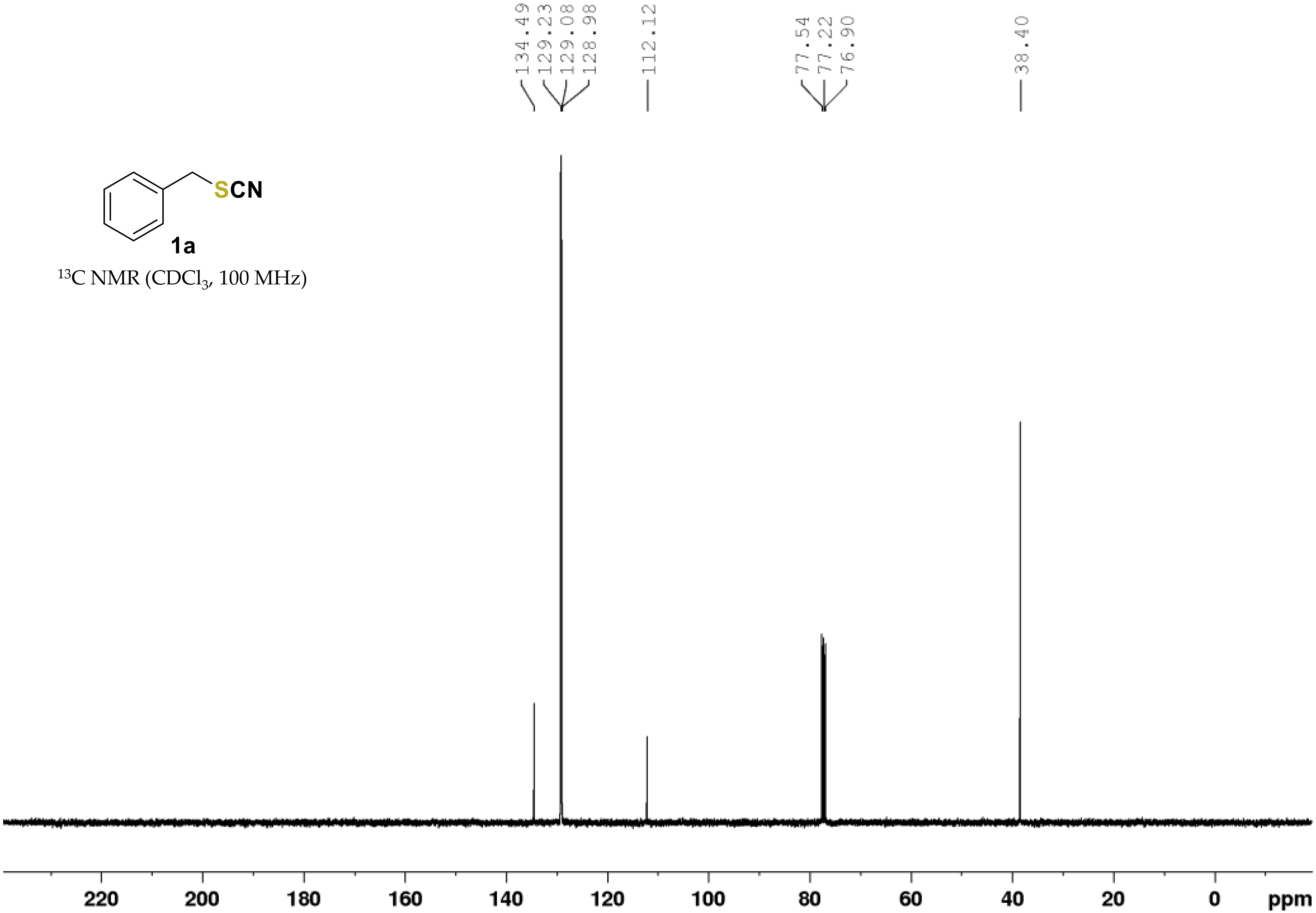

1a

^{13}C NMR ($CDCl_3$, 100 MHz)

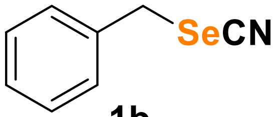

1b

^{1}H NMR ($CDCl_3$, 400 MHz)

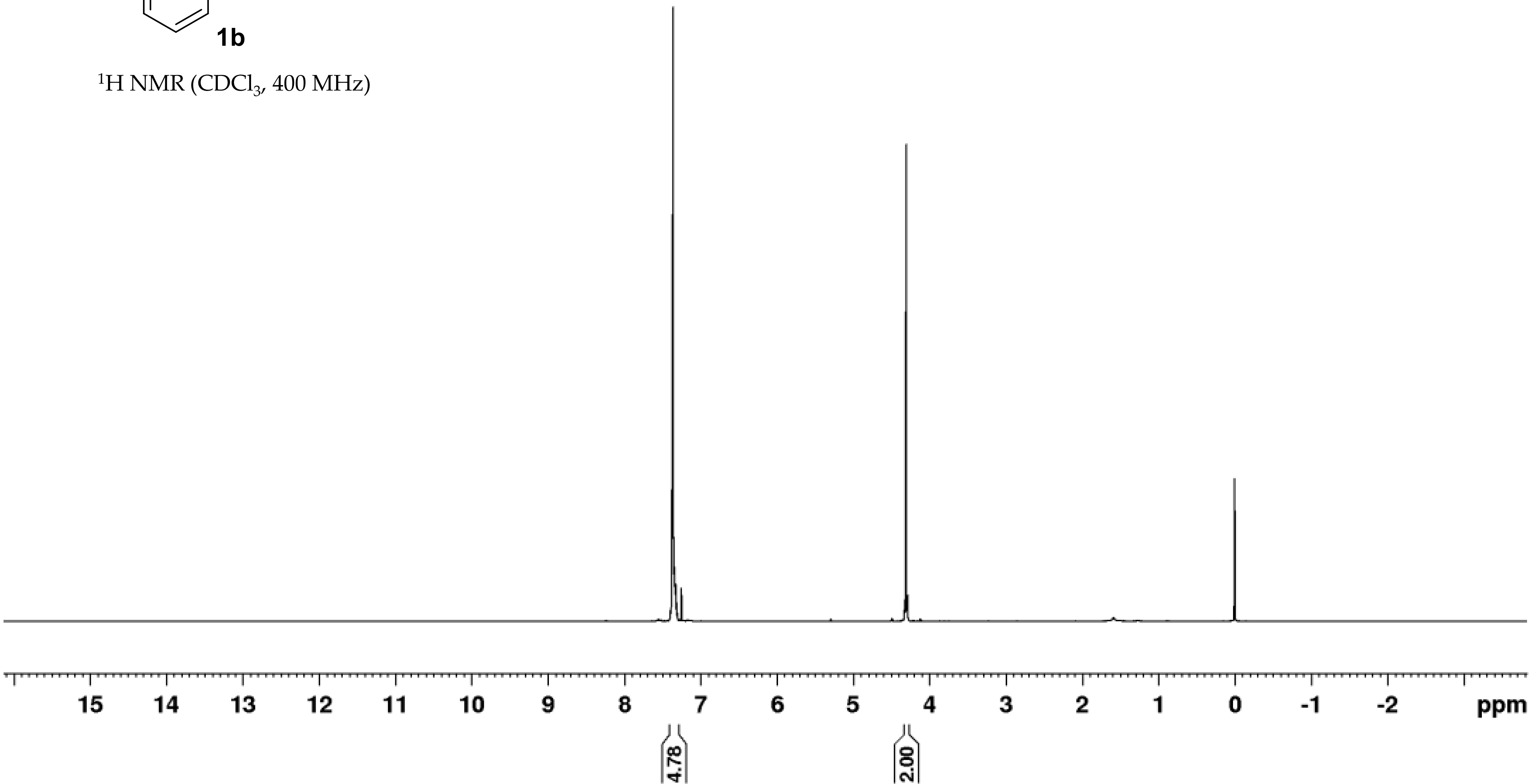

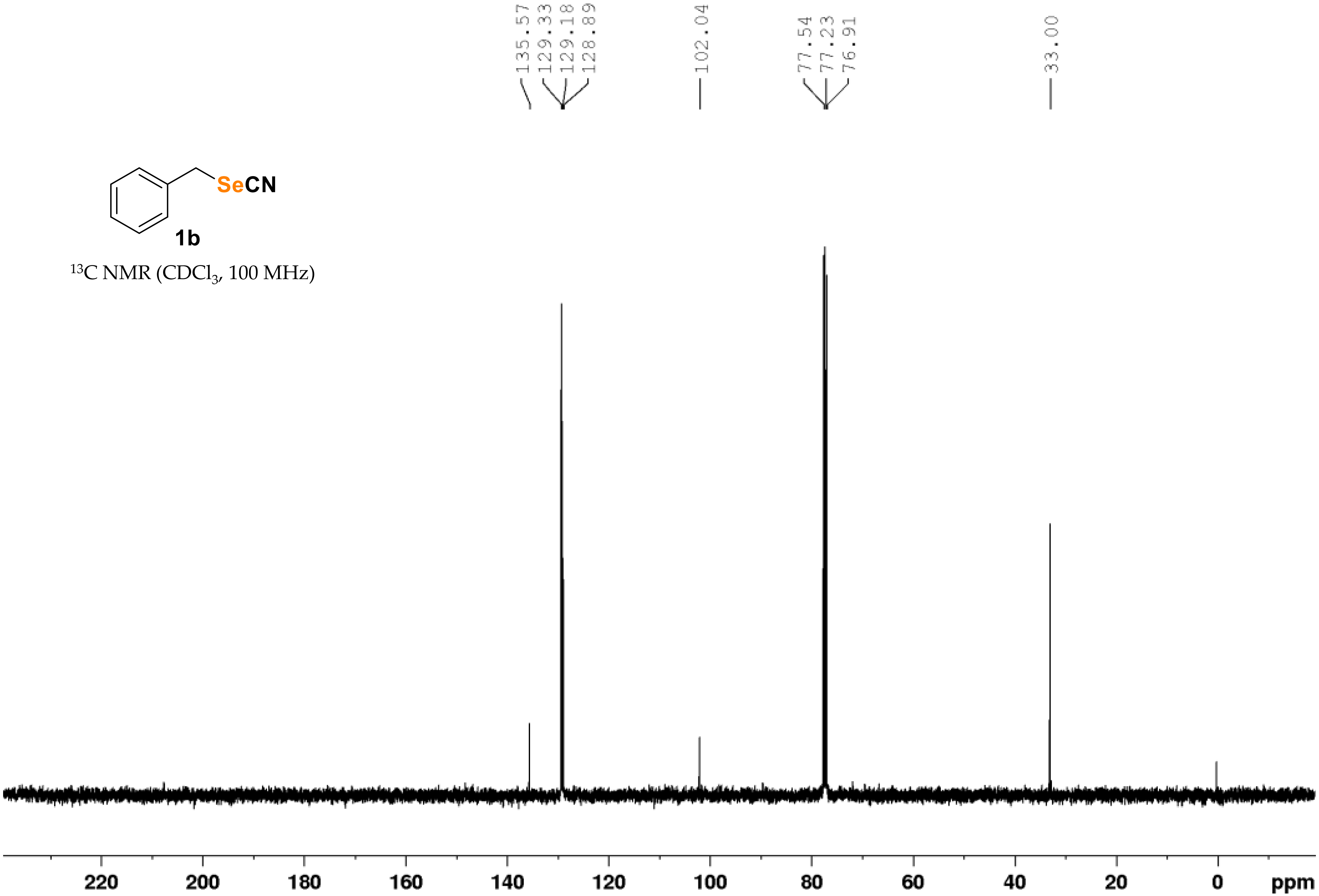

1b

^{13}C NMR ($CDCl_3$, 100 MHz)

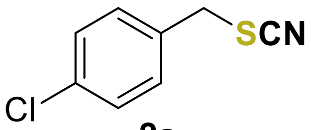

2a

^{1}H NMR ($CDCl_3$, 400 MHz)

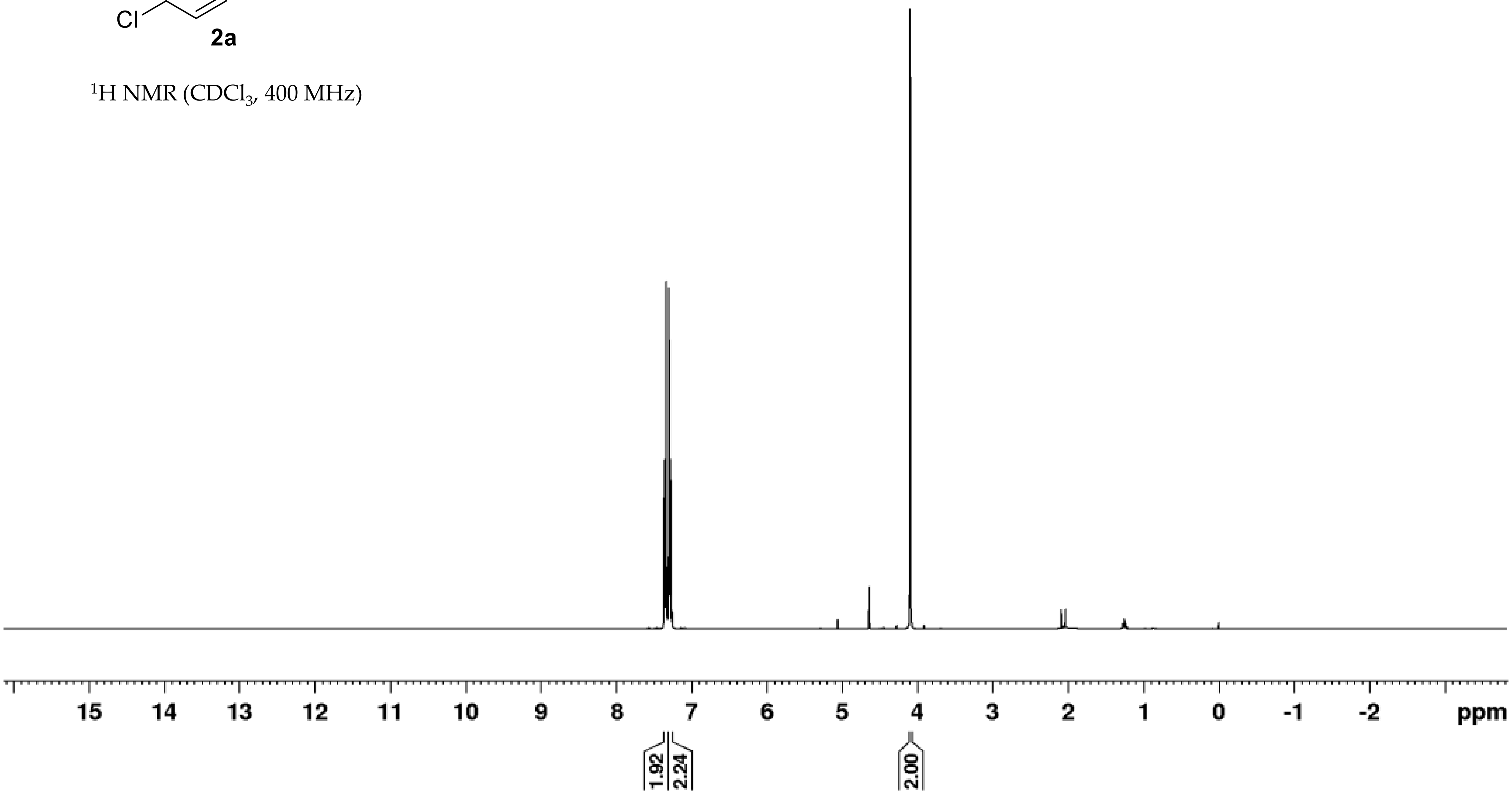

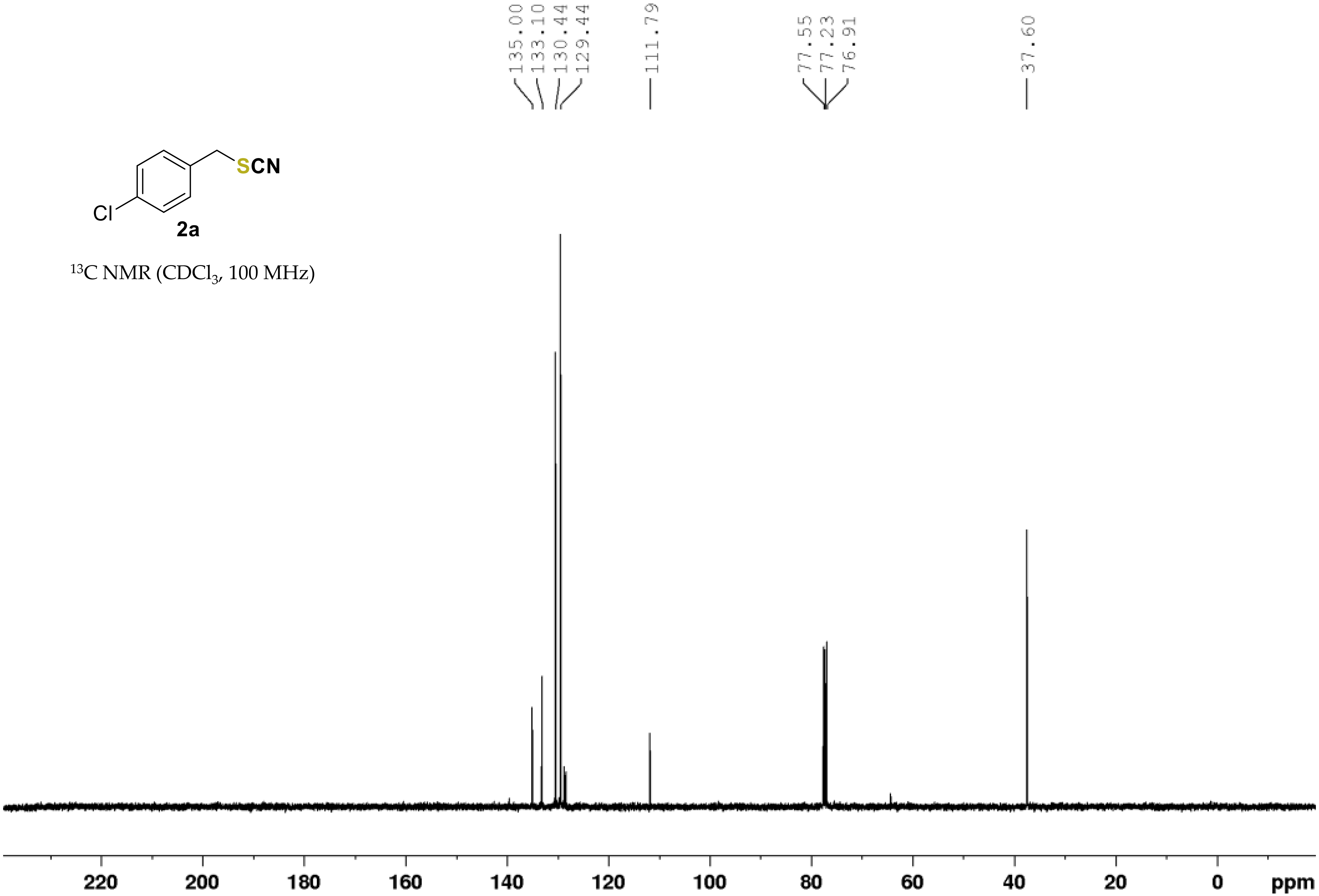
SCN
Cl
2a
13C NMR (CDCl3, 100 MHz)
135.00
133.10
130.44
129.44
111.79
77.55
77.23
76.91
37.60
220
200
180
160
140
120
100
80
60
40
20
0
ppm

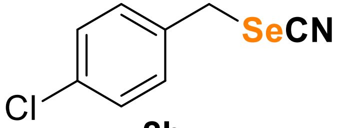

2b

^{1}H NMR ($CDCl_3$, 400 MHz)

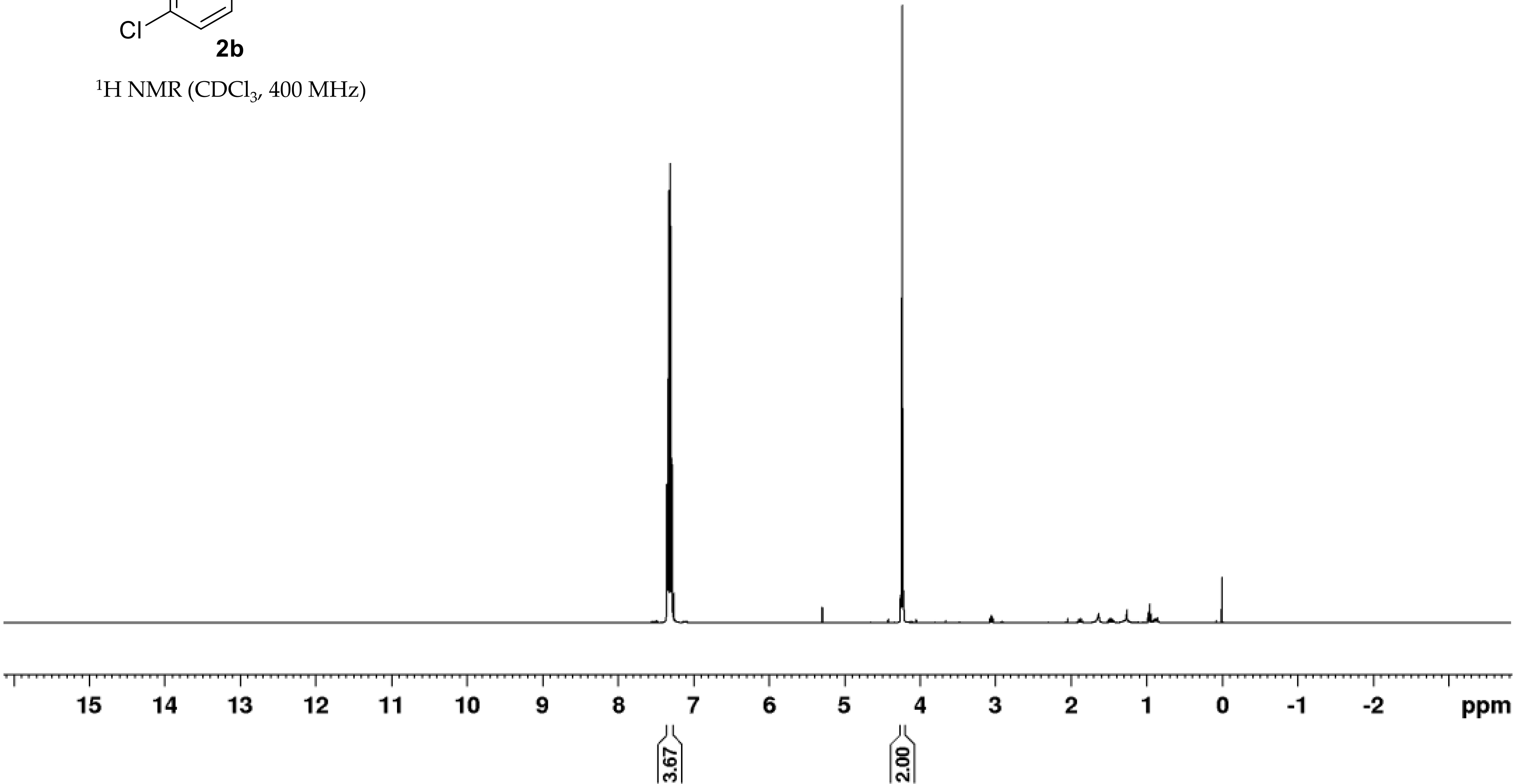

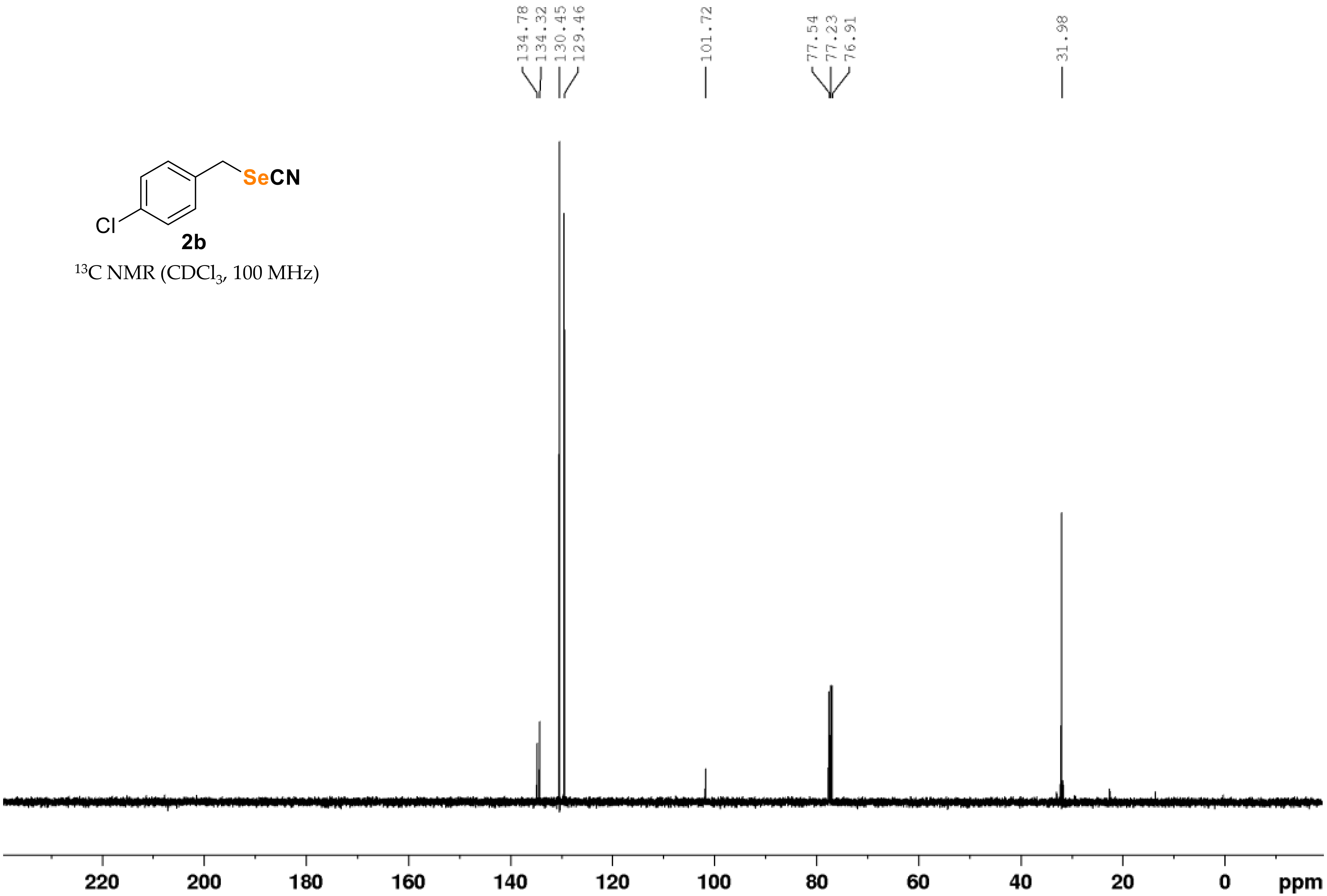
134.78
134.32
130.45
129.46
101.72
77.54
77.23
76.91
31.98
SeCN
Cl
2b
^{13}C NMR ($CDCl_3$, 100 MHz)
220
200
180
160
140
120
100
80
60
40
20
0
ppm

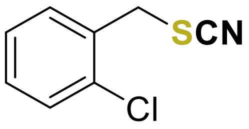

3a

^{1}H NMR (CDCl$_3$, 400 MHz)

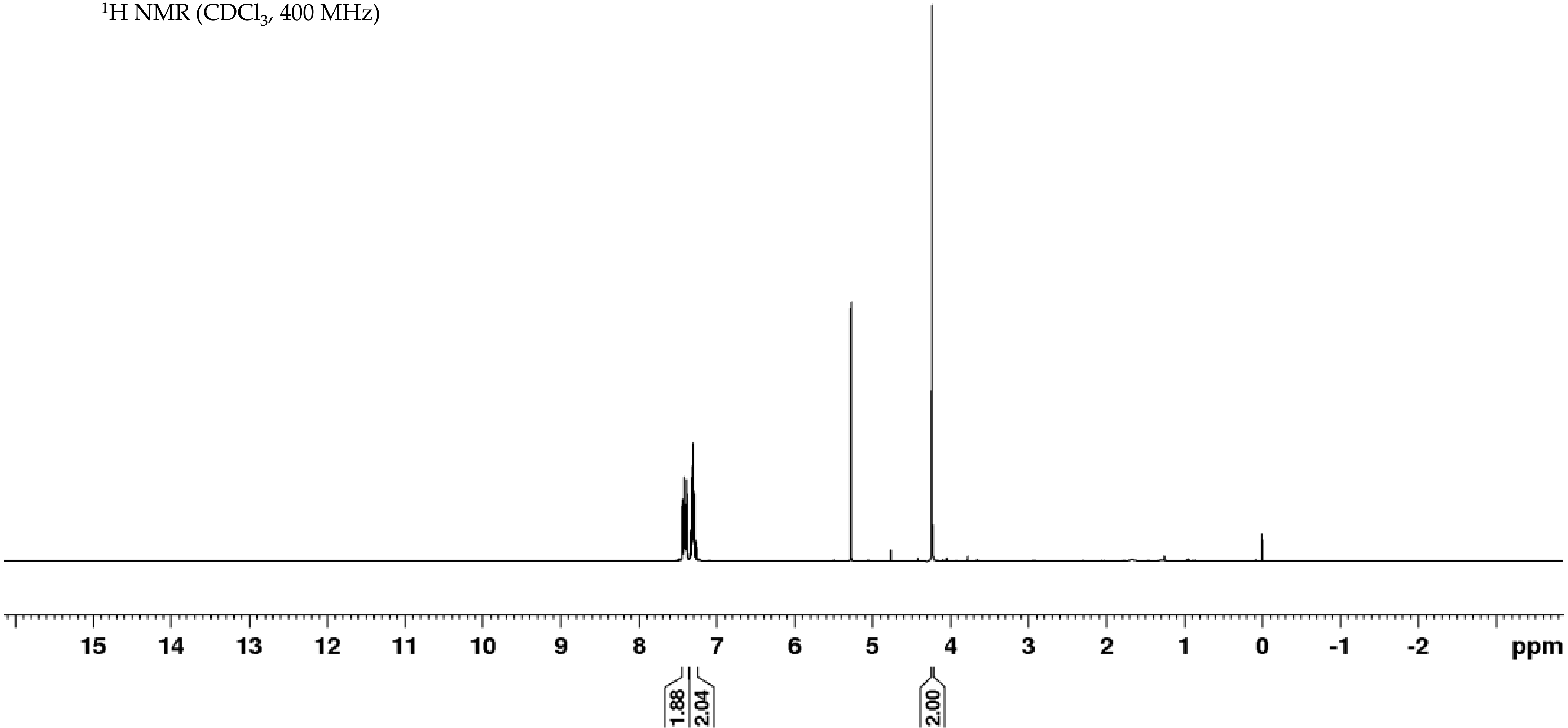

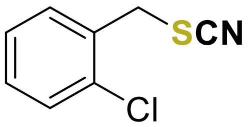

3a

^{13}C NMR ($CDCl_3$, 100 MHz)

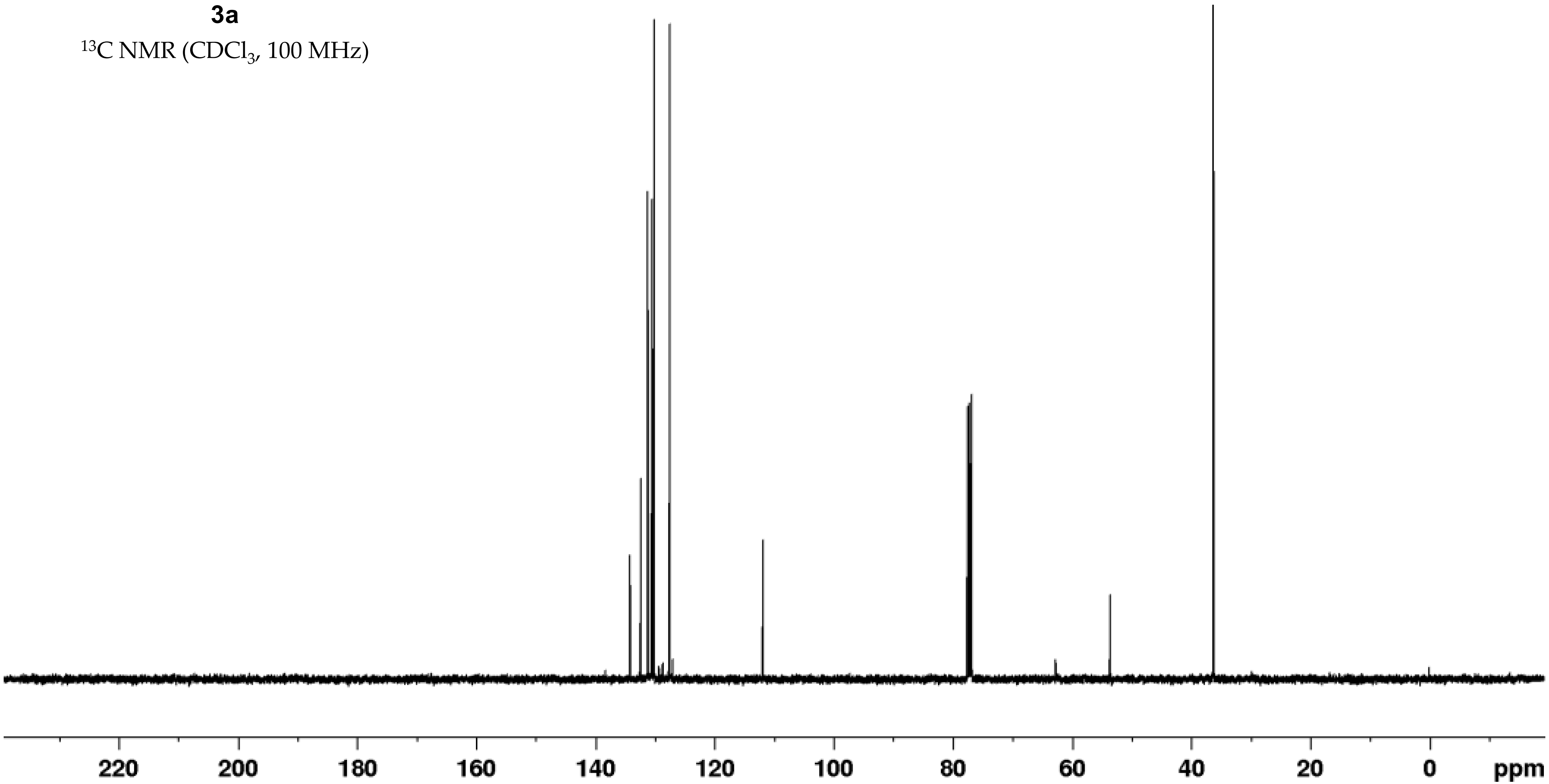

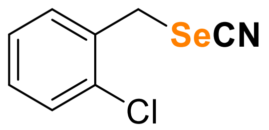

3b

^{1}H NMR ($CDCl_3$, 400 MHz)

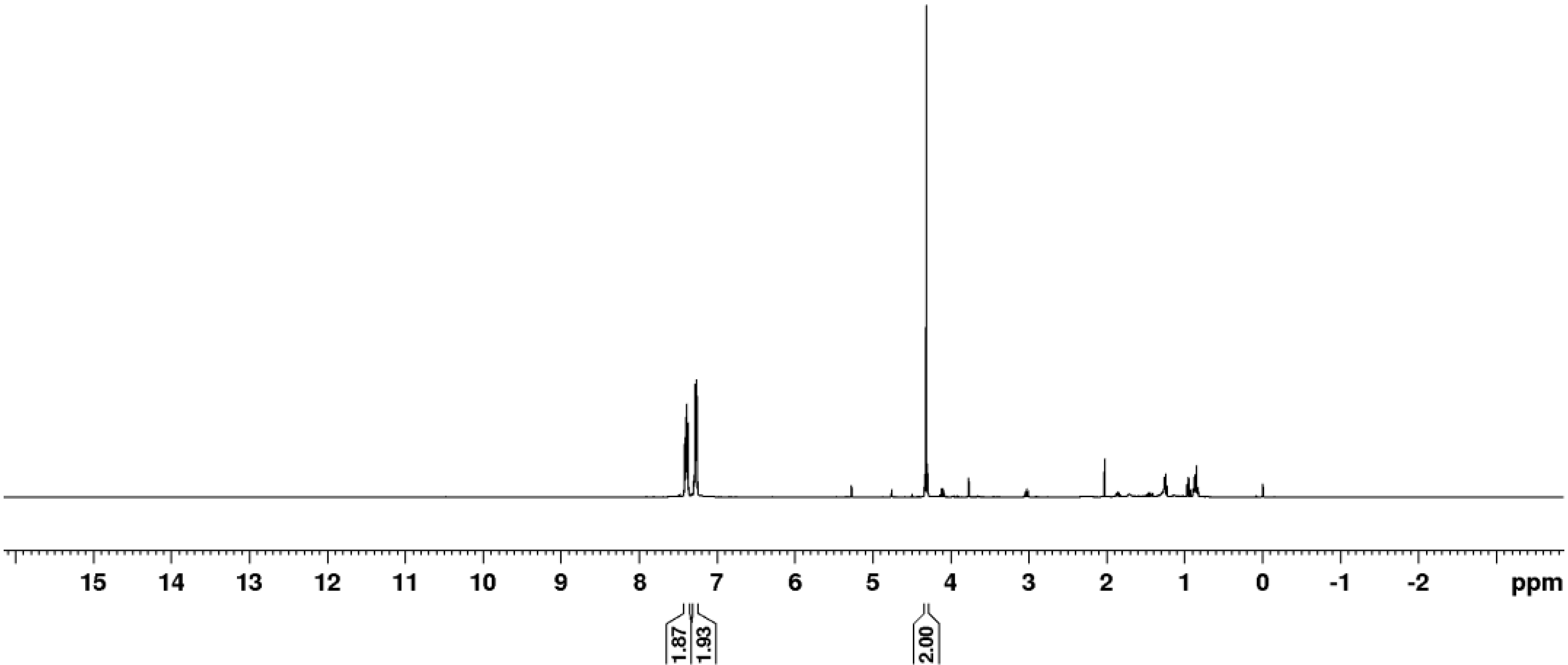

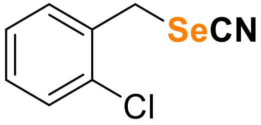

3b

^{13}C NMR ($CDCl_3$, 100 MHz)

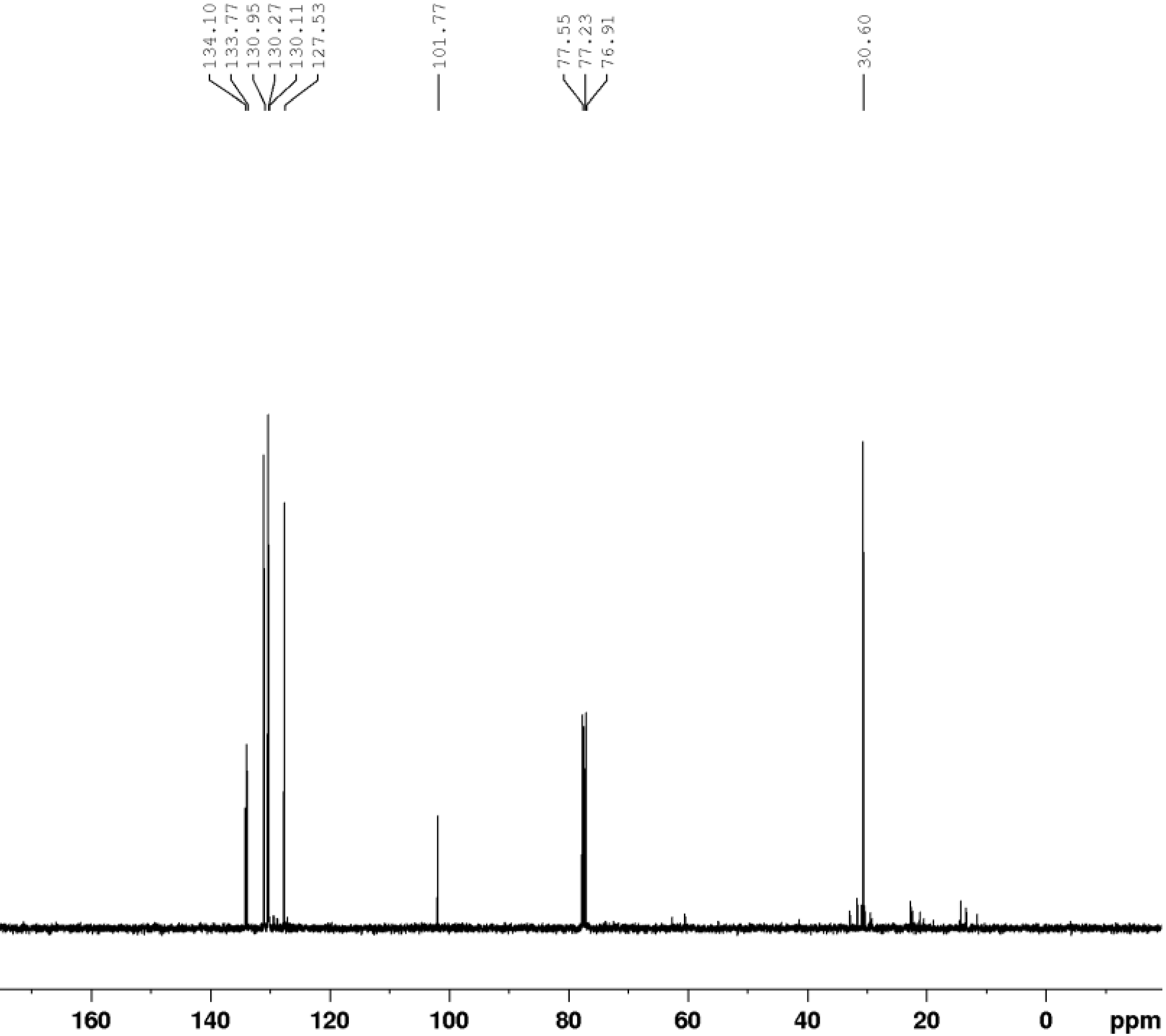

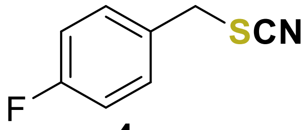

4a

^{1}H NMR ($CDCl_3$, 400 MHz)

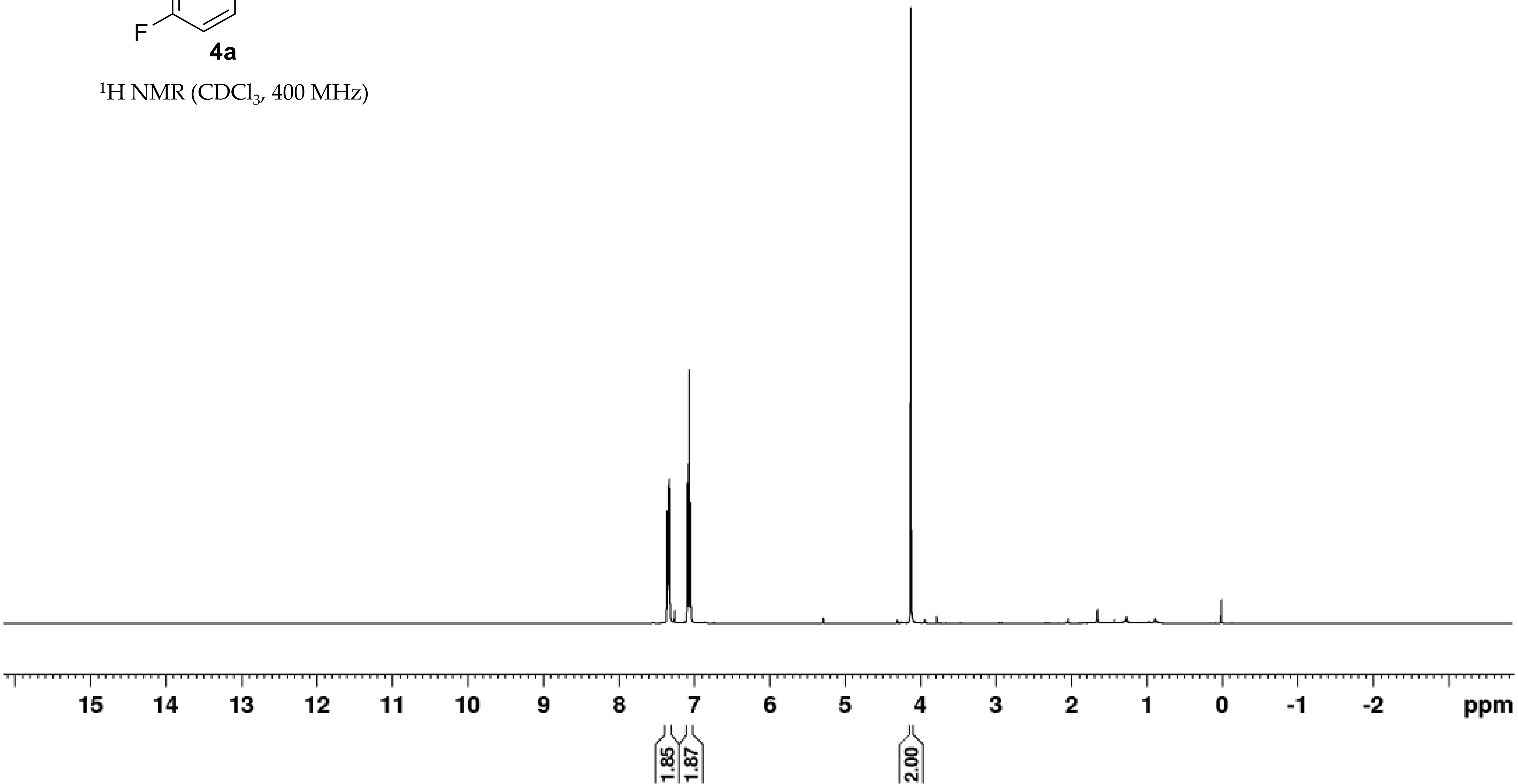

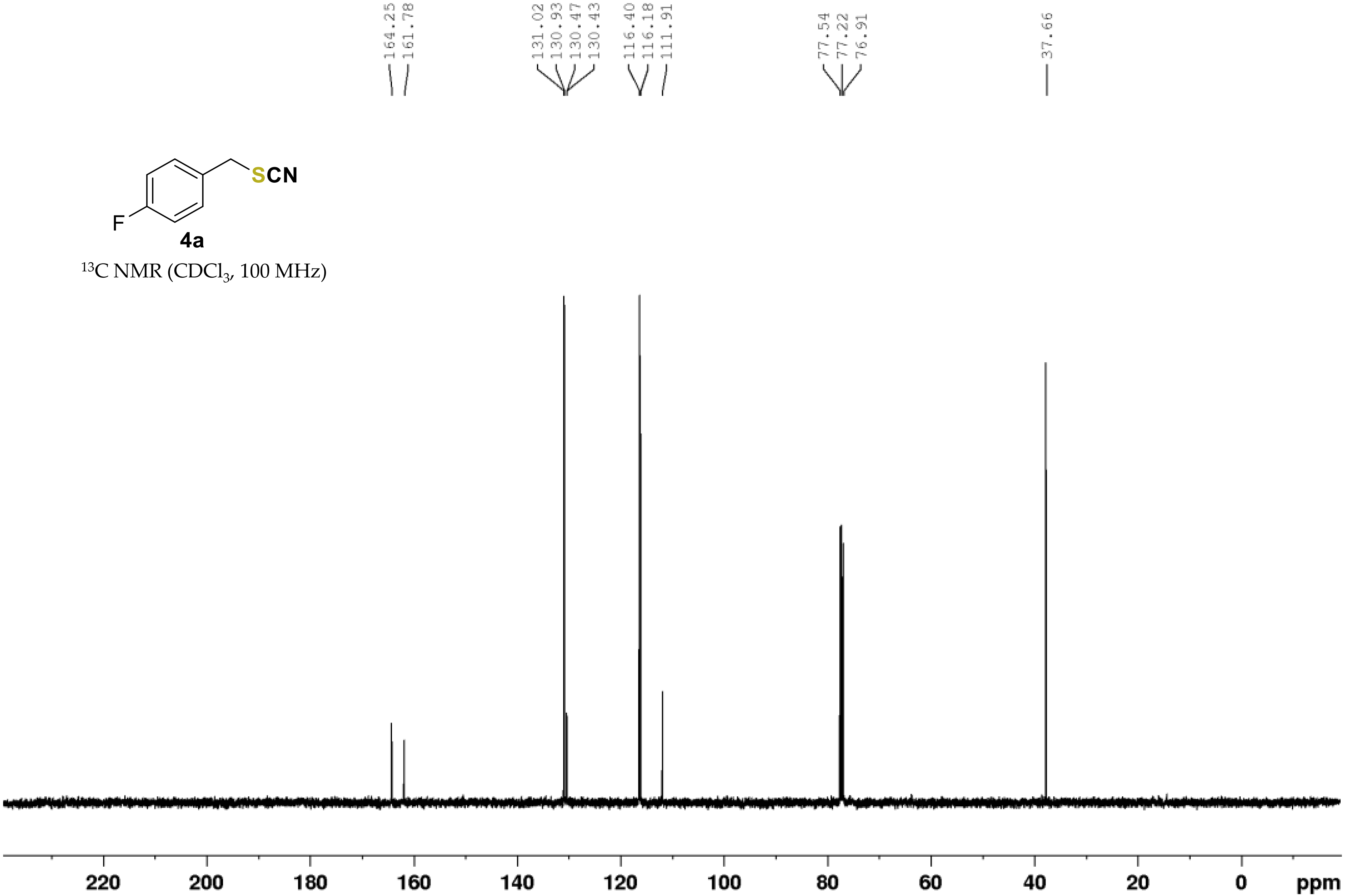

4a

^{13}C NMR ($CDCl_3$, 100 MHz)

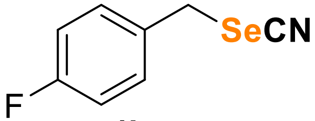

4b

^{1}H NMR ($CDCl_3$, 400 MHz)

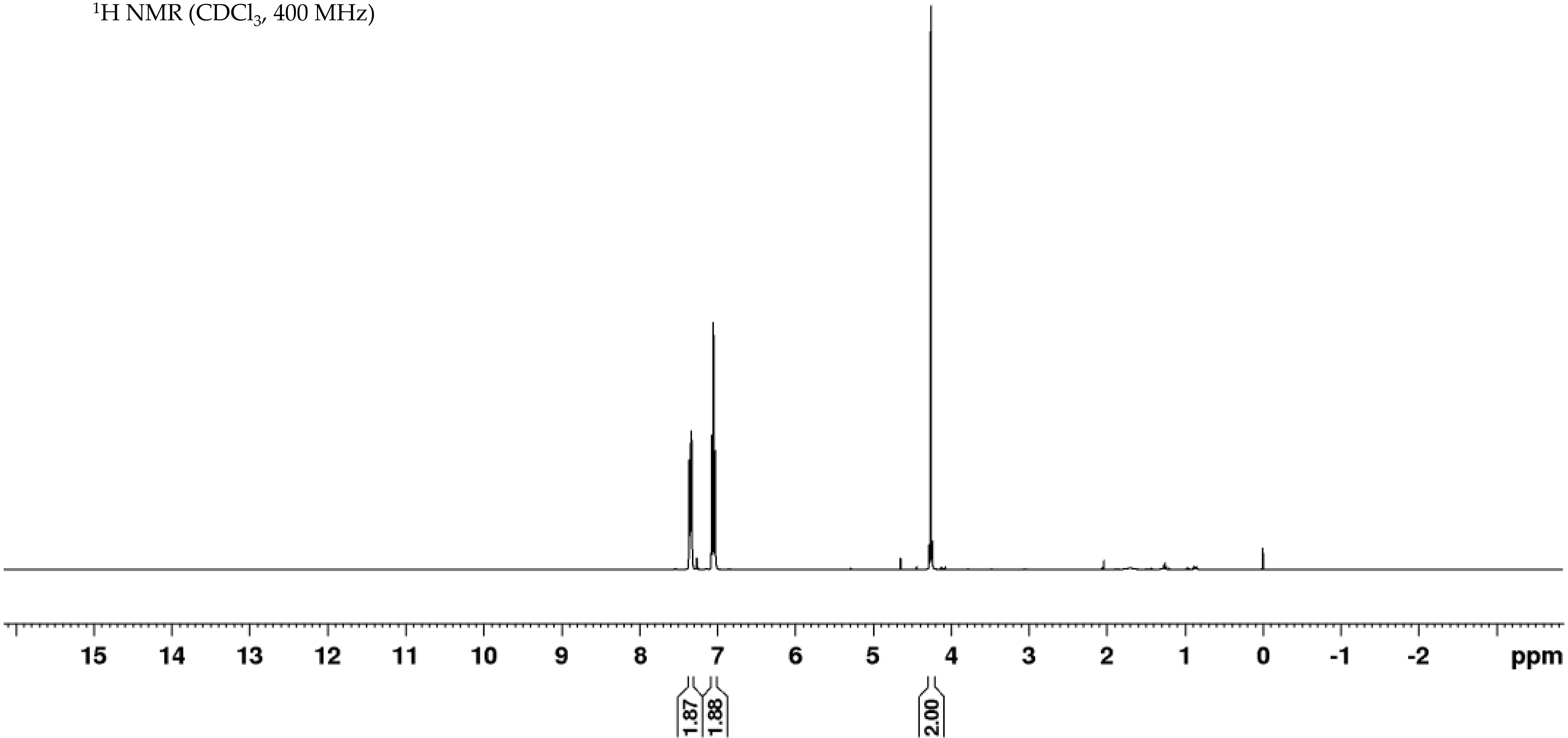

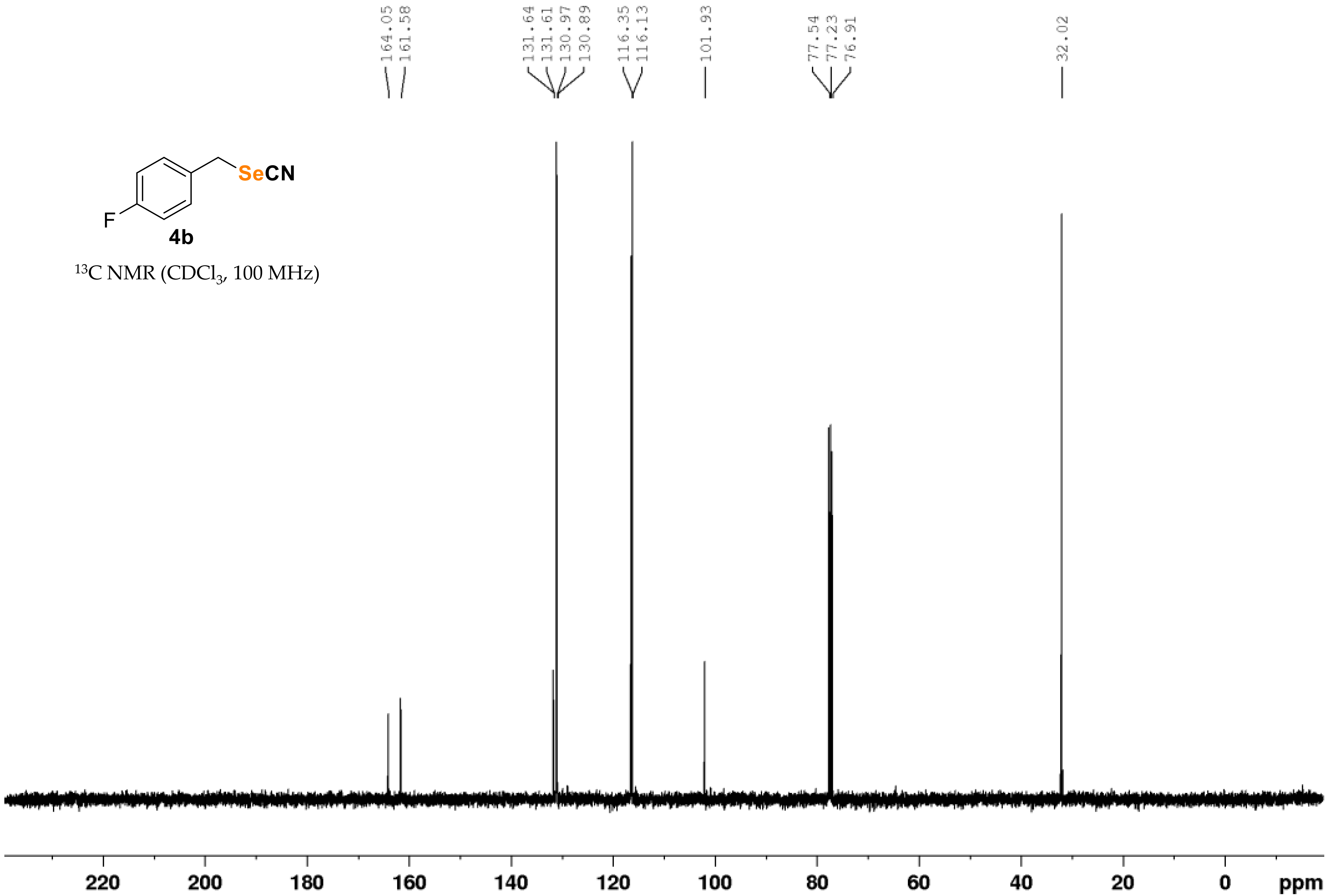
164.05
161.58
131.64
131.61
130.97
130.89
116.35
116.13
101.93
77.54
77.23
76.91
32.02
SeCN
F
4b
^{13}C NMR ($CDCl_3$, 100 MHz)
220
200
180
160
140
120
100
80
60
40
20
0
ppm

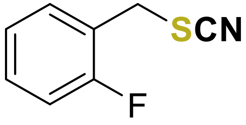

5a

^{1}H NMR ($CDCl_3$, 400 MHz)

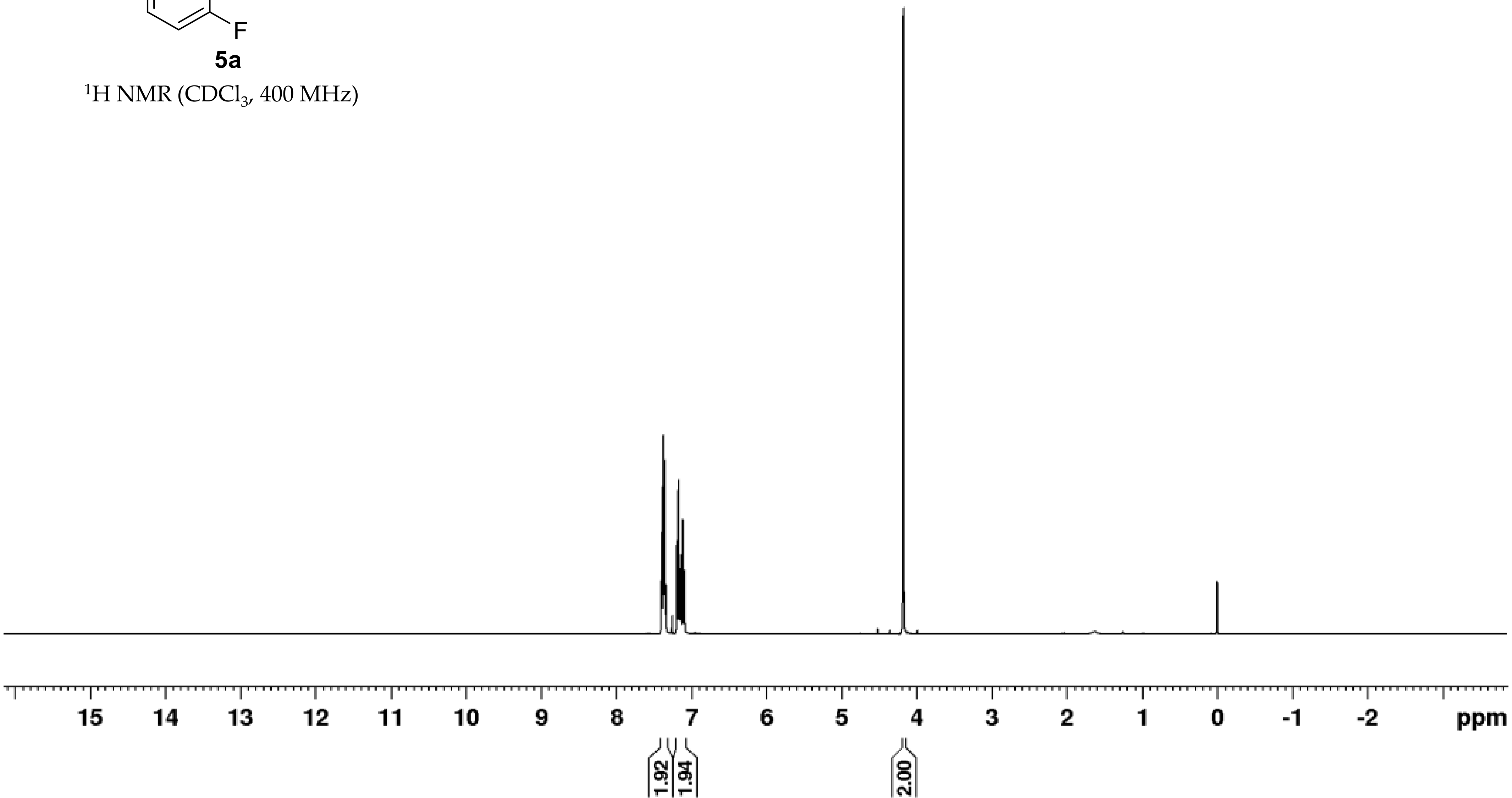

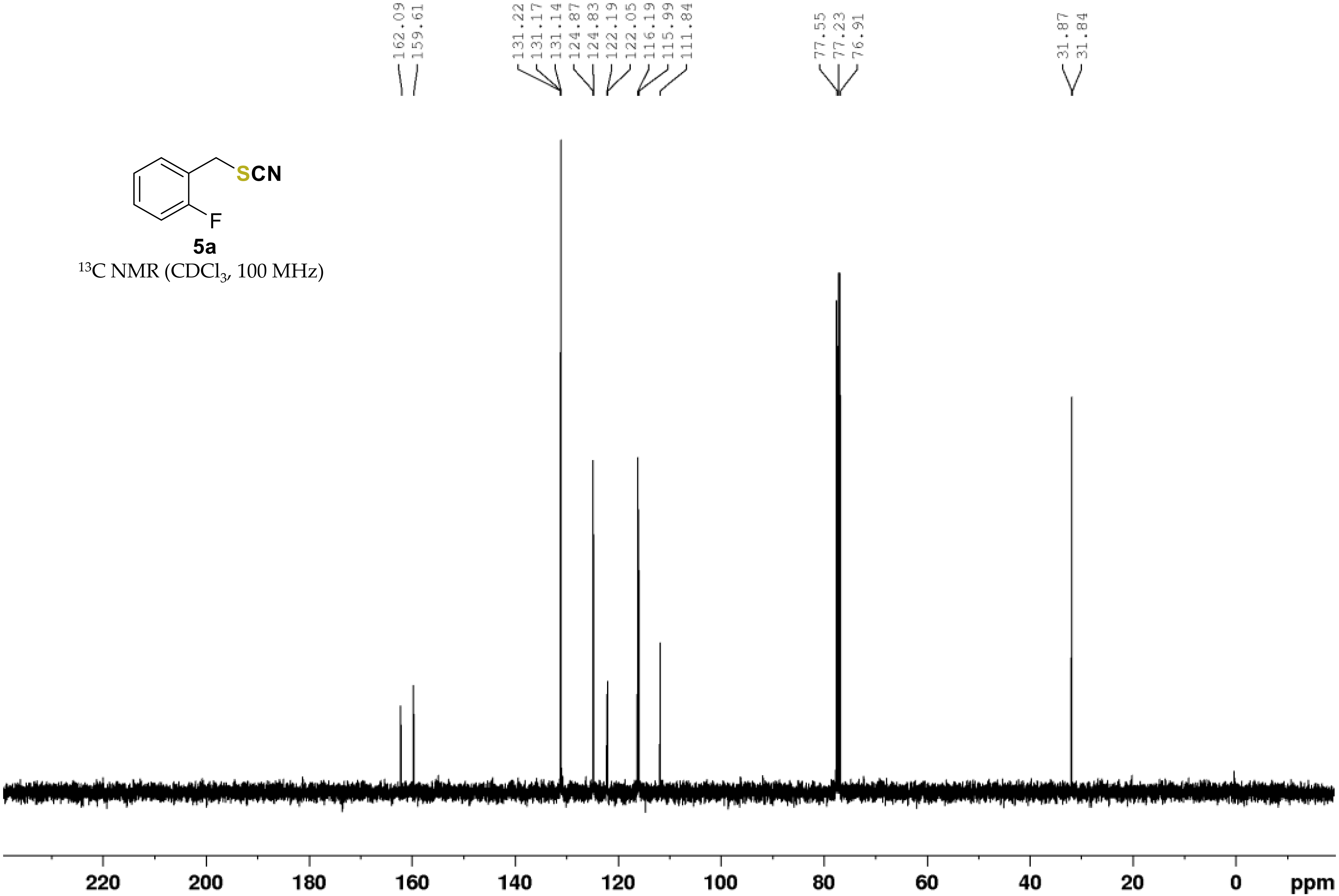

162.09
159.61
131.22
131.17
131.14
124.87
124.83
122.19
122.05
116.19
115.99
111.84
77.55
77.23
76.91
31.87
31.84
SCN
F
5a
^{13}C NMR (CDCl$_3$, 100 MHz)
220
200
180
160
140
120
100
80
60
40
20
0
ppm

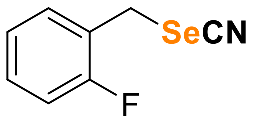

5b

^{1}H NMR ($CDCl_3$, 400 MHz)

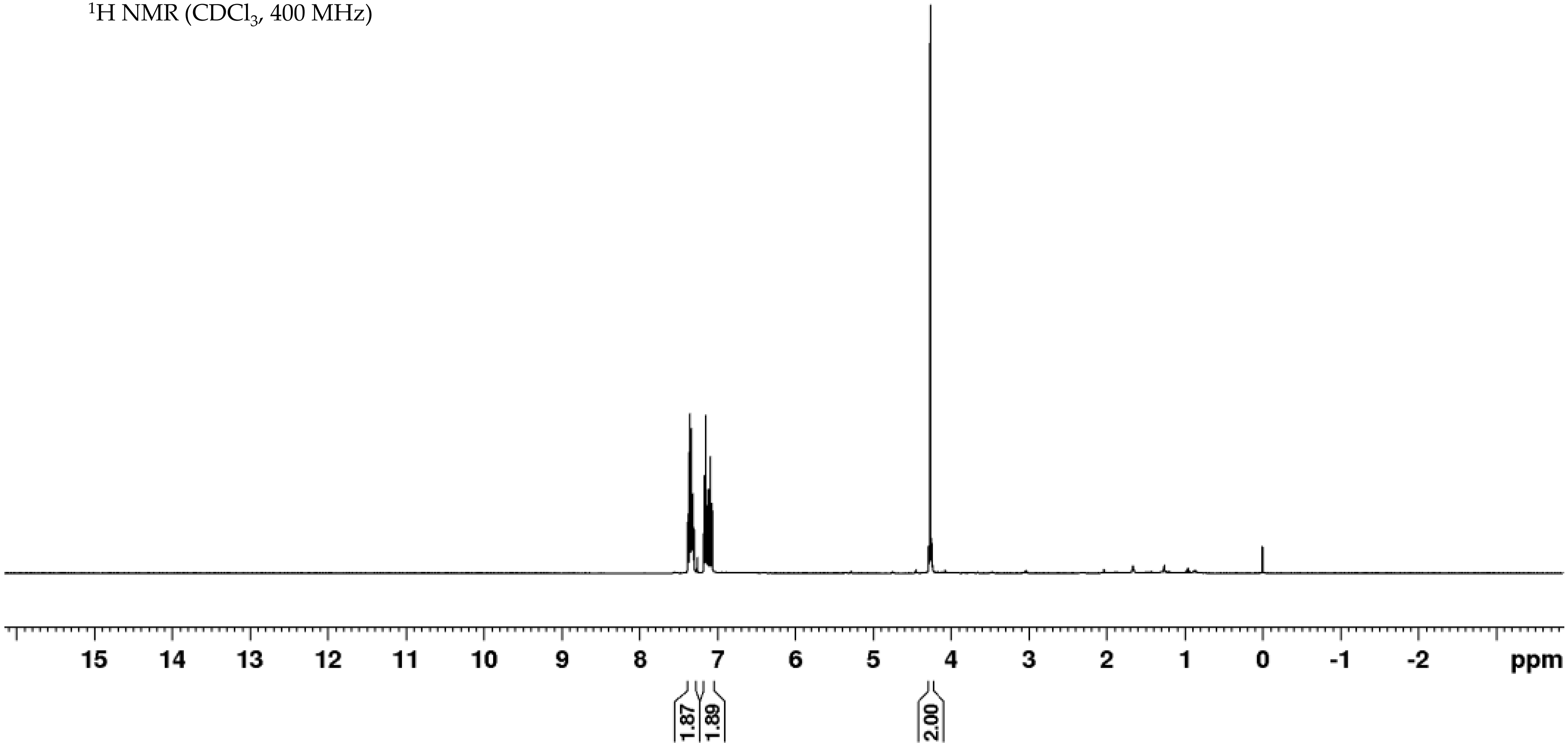

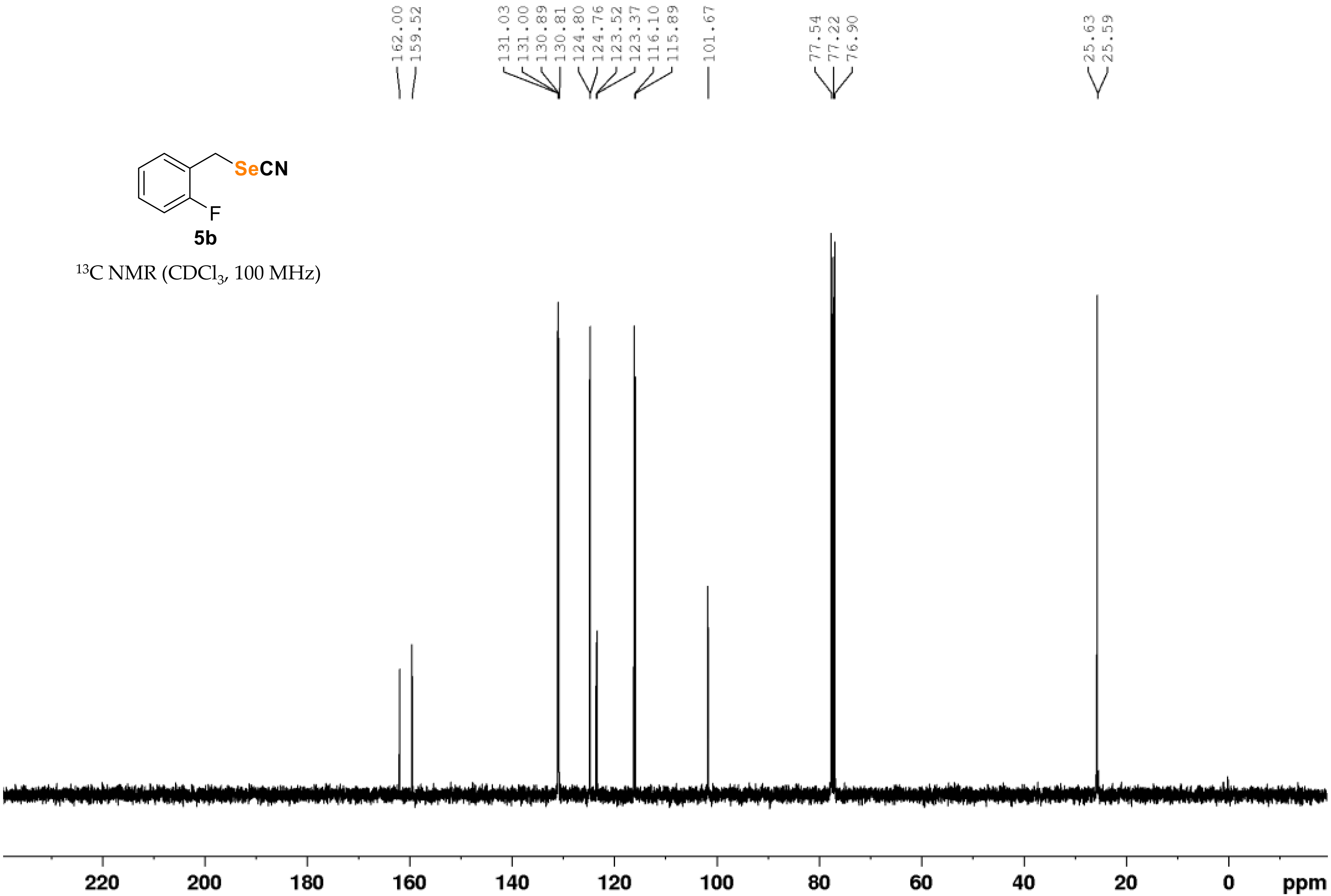

5b

^{13}C NMR ($CDCl_3$, 100 MHz)

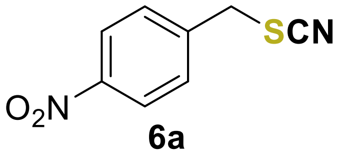

6a

^{1}H NMR ($CDCl_3$, 400 MHz)

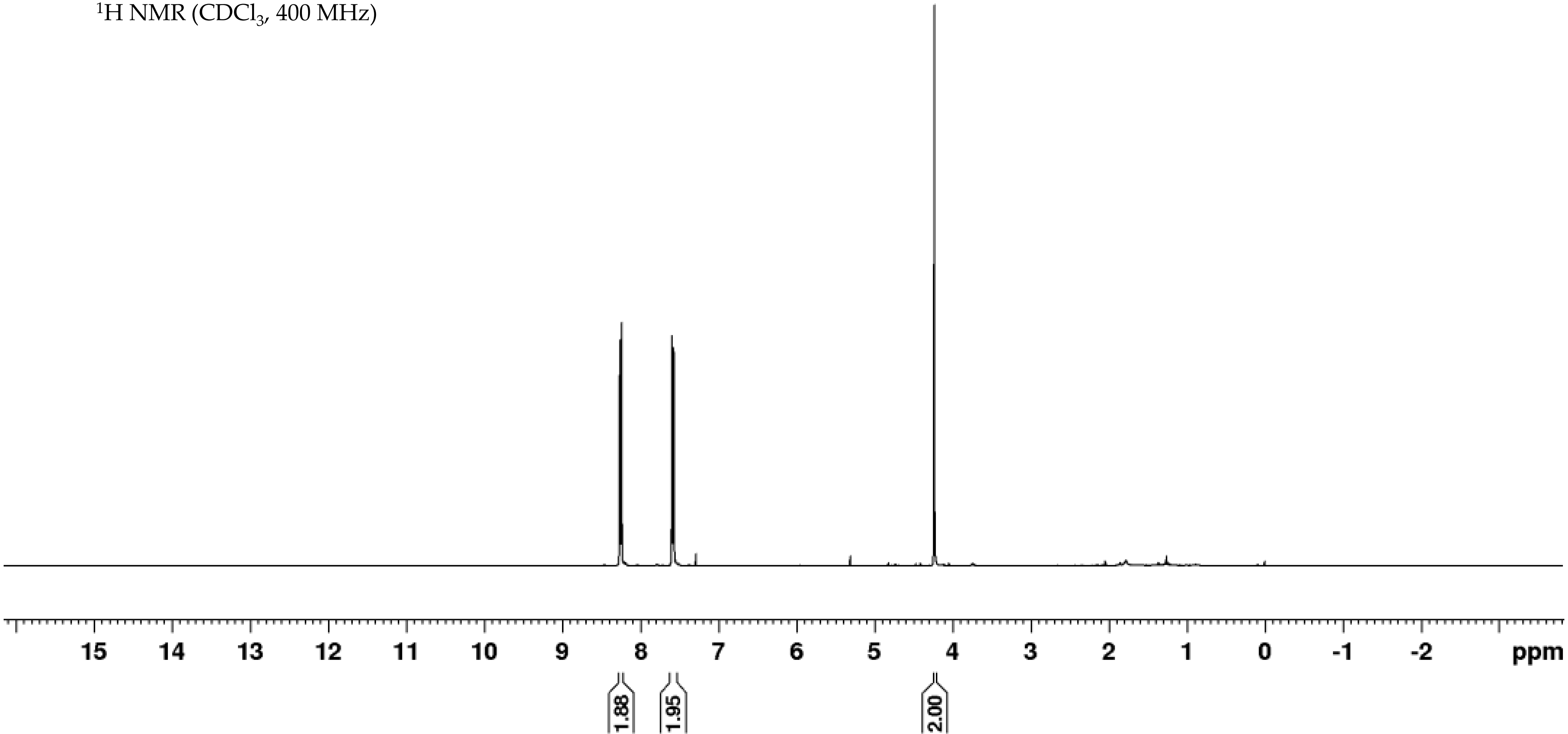

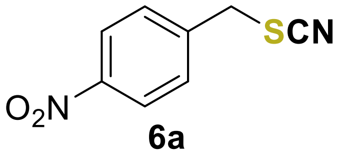

6a

^{13}C NMR ($CDCl_3$, 100 MHz)

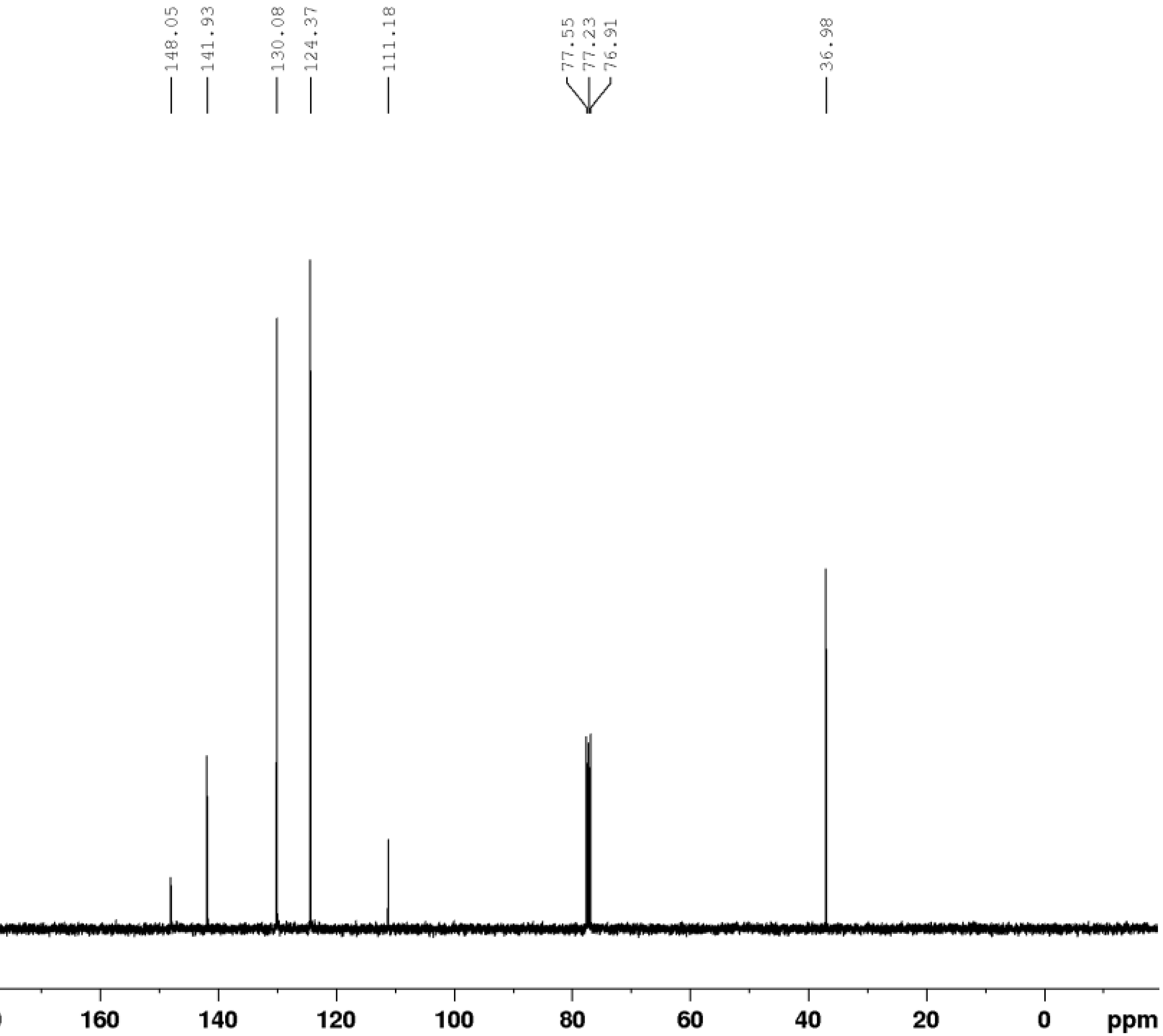

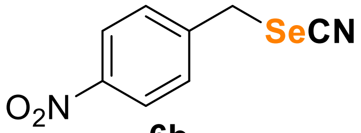

6b

^{1}H NMR ($CDCl_3$, 400 MHz)

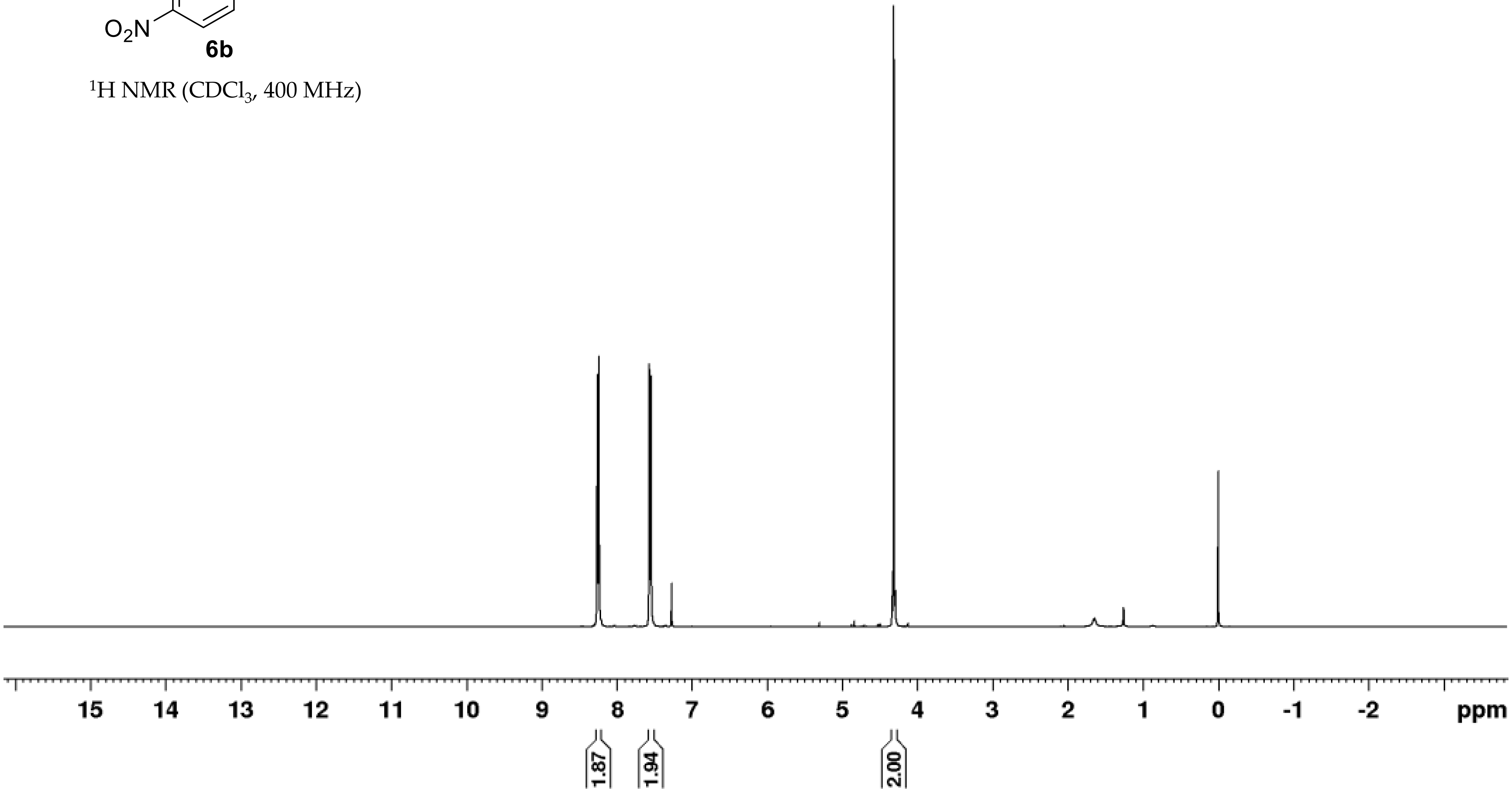

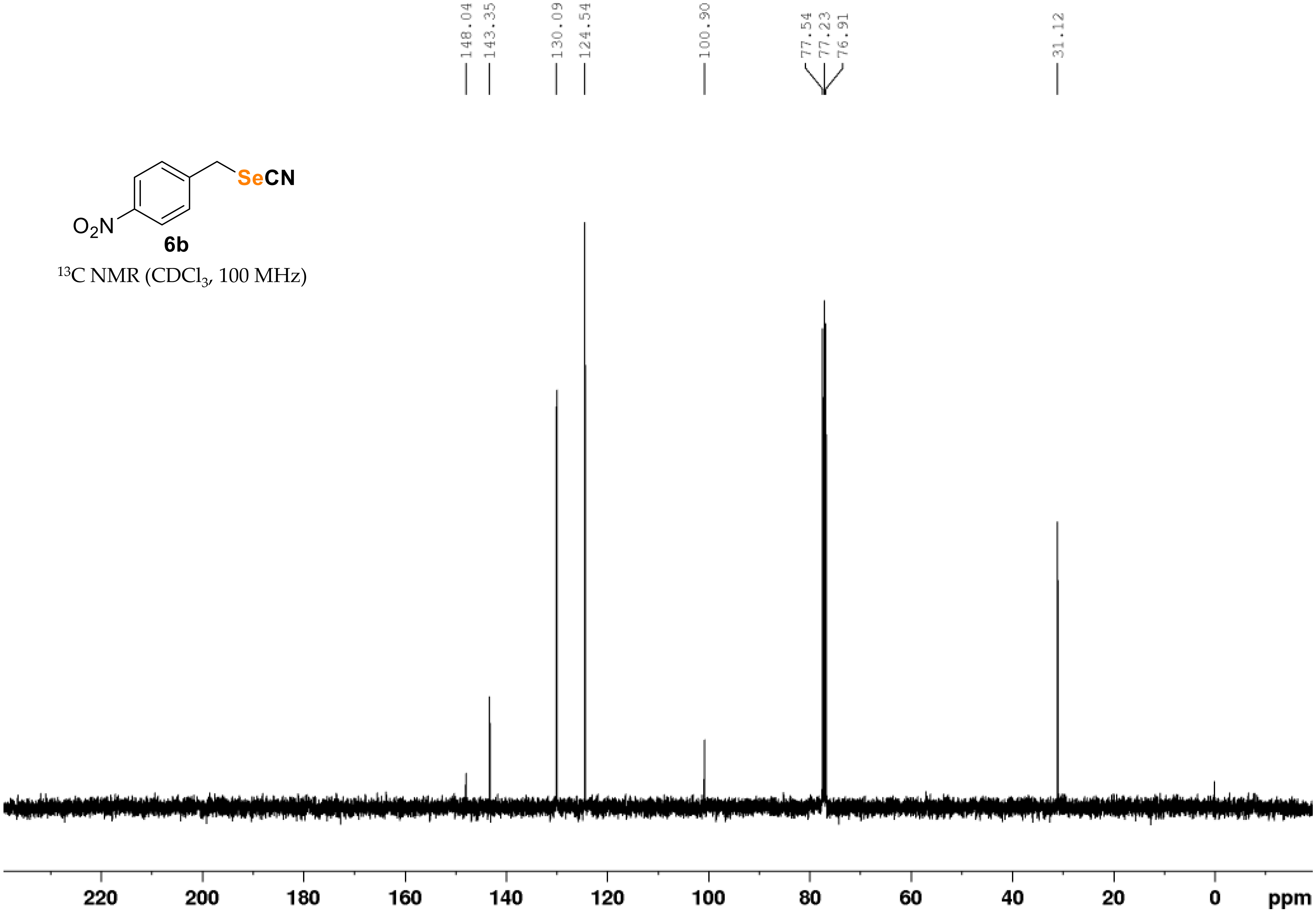

6b

^{13}C NMR ($CDCl_3$, 100 MHz)

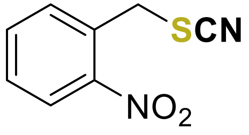

7a

^{1}H NMR ($CDCl_3$, 400 MHz)

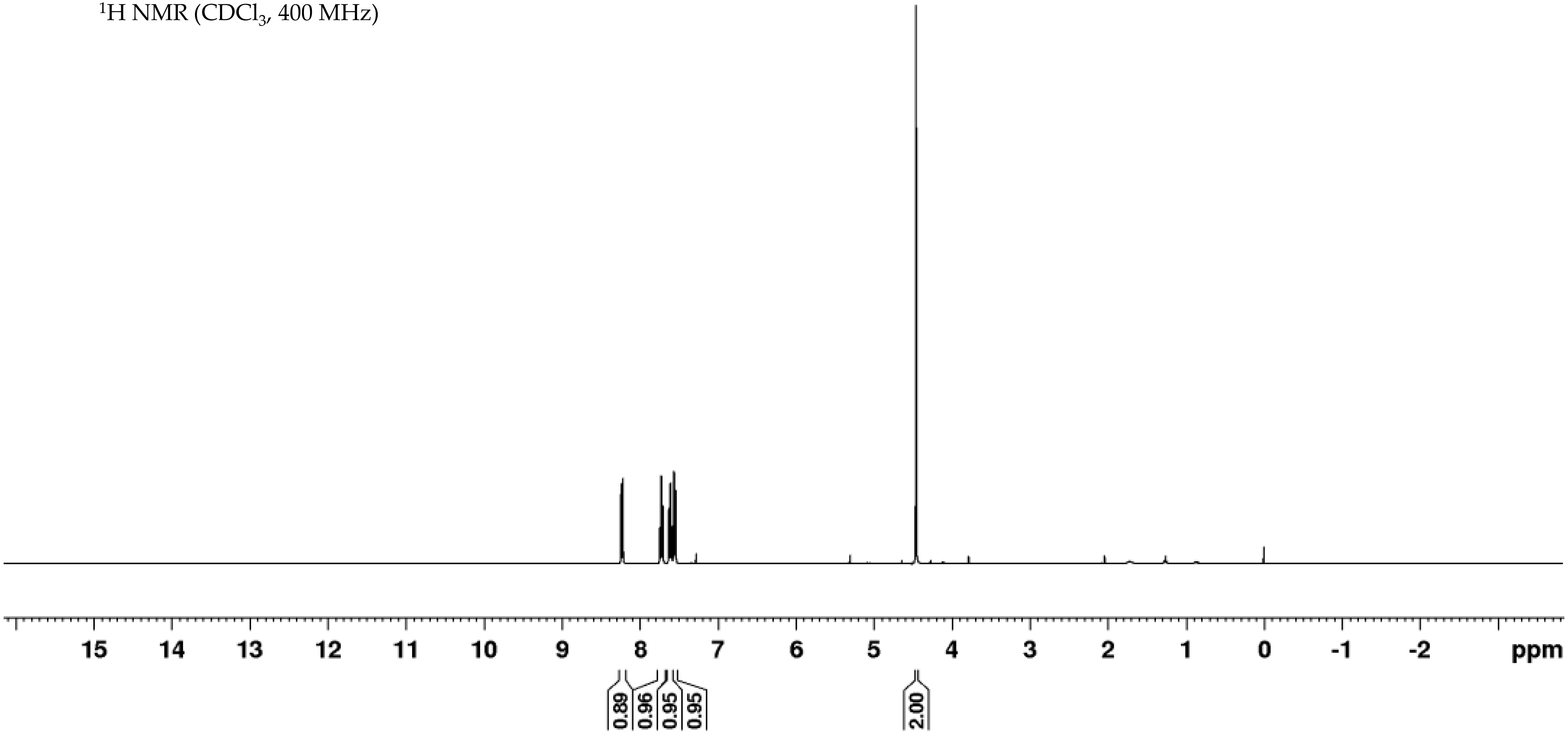

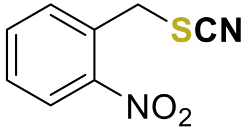

7a

^{13}C NMR($CDCl_3$, 100 MHz)

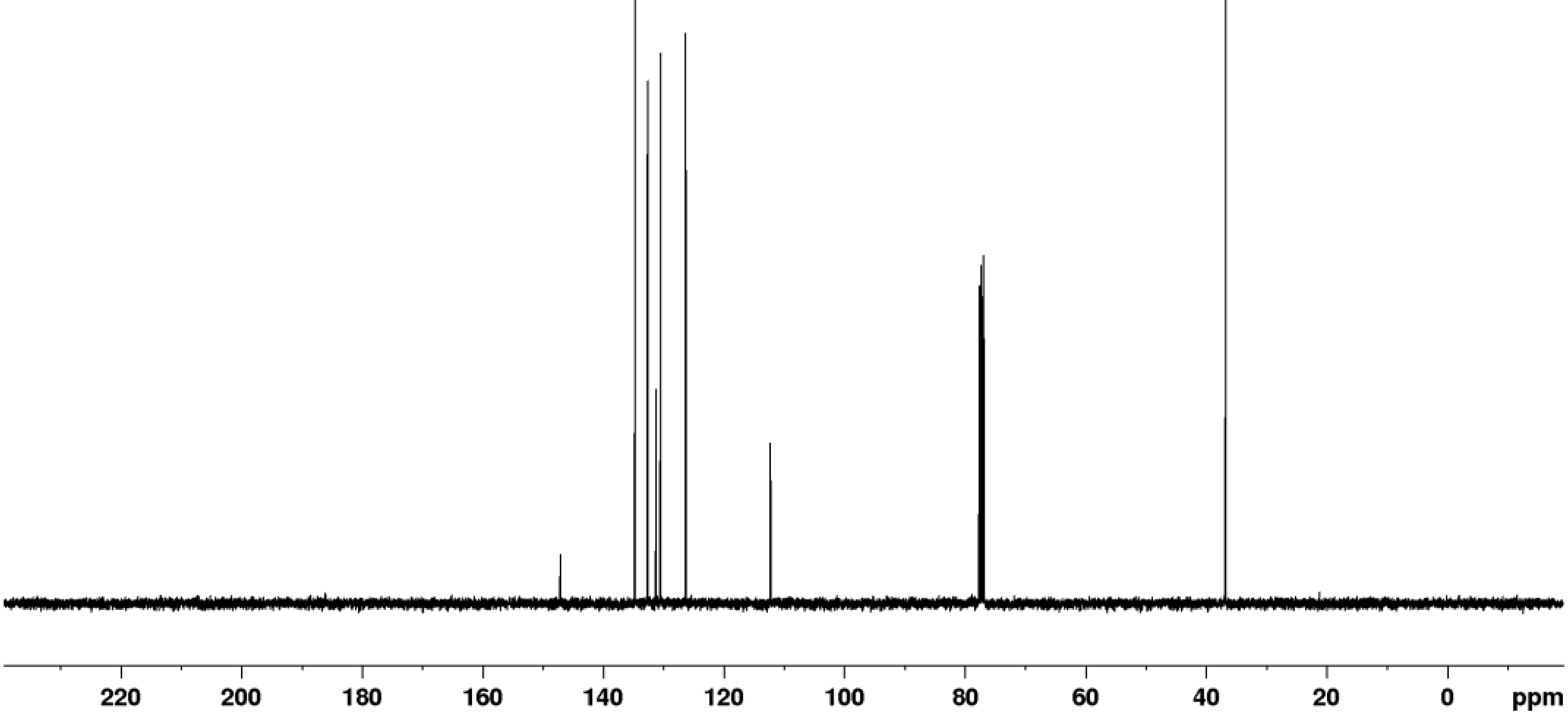

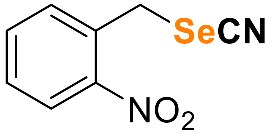

7b

^{1}H NMR ($CDCl_3$, 400 MHz)

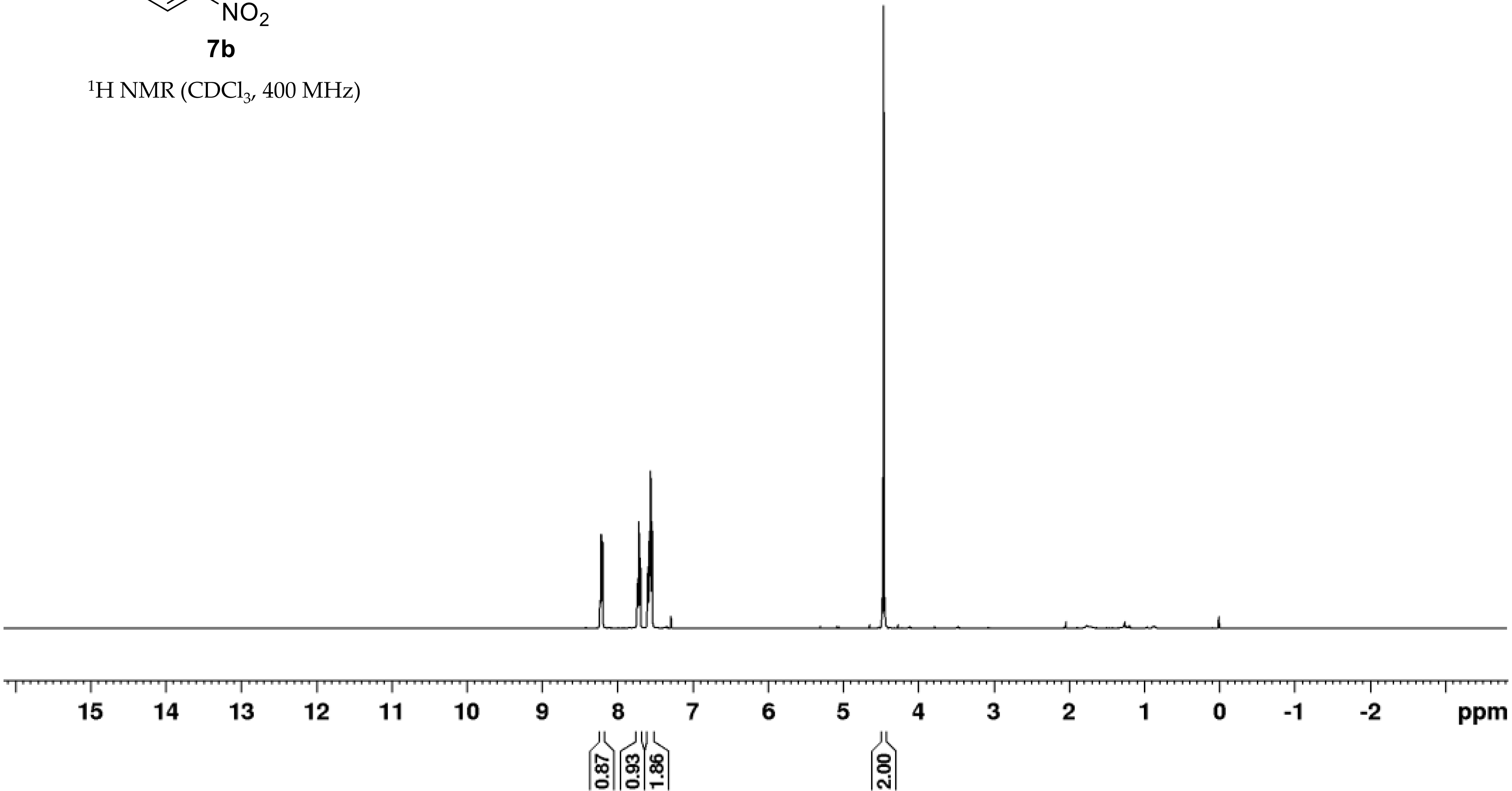

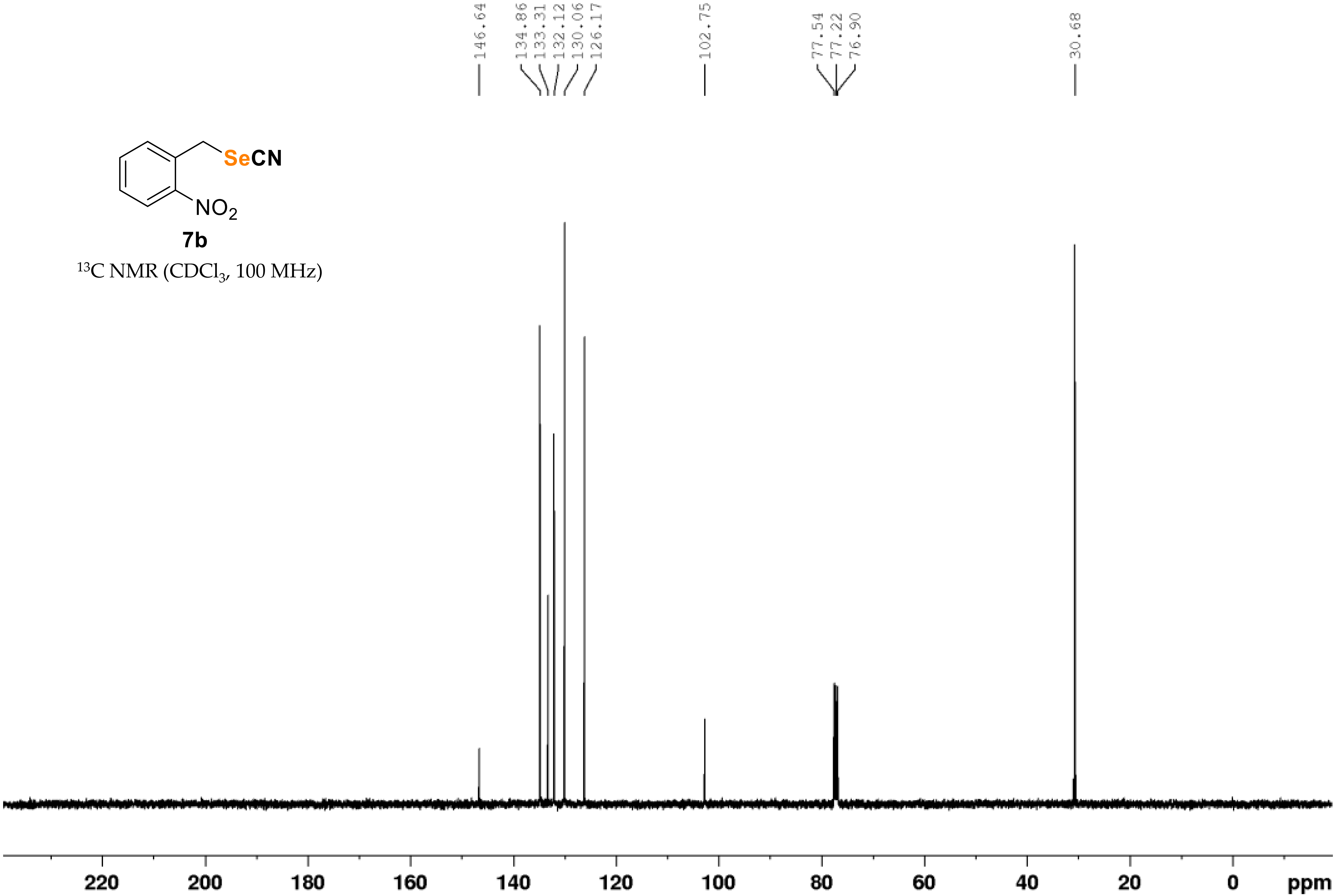

7b

^{13}C NMR ($CDCl_3$, 100 MHz)

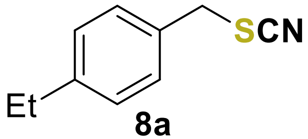

8a

^{1}H NMR ($CDCl_3$, 400 MHz)

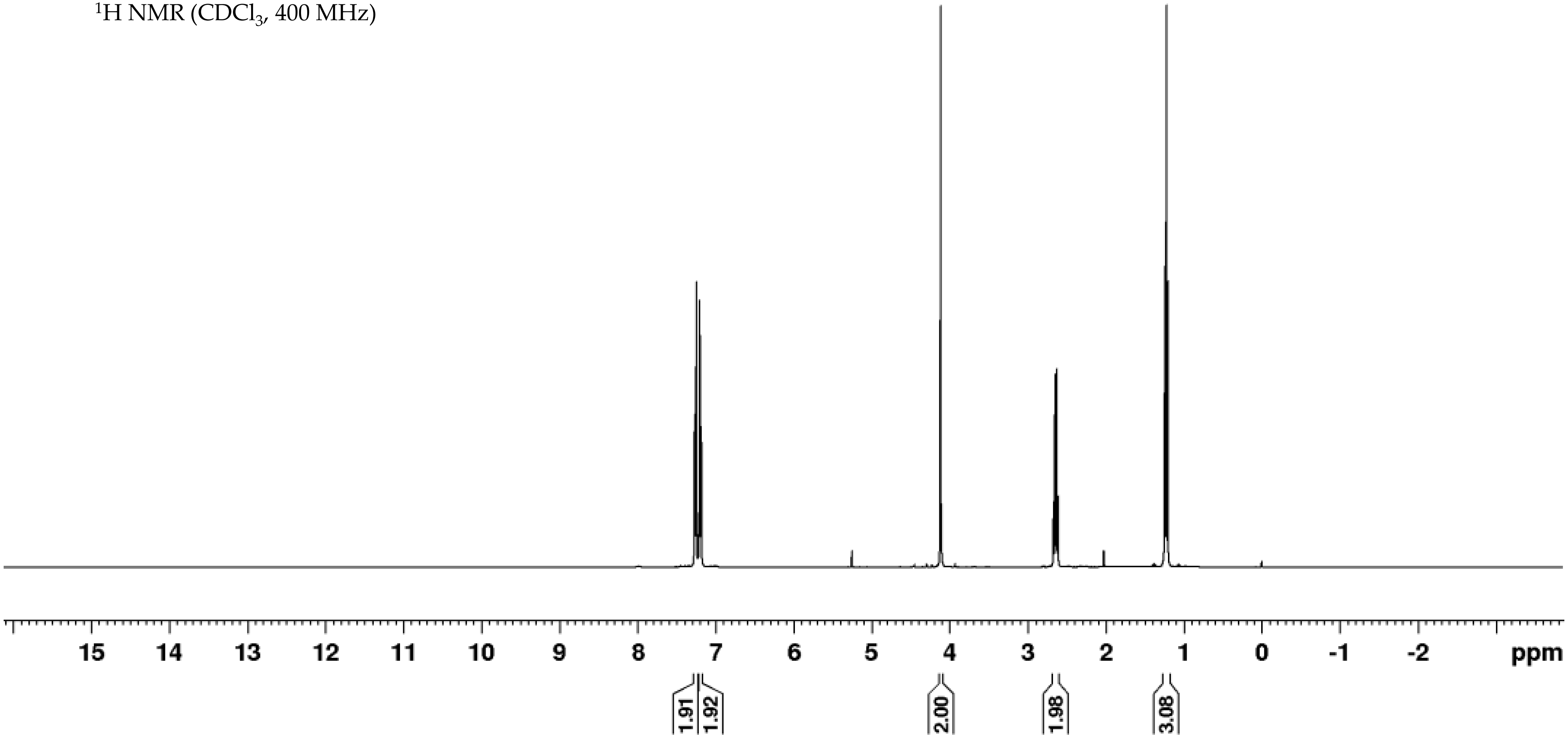

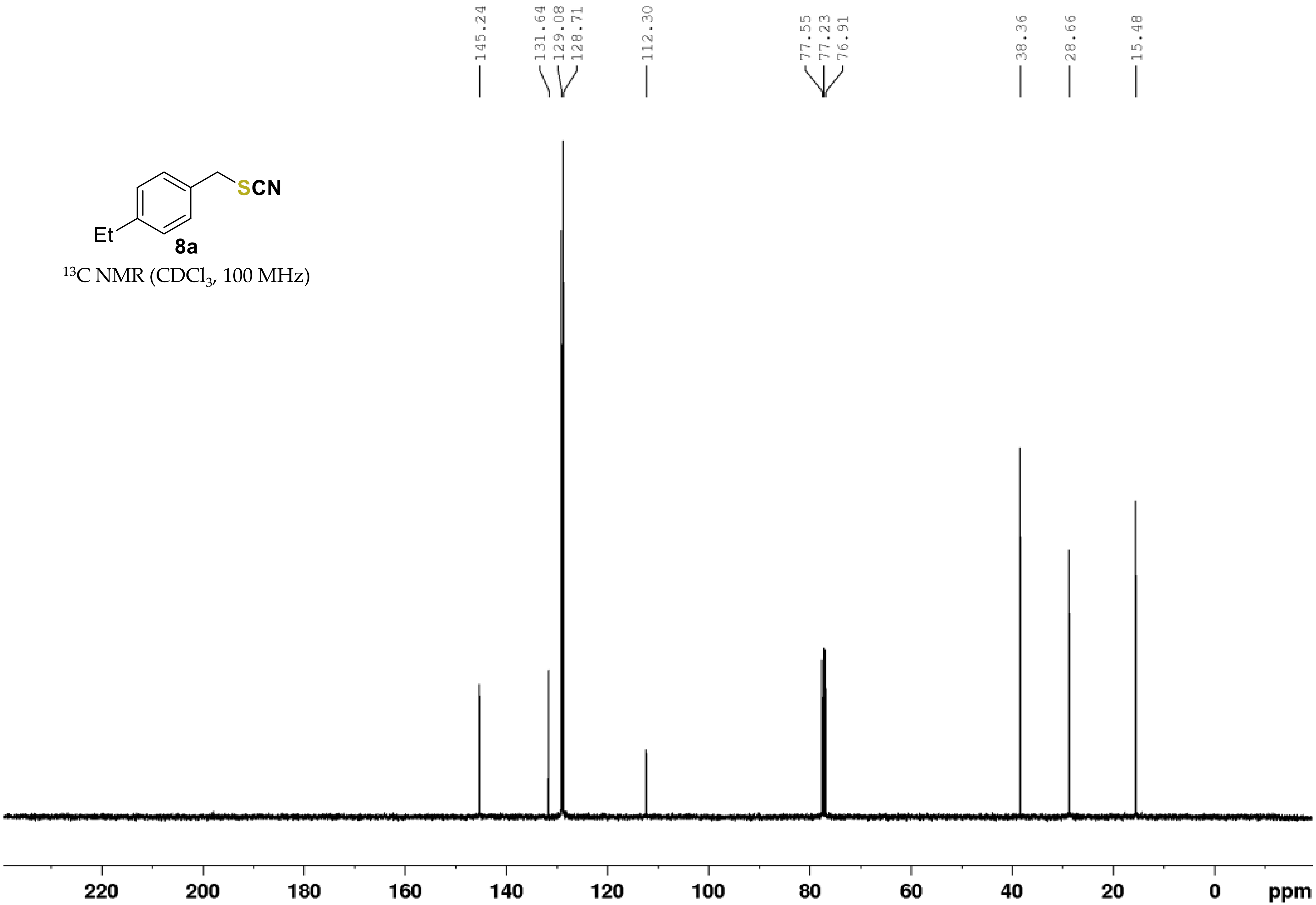
145.24
131.64
129.08
128.71
112.30
77.55
77.23
76.91
38.36
28.66
15.48
SCN
Et
8a
13C NMR (CDCl3, 100 MHz)
220
200
180
160
140
120
100
80
60
40
20
0
ppm

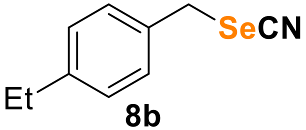

8b

^{1}H NMR ($CDCl_3$, 400 MHz)

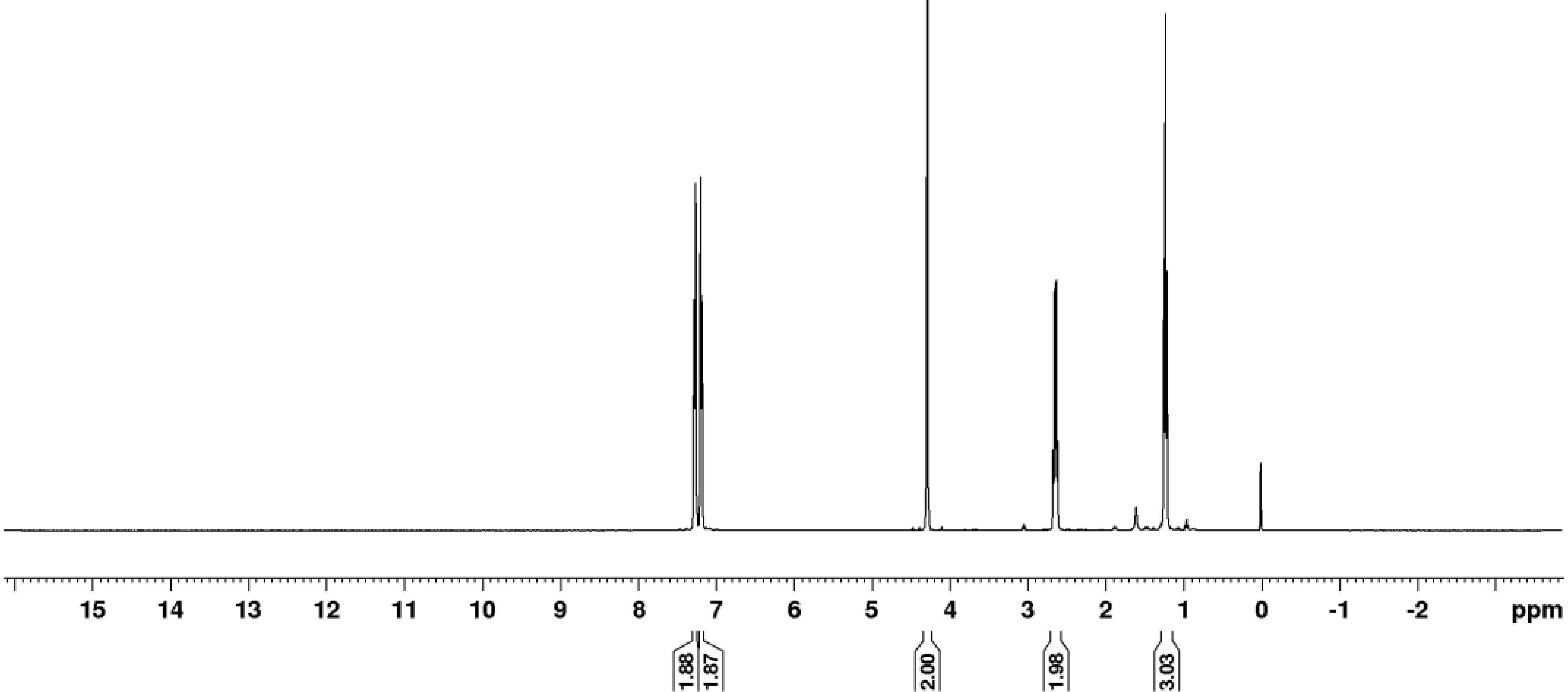

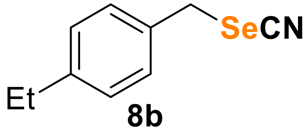

8b

^{13}C NMR ($CDCl_3$, 100 MHz)

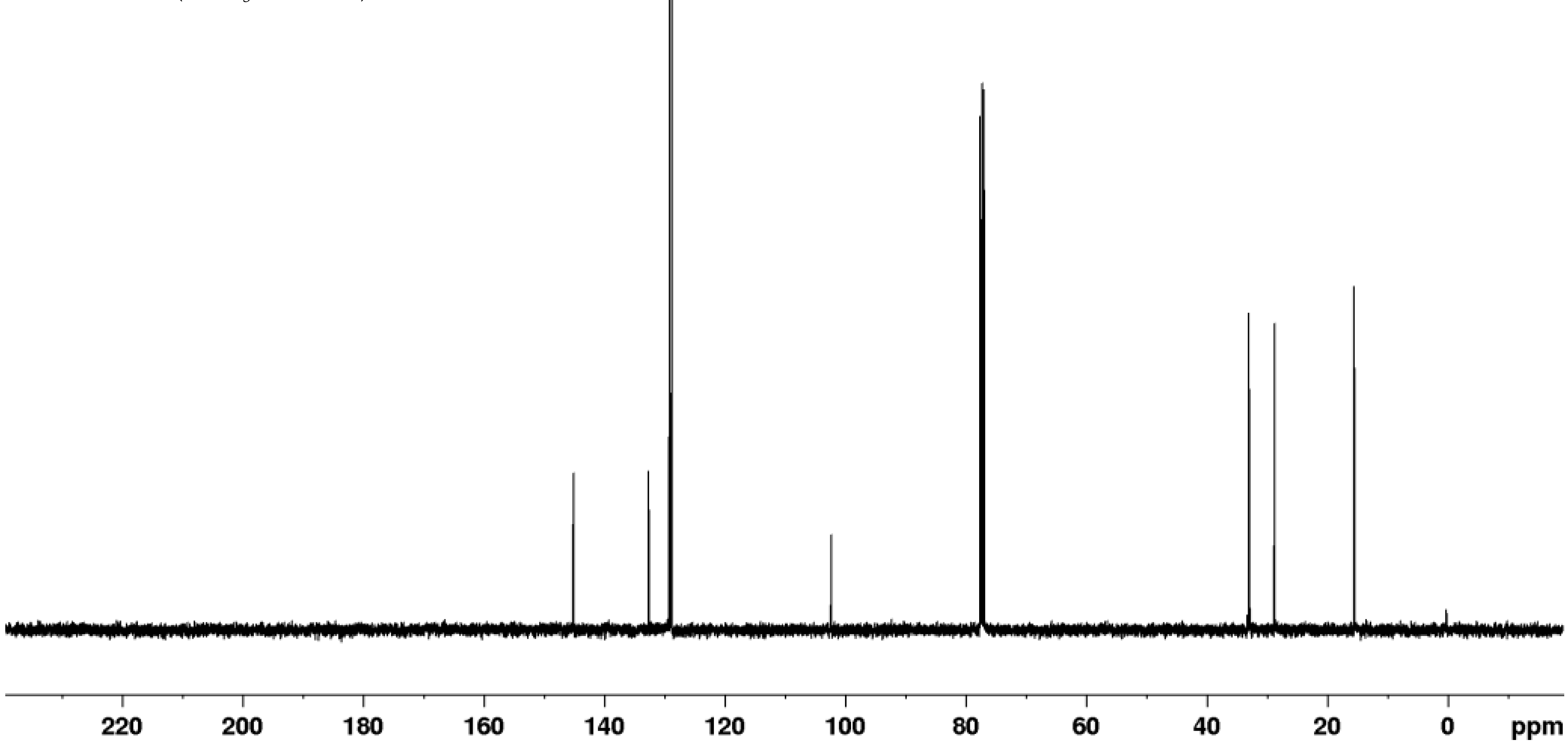

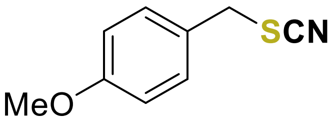

9a

^{1}H NMR ($CDCl_3$, 400 MHz)

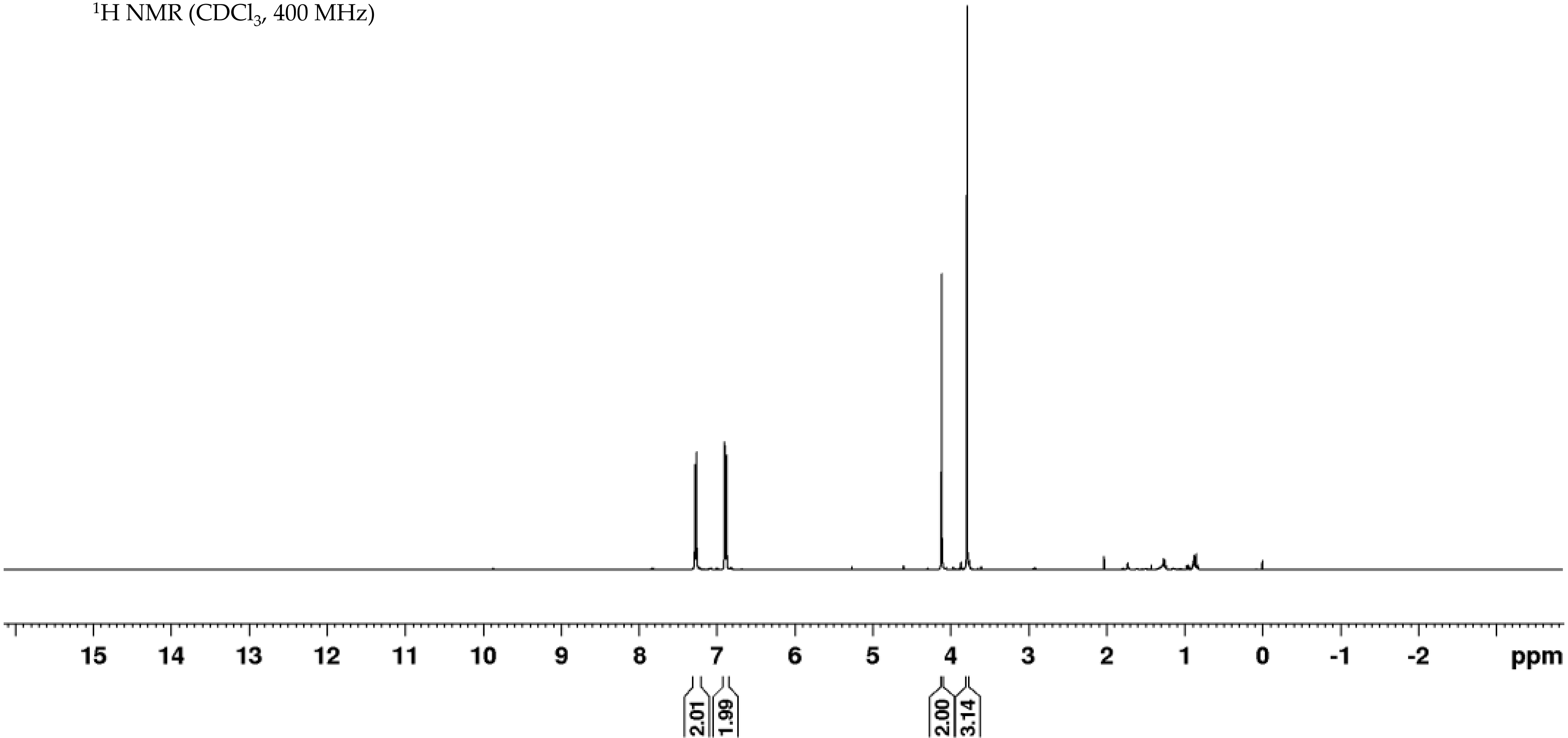

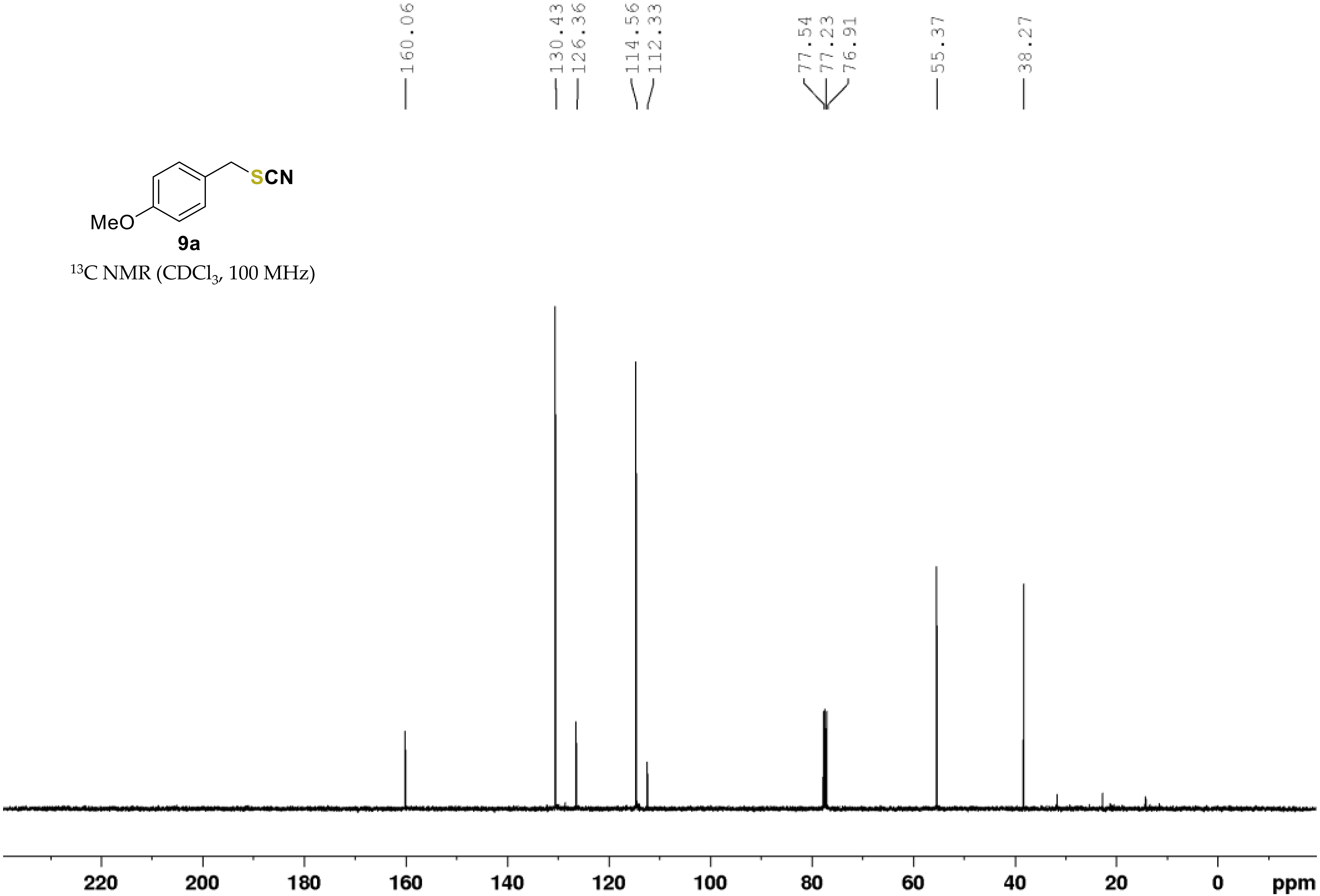

160.06
130.43
126.36
114.56
112.33
77.54
77.23
76.91
55.37
38.27
SCN
MeO
9a
^{13}C NMR ($CDCl_3$, 100 MHz)
220
200
180
160
140
120
100
80
60
40
20
0
ppm

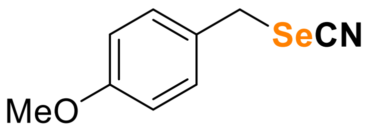

9b

^{1}H NMR ($CDCl_3$, 400 MHz)

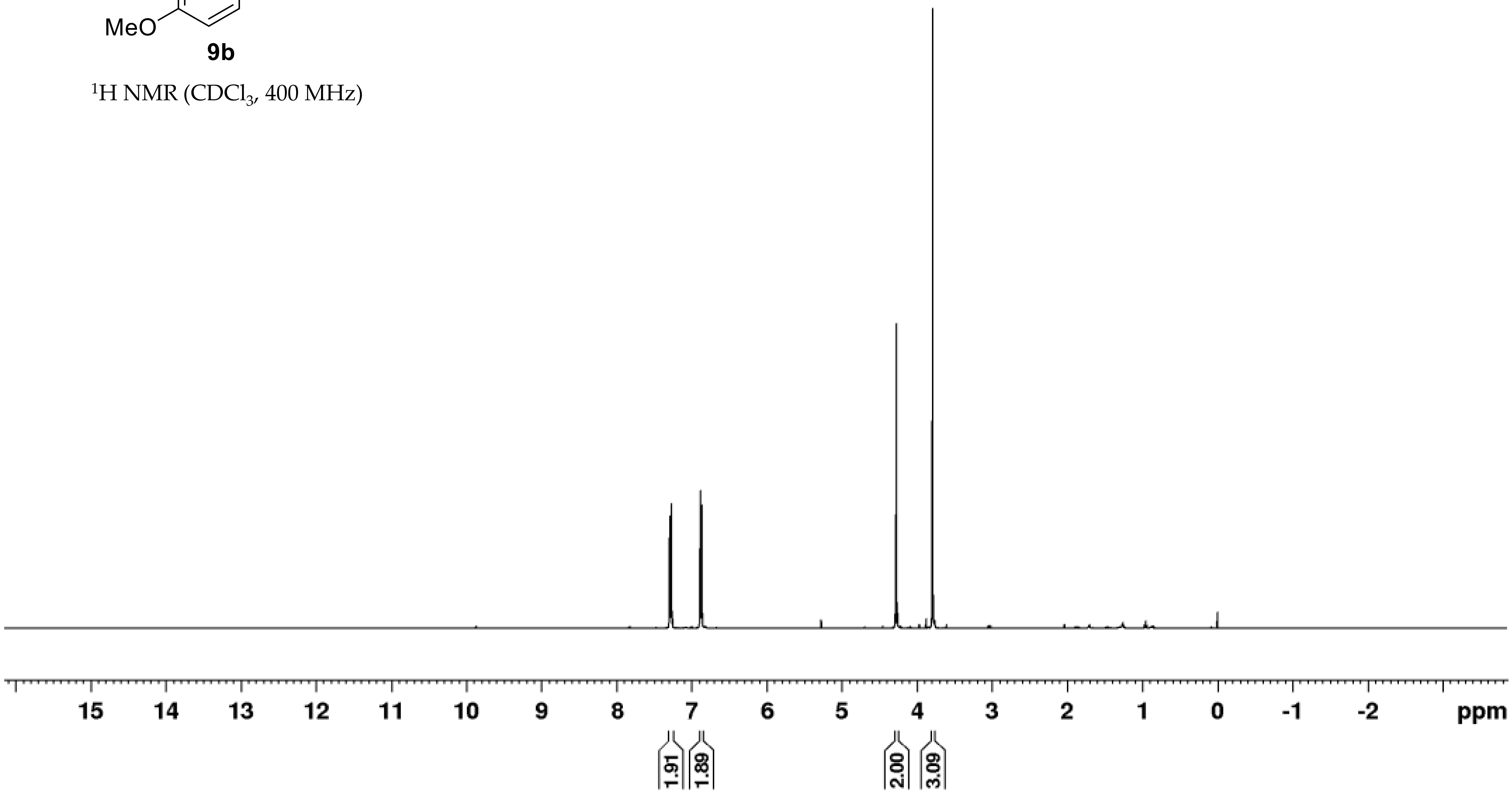

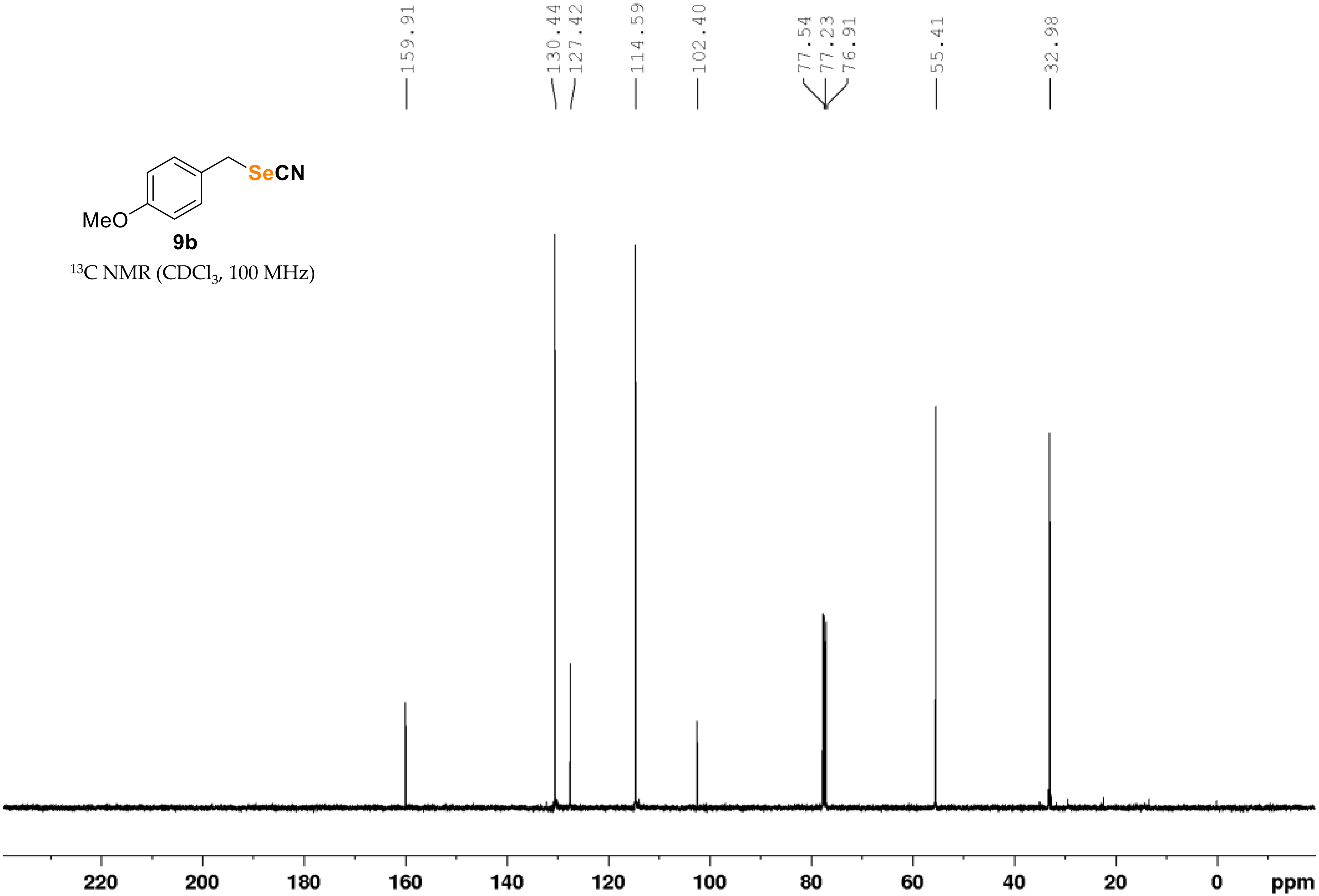

^{13}C NMR ($CDCl_3$, 100 MHz)

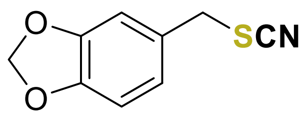

10a

^{1}H NMR ($CDCl_3$, 400 MHz)

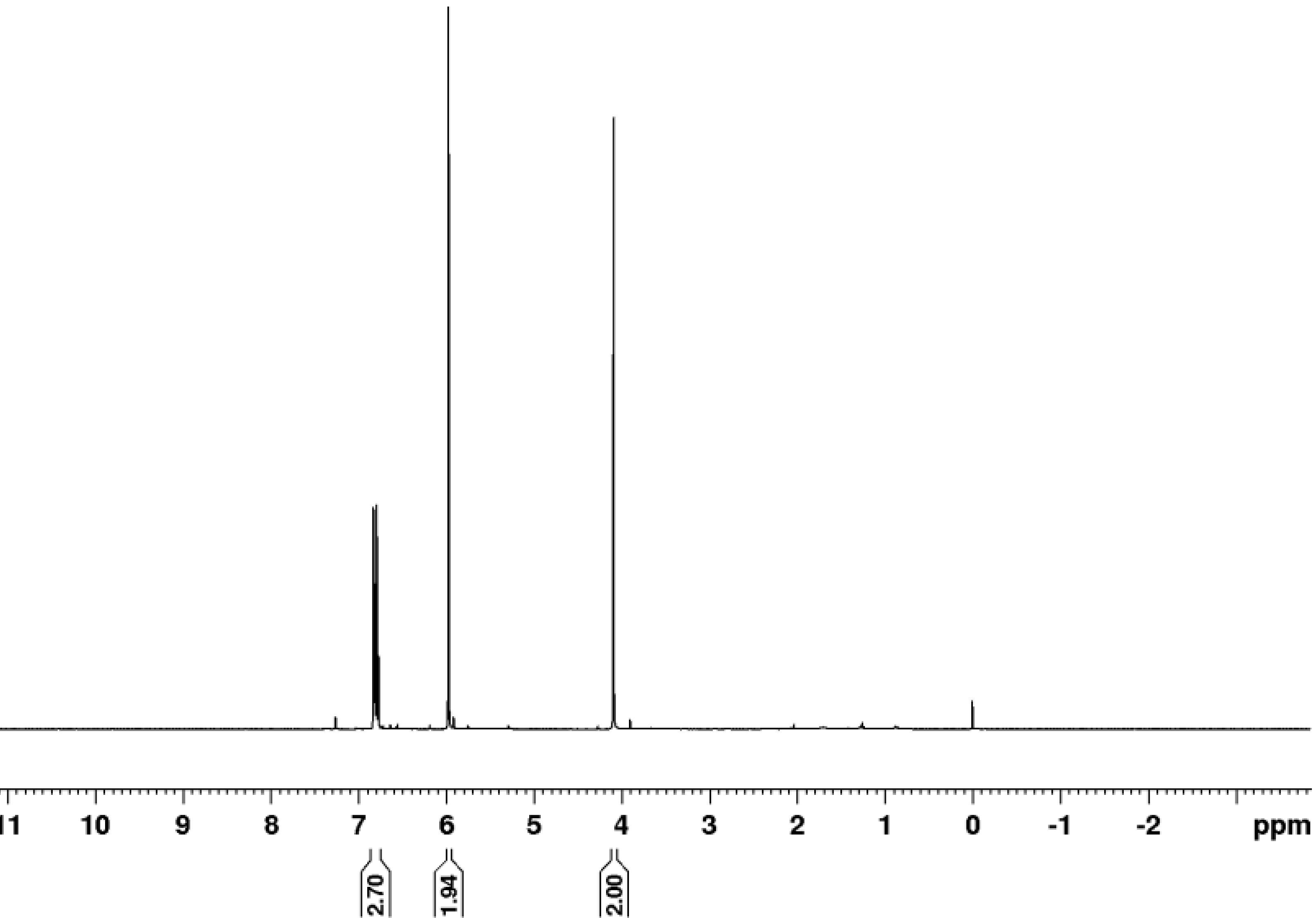

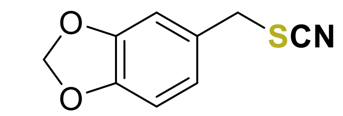

10a

^{13}C NMR ($CDCl_3$, 100 MHz)

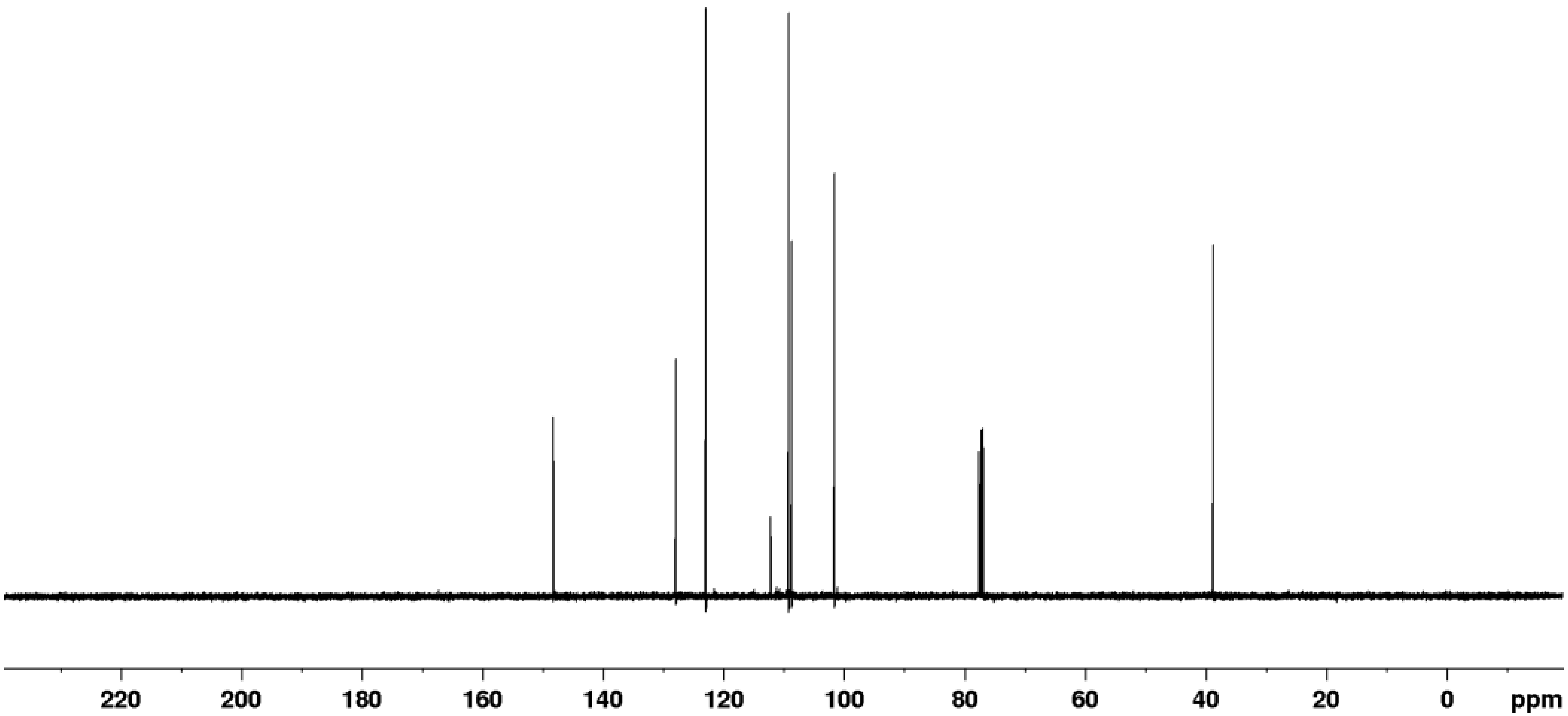

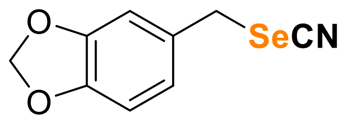

10b

^{1}H NMR ($CDCl_3$, 400 MHz)

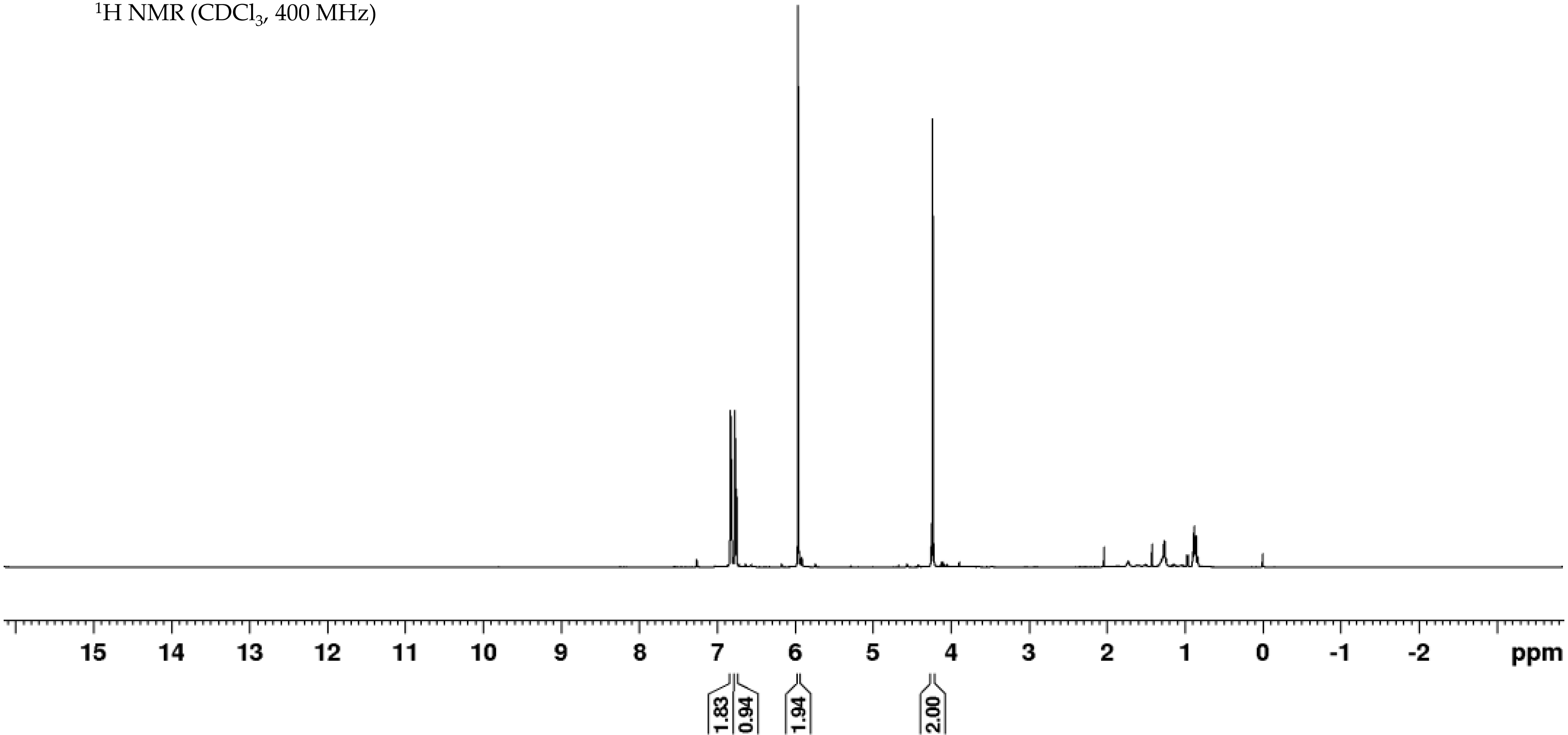

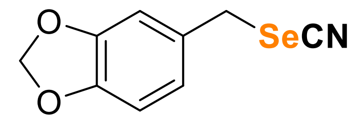

10b

^{13}C NMR ($CDCl_3$, 100 MHz)

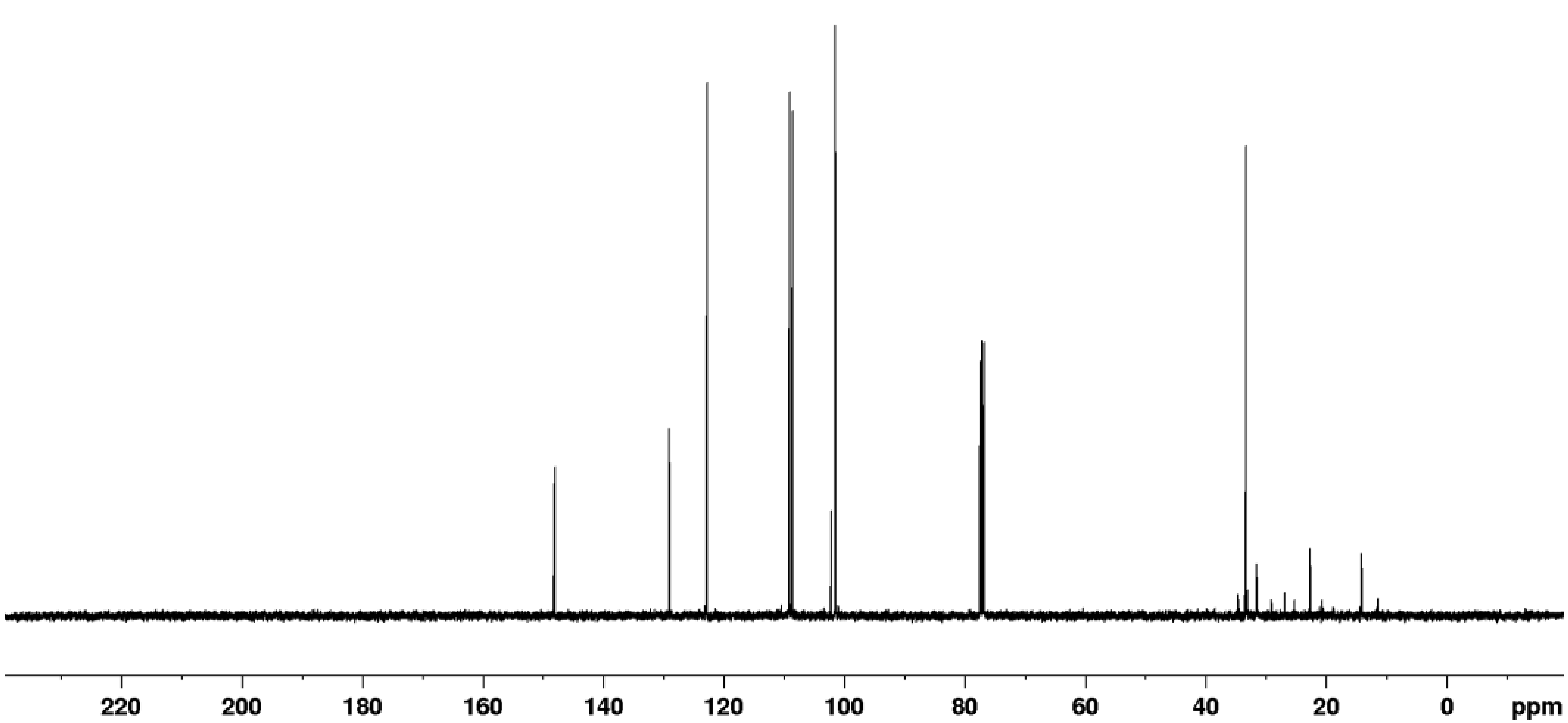

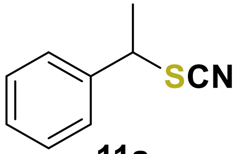

11a

^{1}H NMR ($CDCl_3$, 400 MHz)

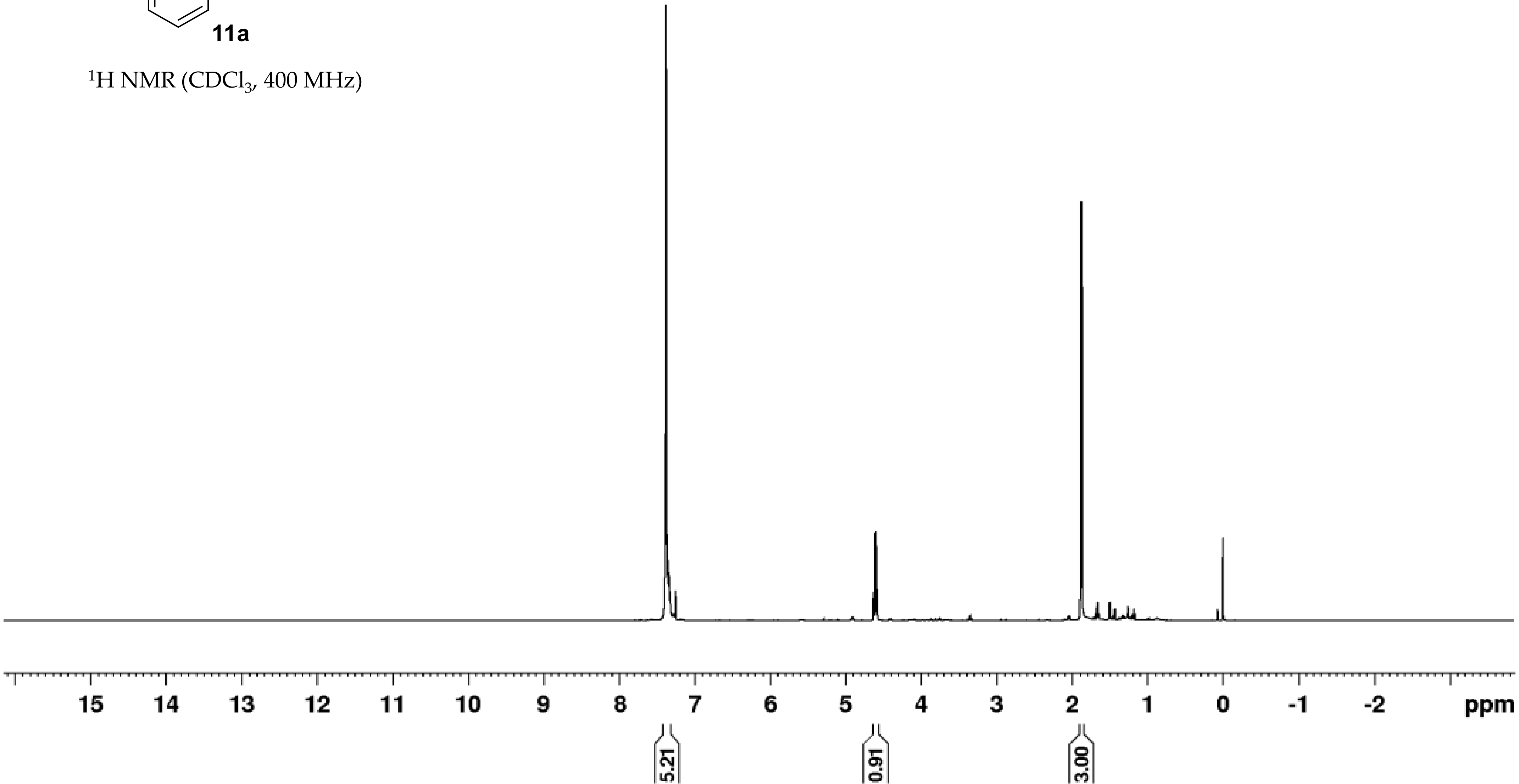

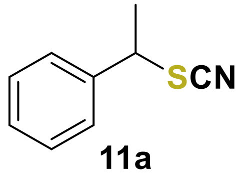

11a

^{13}C NMR ($CDCl_3$, 100 MHz)

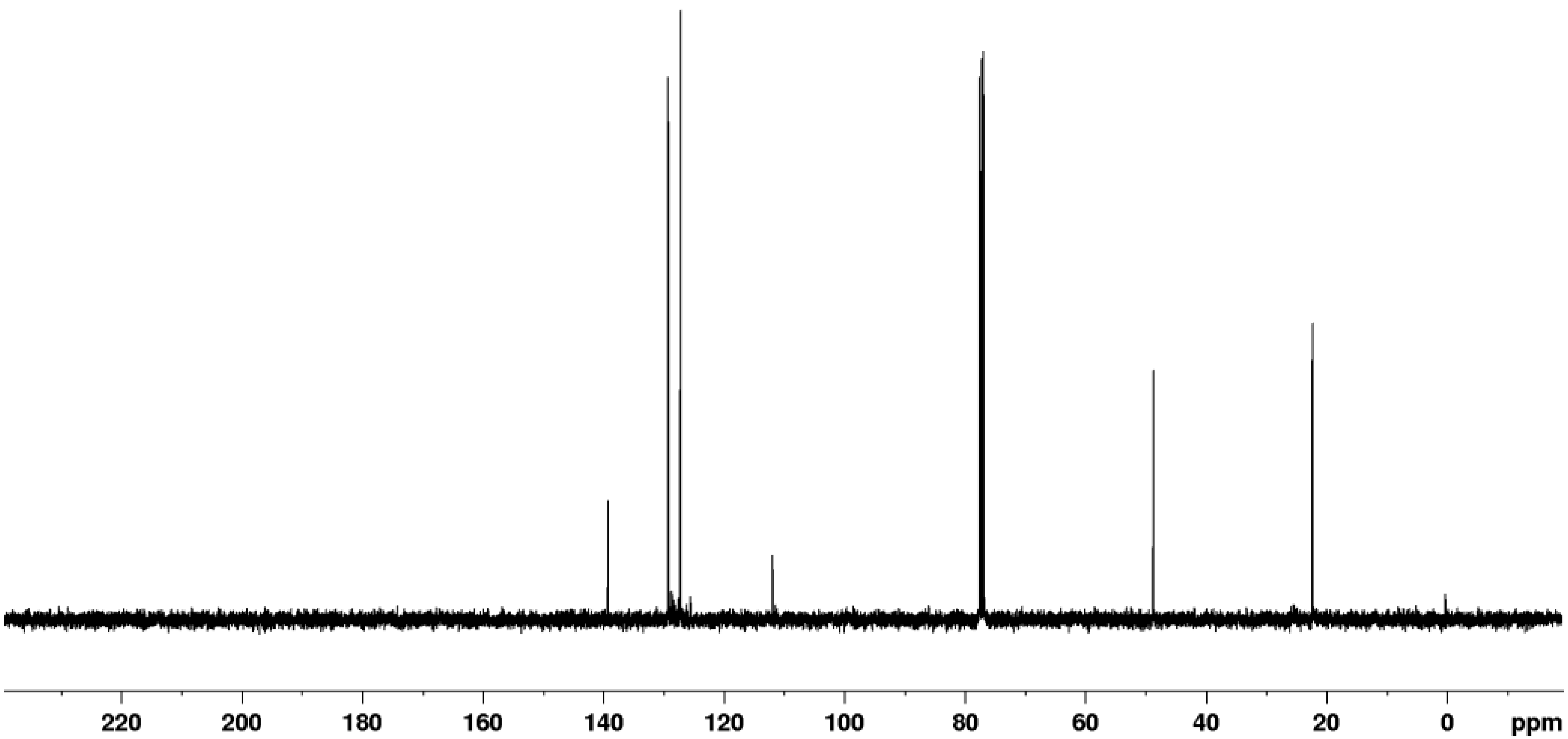

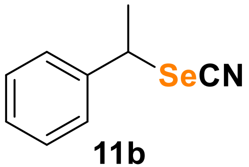

11b

^{1}H NMR ($CDCl_3$, 400 MHz)

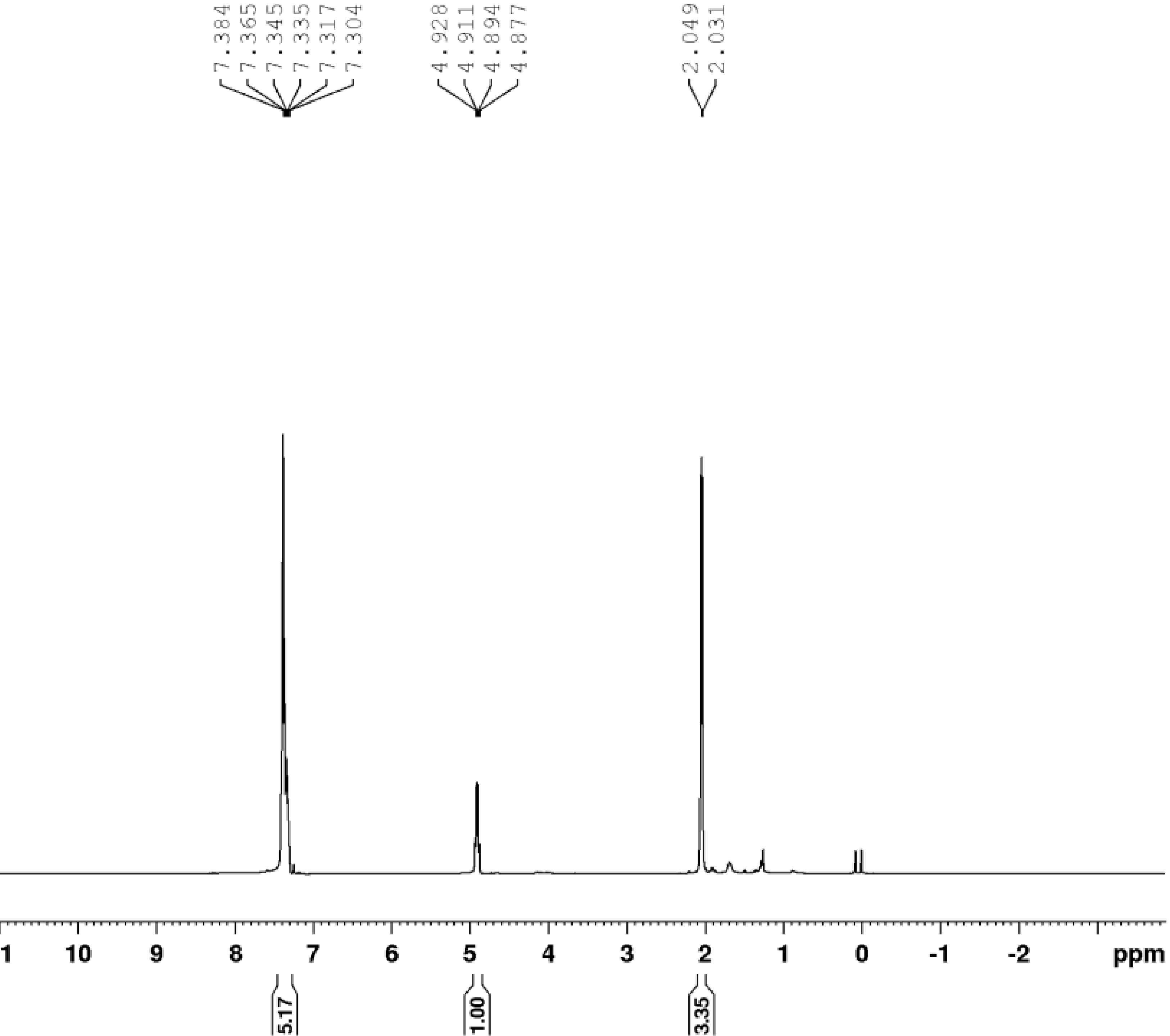

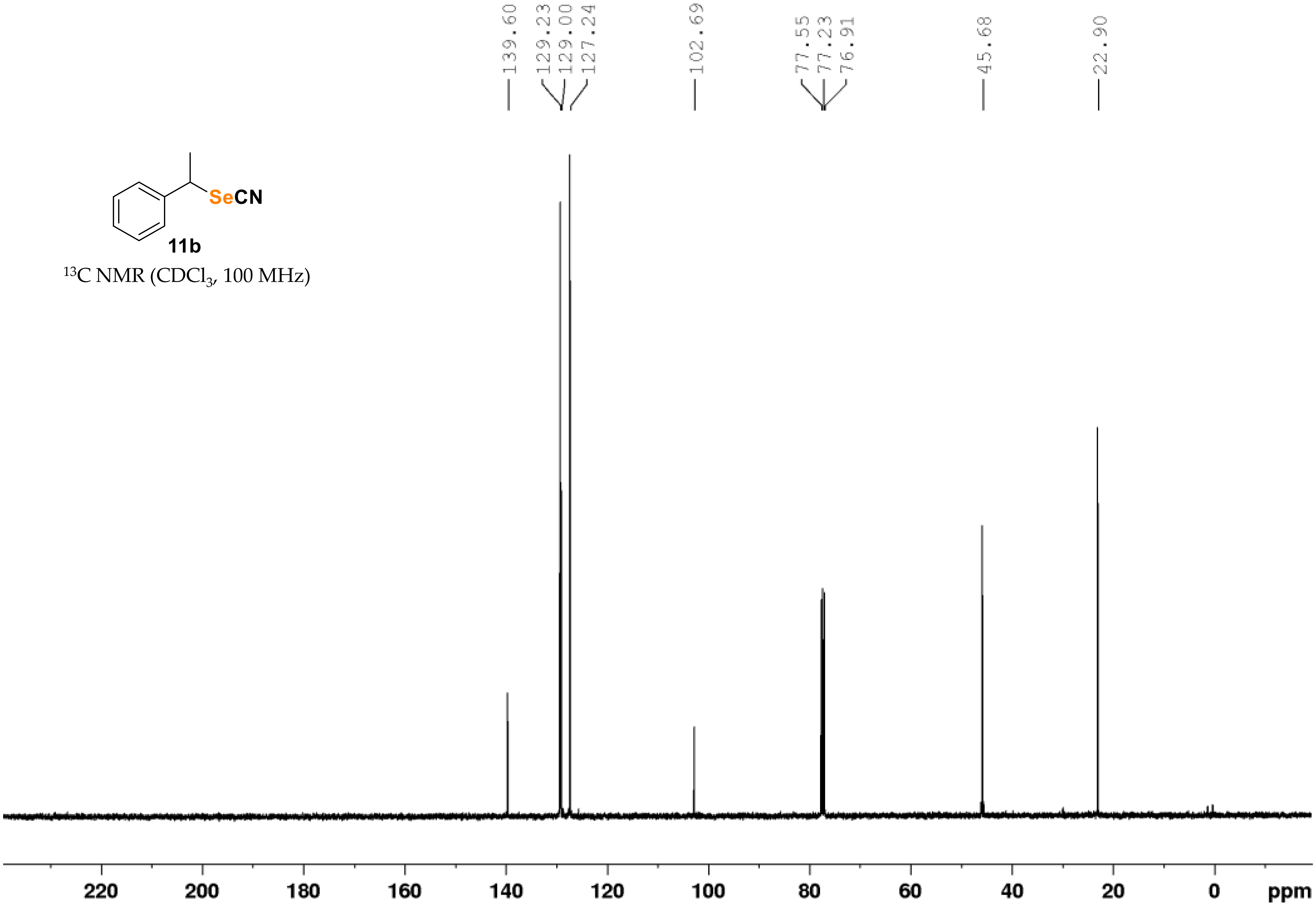

139.60
129.23
129.00
127.24
102.69
77.55
77.23
76.91
45.68
22.90
SeCN
11b
13C NMR (CDCl3, 100 MHz)
220
200
180
160
140
120
100
80
60
40
20
0
ppm

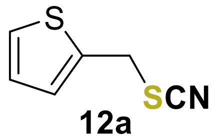

12a

^{1}H NMR ($CDCl_3$, 400 MHz)

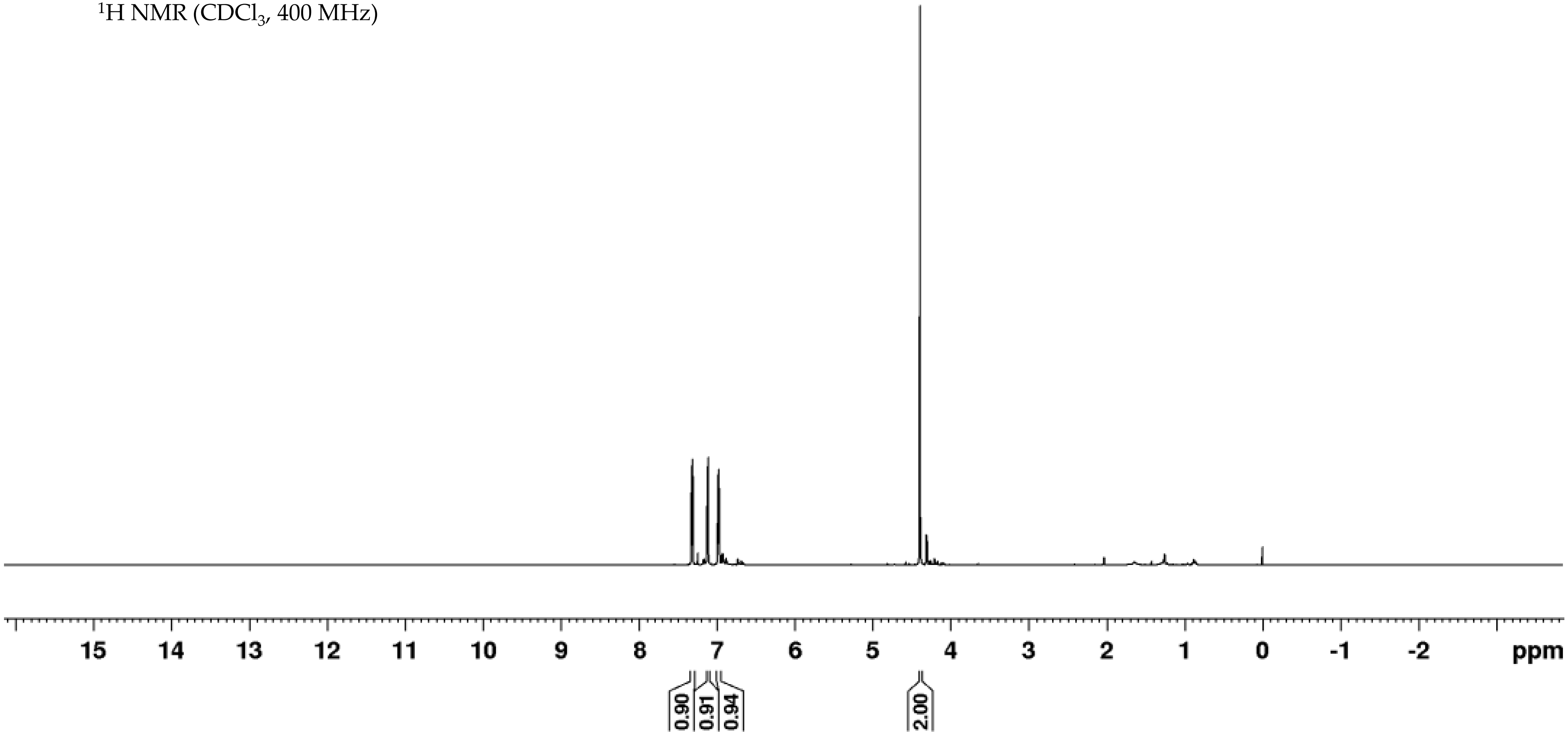

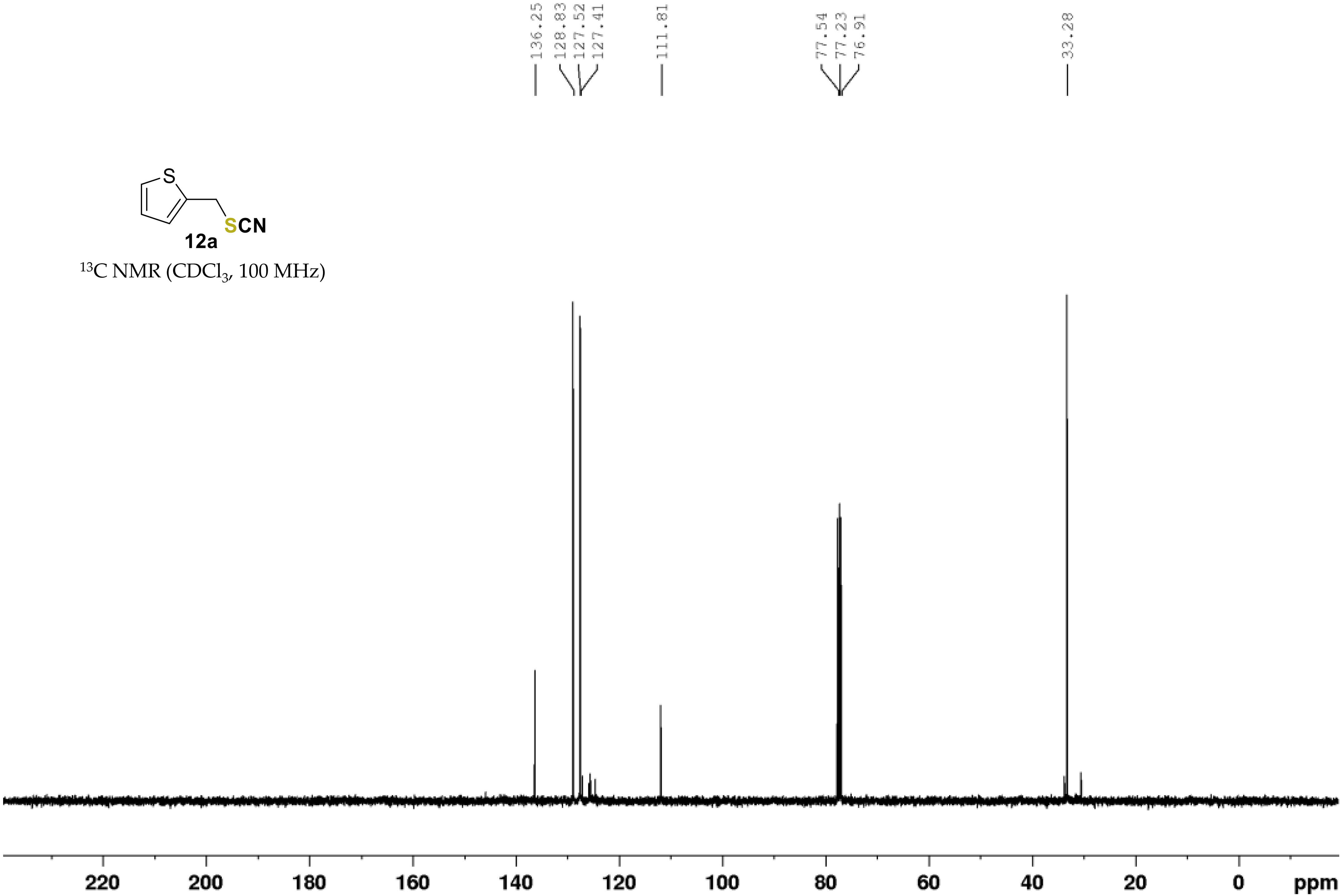

12a

^{13}C NMR ($CDCl_3$, 100 MHz)

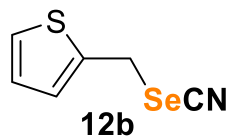

12b

^{1}H NMR ($CDCl_3$, 400 MHz)

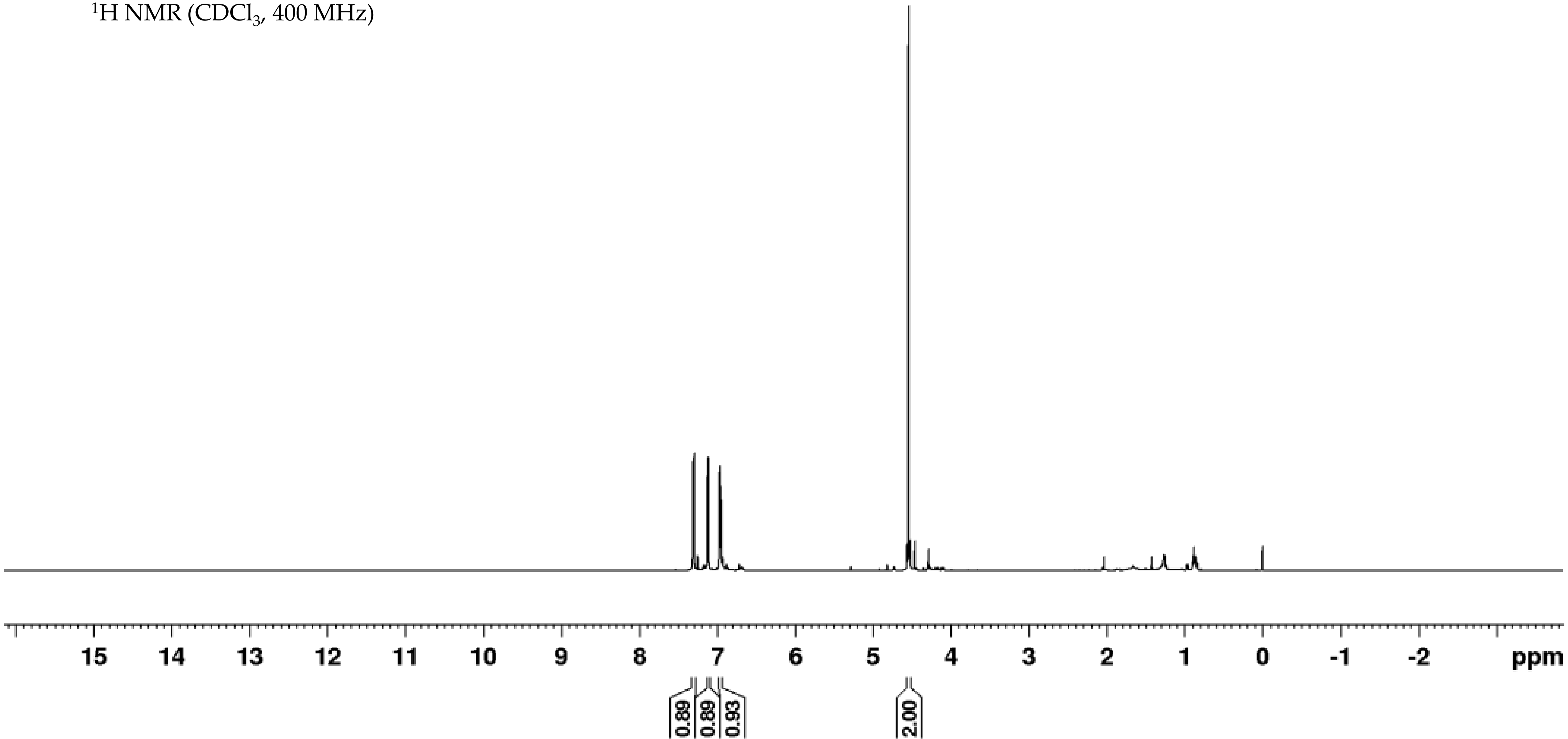

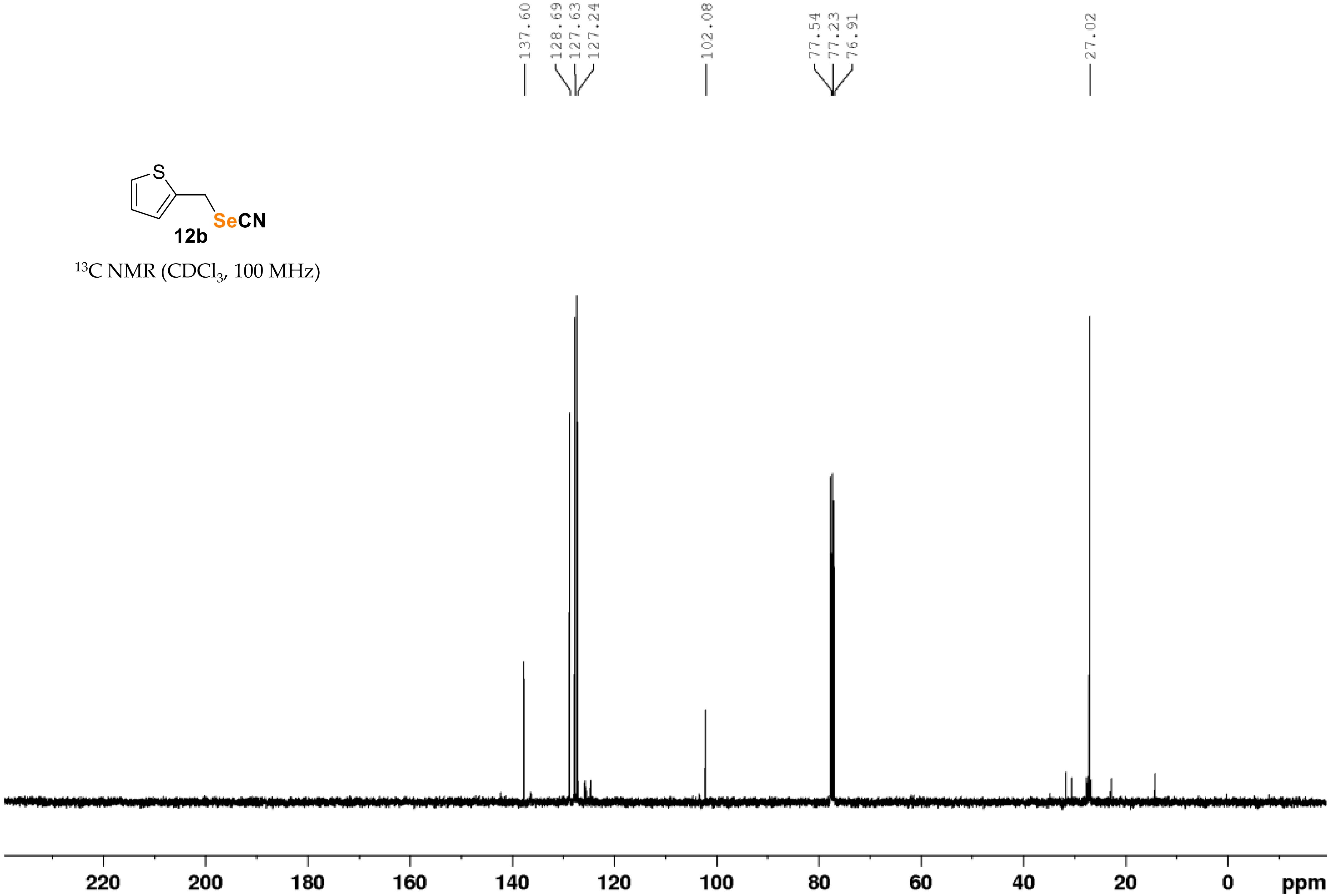
137.60
128.69
127.63
127.24
102.08
77.54
77.23
76.91
27.02
S
SeCN
12b
13C NMR (CDCl3, 100 MHz)
220
200
180
160
140
120
100
80
60
40
20
0
ppm

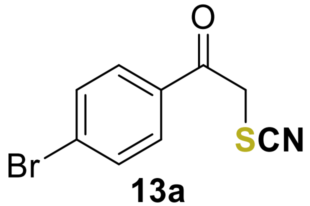

13a

^{1}H NMR ($CDCl_3$, 400 MHz)

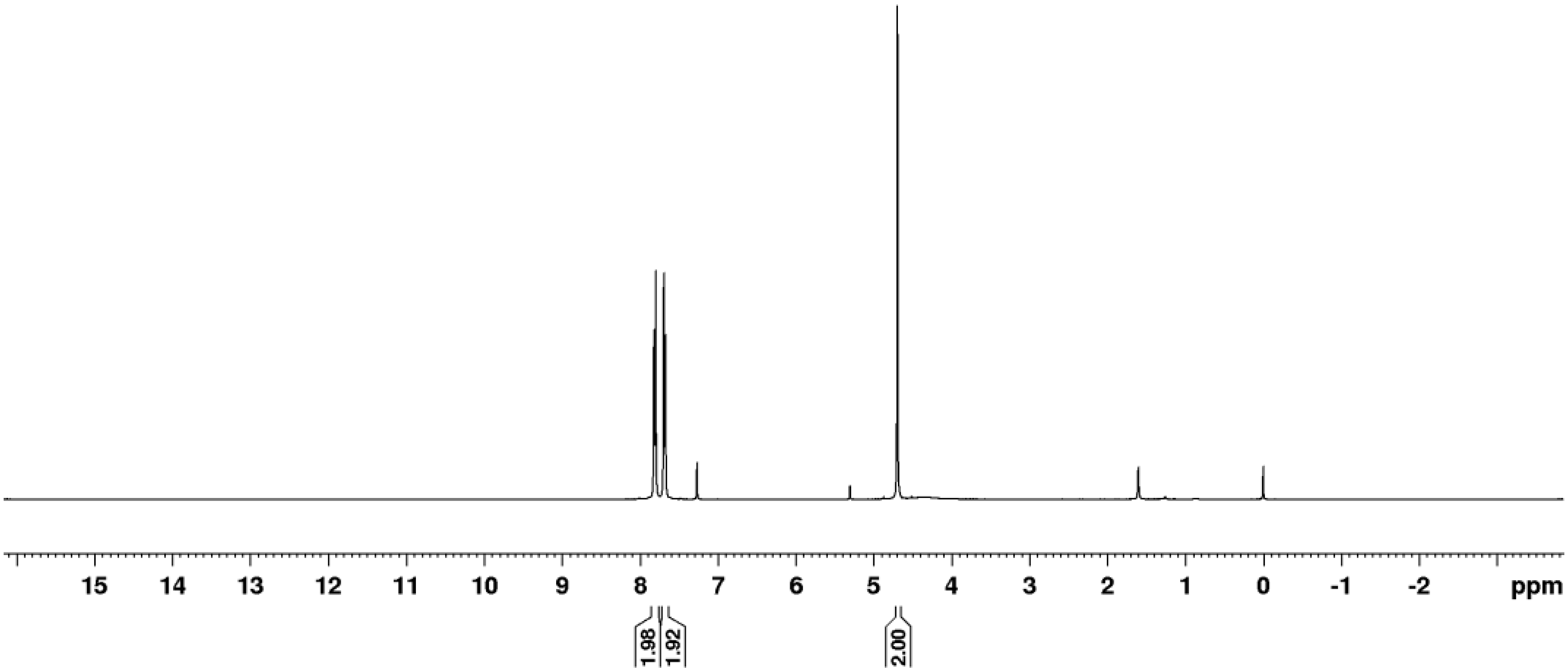

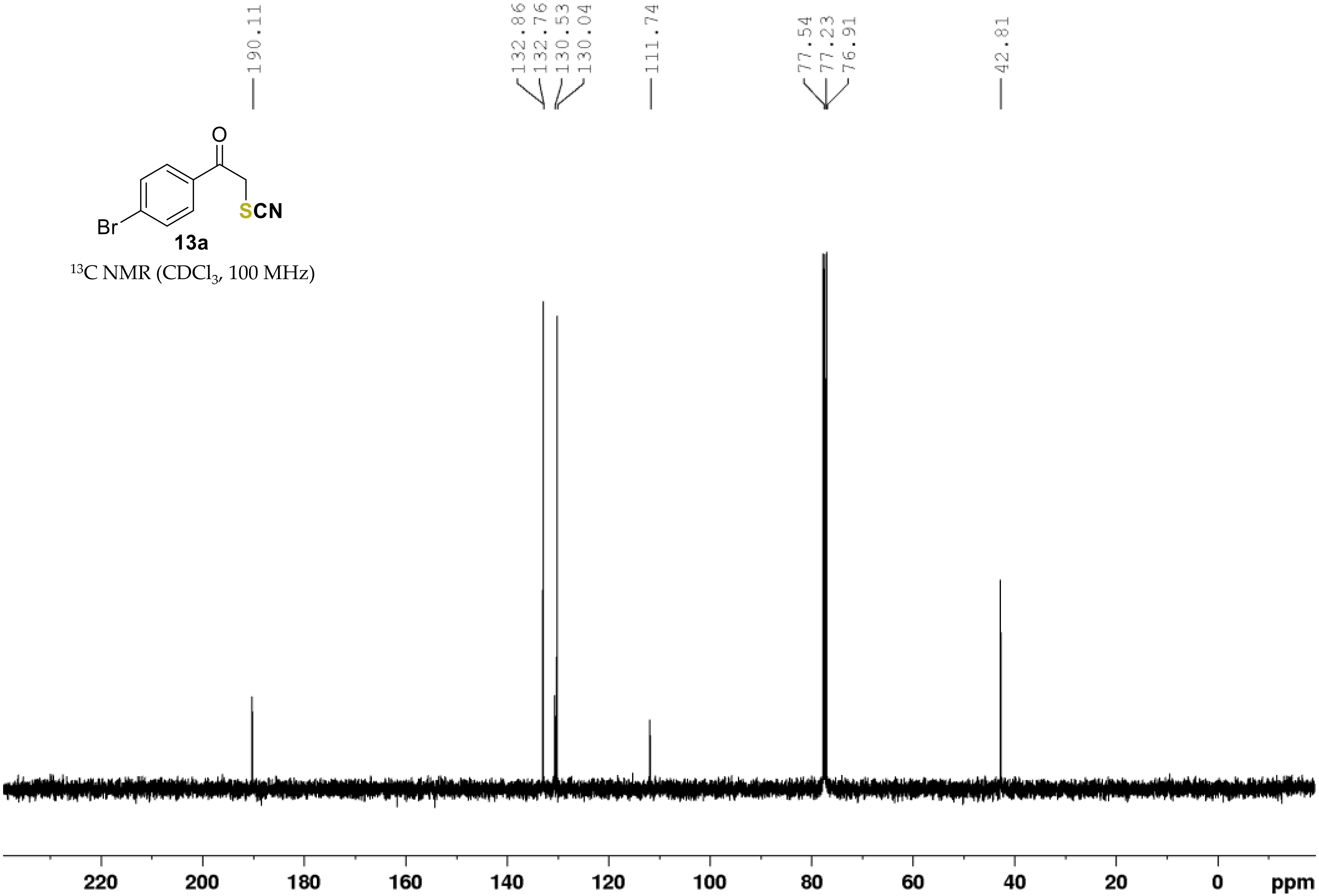
190.11
132.86
132.76
130.53
130.04
111.74
77.54
77.23
76.91
42.81
O
Br
SCN
13a
13C NMR (CDCl3, 100 MHz)
220
200
180
160
140
120
100
80
60
40
20
0
ppm

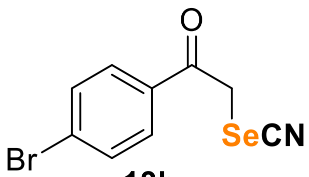

13b

^{1}H NMR ($CDCl_3$, 400 MHz)

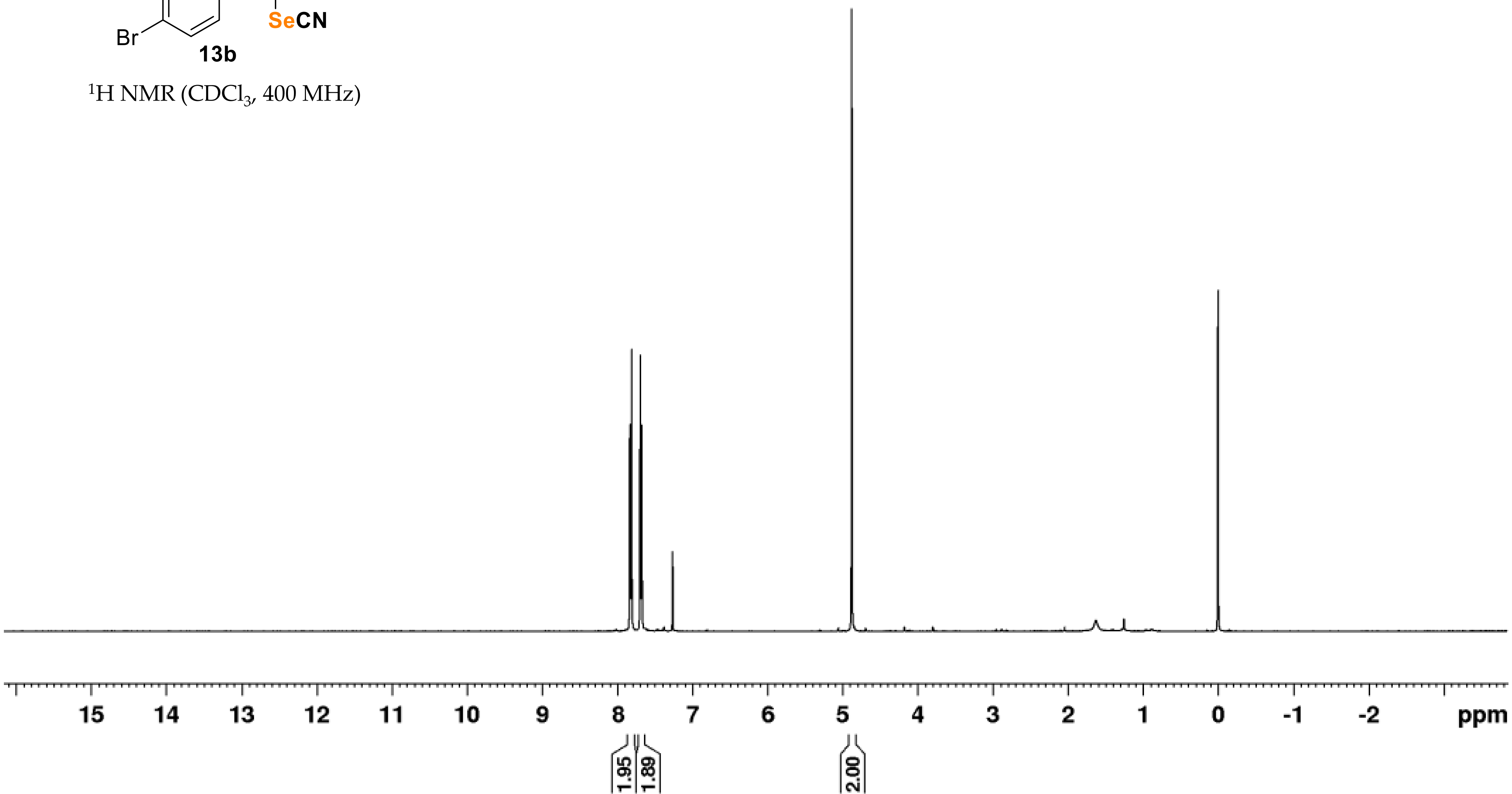

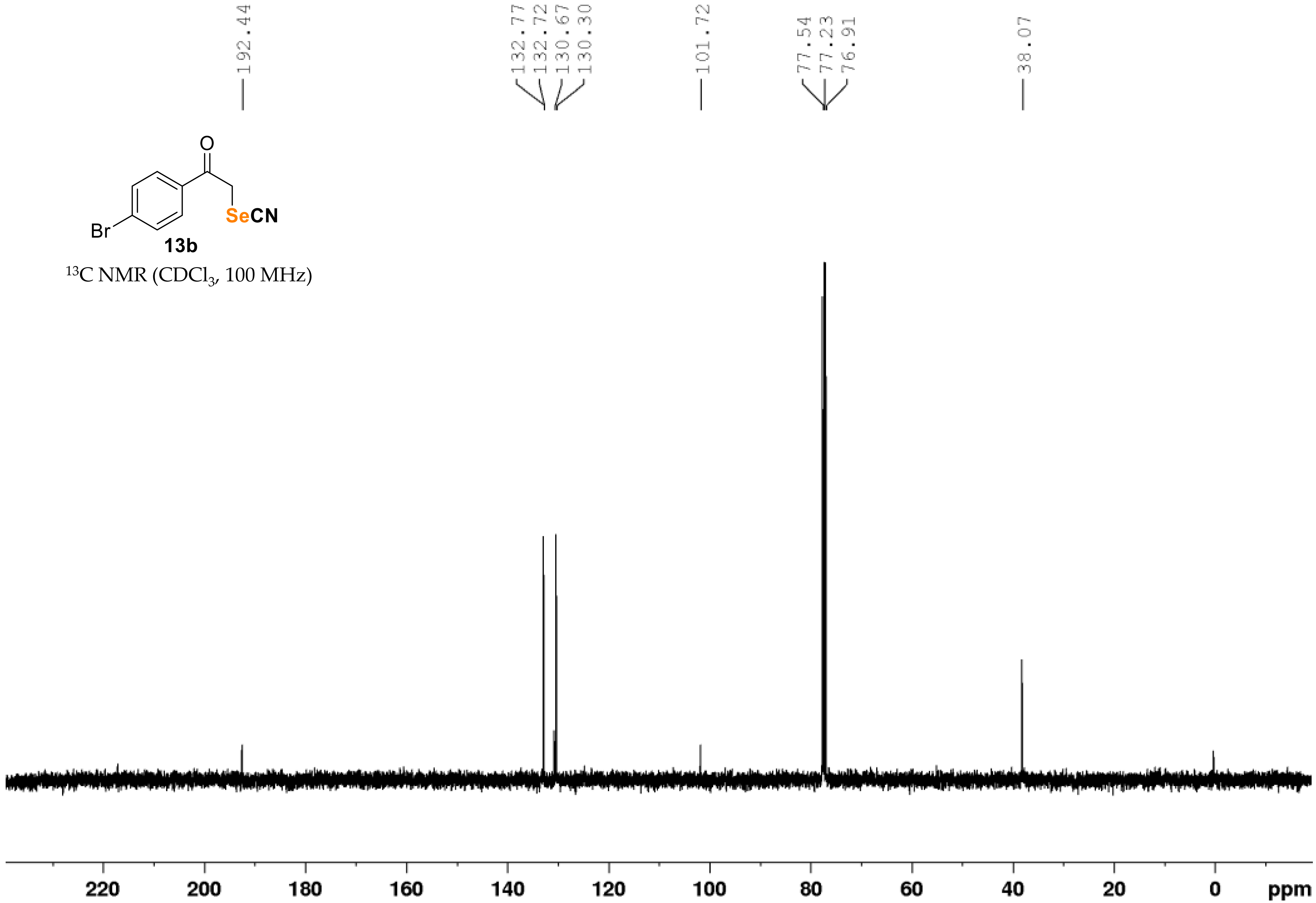
192.44
132.77
132.72
130.67
130.30
101.72
77.54
77.23
76.91
38.07
O
Br
SeCN
13b
^{13}C NMR ($CDCl_3$, 100 MHz)
220
200
180
160
140
120
100
80
60
40
20
0
ppm
